# *Aspergillus fumigatus* PolX1 is an early ancestor of vertebrate terminal deoxynucleotidyl transferases

**DOI:** 10.1093/nar/gkaf1497

**Published:** 2026-01-23

**Authors:** Najma Parveen, Sophia Steblina, Abhijit Behera, Caecilie M Benckendorff, Gavin J Miller, Katie E Davis, Purba Mukherjee

**Affiliations:** Department of Chemistry, University of York, Heslington YO10 5DD, United Kingdom; Department of Biological Sciences, Indian Institute of Science Education and Research, Kolkata, Mohanpur 741246, India; Department of Chemistry, University of York, Heslington YO10 5DD, United Kingdom; Department of Biological Sciences, Indian Institute of Science Education and Research, Kolkata, Mohanpur 741246, India; School of Chemical & Physical Sciences and Centre for Glycoscience, Keele University, Staffordshire ST5 5BG, United Kingdom; School of Chemical & Physical Sciences and Centre for Glycoscience, Keele University, Staffordshire ST5 5BG, United Kingdom; Department of Biology, University of York, Heslington YO10 5DD, United Kingdom; School of Biosciences, University of Sheffield, Sheffield S10 2TN,United Kingdom; Department of Chemistry, University of York, Heslington YO10 5DD, United Kingdom; Department of Biological Sciences, Indian Institute of Science Education and Research, Kolkata, Mohanpur 741246, India

## Abstract

X-family DNA polymerases (PolXs) perform essential roles in repair and maintenance of the genome. One branch of the PolXs have evolved to function as terminal transferases, extending DNA ends in a template-independent manner, unusual for polymerases. To date, template independence has been shown exclusively in metazoans. We analysed PolXs to determine the phylogenetic evolution of the terminal transferase function in fungal PolXs. We have identified and characterised a PolX from the saprophytic fungus *Aspergillus fumigatus*, named AfPolX1, that demonstrates inherent terminal transferase ability under physiologically relevant conditions. This is the first report for a fungal terminal deoxynucleotidyl transferase (TdT). Our findings indicate that template-independent ‘creative’ synthesis evolved earlier than previously thought and can be traced as far back as the early Polµ’s of multicellular fungi. We further show that like TdT, AfPolX1 is capable of introducing ribonucleotides and various nucleotides with 2′ ribose modifications, giving credence to the idea that the structural features necessary for PolXs observed promiscuous behaviour during template independence existed in the PolXs of early eukaryotes. Our findings suggest AfPolX1 as a promising candidate for use in enzymatic oligonucleotide synthesis.

## Graphical Abstract

**Graphical Abstract:**
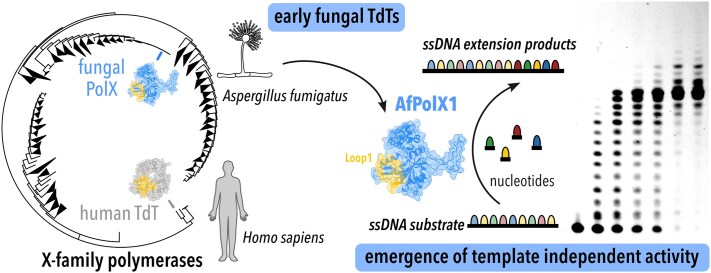

## Introduction

Maintenance and repair of genetic material is an essential endeavour for all domains of life. The X-family of DNA polymerases (PolXs) encode repair polymerases that carry out gap-filling functions required for base-excision repair and double strand break (DSB) repair through non-homologous end-joining (NHEJ) in non-dividing cells [1–5]. Vertebrates have four PolXs – β, λ, μ, and the terminal deoxynucleotidyl transferase (TdT) [6]. TdT performs the unusual function of efficient template-independent primer extension, called ‘creative synthesis’, [7] introducing sequence variability during V(D)J recombination with the consequence of diversifying the repertoire of antigen receptors [8, 9]. Polµ also shows a degree of terminal transferase activity [10], making it possible to convert incompatible DNA breaks into compatible double-stranded DNA (dsDNA) overhangs for NHEJ to move forward [9, 11, 12]. The functional overlap can be attributed to high sequence conservation between Polμ and TdT (43% sequence identity). In comparison, Pols β and λ are closer evolutionarily (34% identical in humans) [13, 14] and do not possess creative synthesis capability. Structurally, all PolXs share the catalytic core formed of fingers, palm, and thumb regions, linked to an N-terminal ‘8-kDa’ accessory domain that encodes 5′-deoxyribose 5′-phosphate-lyase function important in backbone sugar excision during repair. In most PolXs, except Polβ, an additional sequence and length variable N-terminal BRCT (BRCA1 C-terminal) domain is found, which mediates protein–protein interactions during NHEJ [15, 16].

In general, a combination of two features is used to identify PolXs that bear terminal transferase activities. Firstly, an unstructured region called loop1 or ‘lariat’ loop, located within the polymerase’s palm subdomain, is thought to act as a ‘pseudo-template’ facilitating nucleotide incorporation in the absence of template [17, 18]. Indeed, loop1 deletions of human TdT and Polμ lack template-independent synthesis ability [10, 19]. Second, in both Polμ and TdT, the position before the catalytic aspartates in the palm domain is a histidine conserved in vertebrates, H329 and H342 in human Polμ (HsPolµ) and TdT (HsTdT), respectively. Mutational studies show that swapping this position to alanine results in loss of TdT-like activity [20].

Exploiting terminal transferases has been crucial to the recent advances in chemoenzymatic oligonucleotide synthesis to establish sustainable, enzymatic processes [21–23]. Both TdT and its RNA equivalent, polyU polymerase, are being actively explored for template-independent enzymatic oligonucleotide synthesis strategies to manufacture nucleic acids [23, 24]. Several thermotolerant (up to 50°C) and salt tolerant TdT variants [25–27] have been evolved along with TdTs that successfully incorporate a host of nucleotide modifications including 3′-phosphate, 3′-ONH_2_, 2′-fluoro, and 2′-*O*-methyl (OMe), among others [26, 28–30]. New sources of TdT activity such as in a PolX from the tardigrade *Ramazzottius varieornatus*, an extremotolerant invertebrate [27], and a prokaryotic-Ago associated PrimPol [31] demonstrate other untapped origins of the terminal transferase activity and hint at the complex evolution path of this family of polymerases.

To date, the search for terminal transferases has been constrained to metazoans, potentially restricting the functional diversity that could be explored for evolution. Expanding to a broader sequence space necessitates a comprehensive understanding of early PolX variants harbouring terminal transferase activity. The repertoire of X-family polymerases available to an organism varies vastly: vertebrates have four PolXs (β, λ, μ, and TdT) [6], sea urchins (*Strongylcentrotus purpuratus*) encode three [14], and budding yeast (*Saccharomyces cerevisiae*) and fission yeast (*Schizosaccharomyces pombe*) each have a single X-family Pol, designated Pol IV [32, 33]. PolXs have also been identified in bacteria (*Bacillus, Deinococcus*), plant (*Arabidopsis*), fungi (*Coprinus*), and viruses (African swine fever virus) [14, 34, 35]. Intriguingly, fruit flies (*Drosophila*) and nematodes (*Caenorhabditis*) have none [14]. Phylogenetic analysis suggests TdTs evolved from a branch of Polµ-like precursors [14, 34] distant from the Pol IVs of unicellular fungi that have been characterized as lacking terminal transferase activity [32, 33]. This raises questions on the emergence and evolution of template independence. Crucially, this has not been explored in multicellular fungi. These fungi lacking Polβ and TdT instead encode two X-family polymerases that were precursors of the mammalian Pols µ and λ. We therefore considered whether these PolXs might harbour an ancient creative synthesis activity.

**Figure 1.**
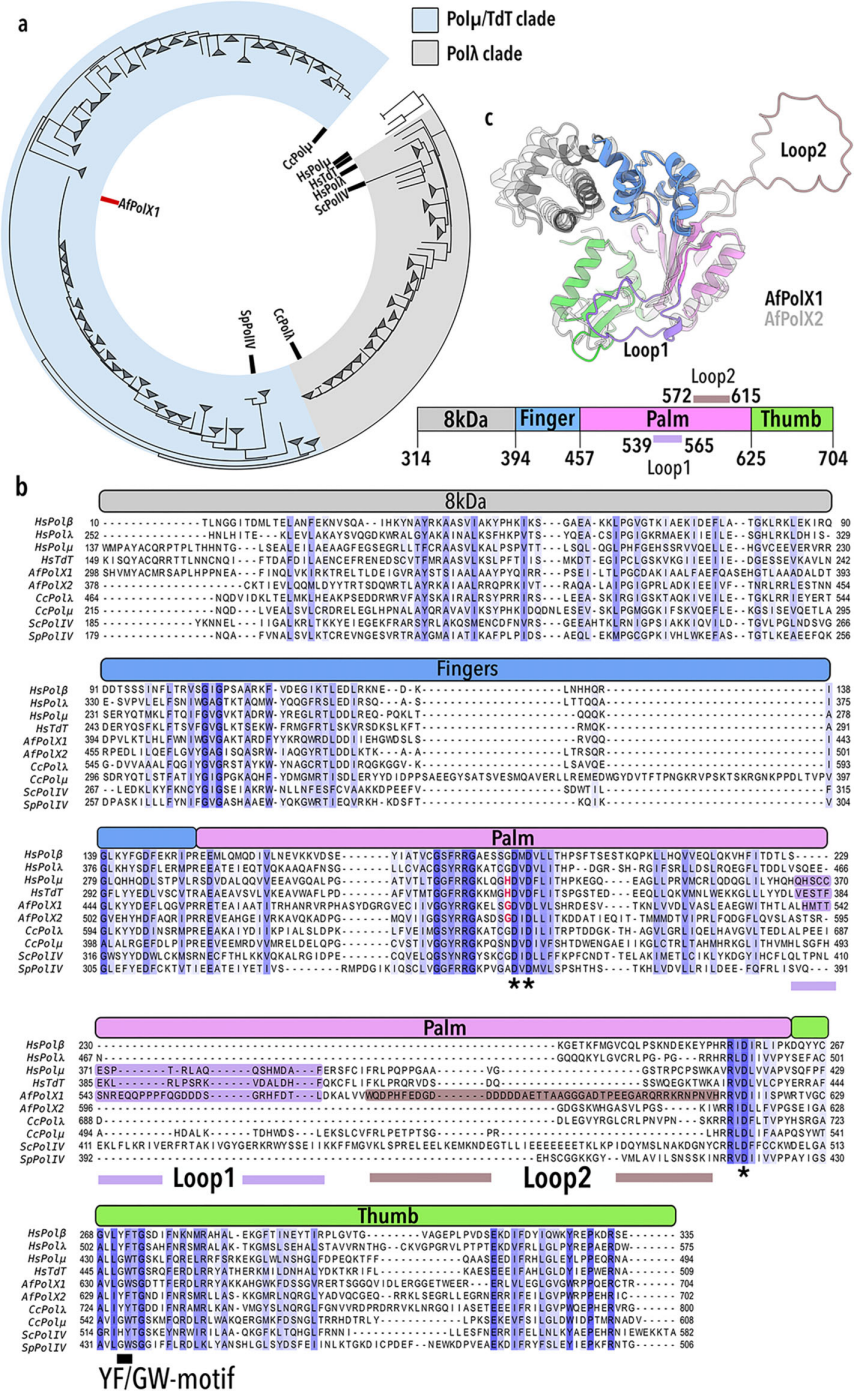
Evolutionary analysis of fungal X-family polymerases. (**a**) Phylogenetic tree inferred from the 8 kDa onwards regions of 2623 fungal PolXs. Analysis includes sequences of PolXs from humans (Hs), *Coprinus cinereus* (Cc), *S. cerevisiae* (Sc), and *Schizosaccharomyces pombe* (Sp), shown with black lines. *Aspergillus fumigatus* (Af) PolX1 is shown with a red line. See Supplementary File 1 for the full tree. (**b**) Multiple-sequence alignment (MSA) comparing the catalytic cores including the 8 kDa domains of PolXs. Sequences aligned are human Polβ (UniProt ID: DPOLB_HUMAN), Polλ (UniProt ID: DPOLl_HUMAN), Polμ (UniProt ID: DPOLM_HUMAN), and TdT (UniProt ID: TDT_HUMAN), *C. cinereus* Polμ (UniProt ID: Q5FBD6_COPCI) and Polλ (UniProt ID: A0PC13_COPCI), *S. cerevisiae* Pol IV (UniProt ID: DPO4_YEAST), and *S. pombe* Pol IV (UniProt ID: DPO4_SCHPO), along with AfPolX1 (UniProt ID: Q4X1T5_ASPFU) and AfPolX2 (UniProt ID: Q4WT56_ASPFU). Domain organisation shared among X-family polymerases comprising 8 kDa (grey), fingers (blue), palm (pink), and thumb (green) are represented as coloured bars above the sequence alignment. Overall conserved residues are coloured in shades of blue based on the percentage of conservation. Asterisks in the palm denote positions of the three catalytic aspartates. Loop1 region of the X-family members is shaded purple. Loop2 region in AfPolX1 is shaded in brown. Histidines in the palm region essential for TdT-like activity by HsTdT (H342) and HsPolµ (H329) with the corresponding glycines observed in AfPolX1 and AfPolX2 are highlighted in red. The YF/GW motif in the thumb harbouring the steric gate residue is shown underlined by a black line. (**c**) Predicted structures of AfPolX1 (coloured as per domain boundaries shown in panel (b) and the bar plot below) and AfPolX2 (light grey) are shown.

We identified PolXs homologous to the catalytic core of human TdT limited only to fungal sequences (Fig. 1a and Supplementary File 1). We then explored the existence of terminal transferase activity in the X-family polymerase revealed by our phylogenetic analysis from *Aspergillus fumigatus* (*Neosartorya fumigata*)*—*a thermotolerant, saprophytic, multicellular soil fungus known to cause the respiratory disorder aspergillosis in immune-compromised individuals [36]. We selected *Aspergillus* because it was found to occupy a currently unexplored sequence space within X-family polymerases (Fig. 1a) and since *A. fumigatus* has well-characterised thermotolerance [37]. These two factors made *A. fumigatus* PolXs promising starting candidates for future directed evolution efforts, provided template independence was identified.

The *A. fumigatus* genome encodes two genes belonging to the X-family, accession IDs XP_755218.1 and XP_752414.1, hereafter referred to as AfPolX1 and AfPolX2, respectively. Our analysis only identified AfPolX1 because of its similarity to human TdT. Biochemical characterisation of AfPolX1 demonstrates the presence of efficient terminal transferase activity in this early TdT variant. We further show its ability to efficiently incorporate various modified nucleotides. Taken together, our work illustrates that creative synthesis is an ancient ability that was already present in early eukaryotic PolXs.

## Materials and methods

### Phylogenetic analysis

To identify fungal specific X-family polymerases with terminal transferase characteristics, we performed a protein BLAST (with default matrix BLOSUM62) using the human TdT protein sequence (NP_004079.3) as query and limited results to records that included fungi (taxid:4751) while excluding all metazoan PolXs (taxid:33208). Only resultant hit sequences with *E*-values better than 1e−21 and sequence lengths between 400 and 2000 amino acids were aligned using Clustal Omega [38, 39]. Iterative sequence alignment included visual inspection to reduce outliers and lower sequence count to <3000. Control sequences also included in the alignment comprised previously characterized PolXs: human Polβ (UniProt ID: DPOLB_HUMAN), Polλ (UniProt ID: DPOLl_HUMAN), Polμ (UniProt ID: DPOLM_HUMAN), and TdT (UniProt ID: TDT_HUMAN), *Coprinus cinereus* Polμ (UniProt ID: Q5FBD6_COPCI) and Polλ (UniProt ID: A0PC13_COPCI), *S.cerevisiae* PolIV (UniProt ID: DPO4_YEAST), and *S.pombe* PolIV (UniProt ID: DPO4_SCHPO). Final MSA of full length PolX sequences revealed long extensions for many fungal PolXs. To exclude their influence on the evolutionary analysis of the catalytic core, only the aligned common region of X-family polymerases encompassing sequences of the catalytic core regions including the 8 kDa domain was used for phylogenetic tree inference as detailed below.

The phylogeny was inferred in MEGA [40] using maximum likelihood with a Le_Gascuel_2008 model [41]. Initial tree(s) for the heuristic search were obtained automatically by applying Neighbor-Join and BioNJ algorithms to a matrix of pairwise distances estimated using a Jones-Taylor-Thornton (JTT) model, and then the topology with the best log likelihood was selected. A discrete gamma distribution was used to model evolutionary rate differences among sites [5 categories (+G, parameter = 1.0864)]. The rate variation model allowed for some sites to be evolutionarily invariable ([+I], 0.35% sites). All positions with less than 95% site coverage were eliminated, missing data and ambiguous sites were allowed at any position (partial deletion option).

### Structure prediction and domain analysis

The genome of the multicellular fungi *A. fumigatus Af293* (Genome ID: GCA_000002655.1; NCBI Taxonomy ID: 330879) encodes for two uncharacterised putative X-family polymerases: PolX1 (accession ID: XP_755218.1; UniProt ID: Q4X1T5_ASPFU) annotated as ‘putative terminal deoxynucleotidyl transferase’ and PolX2 (accession ID: XP_752414.1; UniProt ID: Q4WT56_ASPFU) annotated as ‘putative Pol4’. ColabFold v1.5.5 (AlphaFold2 using Mmseqs2) [42] predicted tertiary structures of AfPolXs were compared with high-resolution structures available for human PolXs and murine TdT [18, 20, 43–45]. The well-demarcated domain boundaries of the mammalian PolXs were used to designate domains in AfPolX1 and AfPolX2. Structures were visualised and analysed using UCSF ChimeraX [46, 47].

### Cloning, expression, and purification of AfPolX1

A codon-optimised gene construct of the catalytic core (residues 297–704 of full length) of AfPolX1 was synthesised and cloned by GenScript Corp. (USA) into the pET28a(+) vector for protein overexpression in *Escherichia coli*. The expression plasmid for a mutant AfPolX1 with the glycine at position 501 mutated to histidine, hereon referred to as AfPolX1G501H-pET28a+, was obtained as a gift. In both constructs the N-terminus contains a hexahistidine tag followed by a tobacco etch virus (TEV) protease cleavage site. Plasmids were transformed into Rosetta™ 2 (DE3) (Merck, USA) strain of *E. coli*. Cultures were grown in terrific broth containing appropriate antibiotics at 37°C to an optical density (OD) of 0.8 before induction with 0.5 mM isopropyl ß-d-1-thiogalactopyranoside at 20°C for 16–18 h. Cells were harvested by centrifugation.

All the subsequent steps of protein purification were done at 4°C unless mentioned otherwise. The harvested cell pellets were suspended in buffer A (20 mM HEPES, pH 7.5, 500 mM NaCl, and 20 mM imidazole) supplemented with ethylenediaminetetraacetic acid (EDTA)-free protease inhibitor (Roche, USA) and lysozyme. Cells were lysed by sonication on ice and the cell debris was separated by centrifugation at 10 000 × *g* for 1 h. The supernatant was syringe filtered through a 0.45 µm filter prior to loading onto a Ni^2+^-charged HiTrap Chelating HP column or HiTrap IMAC FF (Cytiva, USA) pre-equilibrated with buffer A. The column was washed with a high-salt wash buffer (20 mM HEPES, pH 7.5, 1 M NaCl, and 20 mM imidazole) to remove contaminants before re-equilibration in buffer A. A linear gradient of buffer A containing imidazole from 20 mM to 1 M was used to elute AfPolX1. Fractions containing AfPolX1 were buffer exchanged into TEV digestion buffer containing 20 mM HEPES (pH 7.5), 100 mM NaCl, 5% glycerol, 0.1 mM EDTA, and 1 mM dithiothreitol (DTT) to eliminate imidazole before adding TEV protease for 6x-His tag removal at 4°C overnight. The digested untagged AfPolX1 was centrifuged at 15 000 × *g* for 10 min to remove protein aggregates. The supernatant obtained was then concentrated and diluted in buffer A to reduce EDTA and DTT concentrations before loading on a Ni^2+^-charged HiTrap Chelating HP or HiTrap IMAC FF column pre-equilibrated with lysis buffer. Untagged AfPolX1 was obtained in the flowthrough and buffer exchanged into storage buffer containing 50 mM HEPES (pH 7.5), 200 mM NaCl, 15% glycerol, 0.1 mM EDTA, and 1 mM DTT, before concentrating with a Sartorius Vivapsin 30kD cut-off filter (Sartorius, Germany). Protein concentration was calculated using extinction coefficient ɛ_280 _= 76 890 M^−1^cm^−1^, followed by flash-freezing in liquid nitrogen and storage at −80°C.

### DNA substrates

All DNA oligonucleotides enumerated in Supplementary Tables S1 and S2 were acquired from Integrated DNA Technologies (USA). Upstream primers for the DNA oligos were synthesised with a 6-carboxyfluorescein (FAM) label at the 5′ end. All DNA substrates in Supplementary Table S1 were prepared by annealing in a buffer containing 10 mM HEPES and 50 mM NaCl by heating for 20 min at 95°C, followed by a gradual cooling to room temperature.

### Nucleoside triphosphates

Deoxyribonucleoside triphosphates (dNTPs: dATP, dTTP, dGTP, dCTP) and ribonucleoside triphosphates (rNTPs: ATP, UTP, GTP, CTP) for activity assays were acquired from Cytiva, USA. 2′-fluoro- and 2′-OMe-CTPs and GTPs were procured from Jena Bioscience, Germany, and diluted with nuclease-free water to desired concentration before use. All other nucleotides used in the study were synthesised as described below (also see Supplementary Methods).

### Synthesis of nucleoside triphosphates

All reactions were conducted using anhydrous solvents, under an atmosphere of N_2_, unless otherwise stated. Flash column chromatography was performed using silica gel, high purity grade, pore size 60 Å, 230–400 mesh particle size, 40–63 μm particle size (Sigma–Aldrich). Thin layer chromatography (TLC) was performed using precoated 0.25 mm 60 F254 silica gel plates (Merck). Visualisation was achieved using UV light (λ = 254 nm). All high-resolution mass spectra were measured at the EPSRC National Mass Spectrometry Facility at Swansea University, UK. NMR spectra were recorded on a Bruker Avance 400 spectrometer.

### Nucleoside 5′-O-triphosphates

To an oven-dried multi-necked round bottom flask equipped with a magnetic stirrer bar, nucleoside analogue (1.0 equiv.) was added, and the reaction flask was evacuated and refilled with N_2_ three times. The nucleoside was suspended in PO(OMe)_3_ (0.75 M), and the mixture was cooled to −20°C (ice/NaCl). POCl_3_ (1.2 equiv.) was added dropwise, and the reaction mixture was allowed to warm to –10°C and maintained between −10°C and 0°C. Once complete or majority consumption of the starting material was observed *via* analytical high pressure (or high performance) liquid chromatography (HPLC) [see general analytical strong anion exchange (SAX) HPLC method], the reaction mixture was cooled to −10°C, and a −20°C solution of bis(tributylammonium) pyrophosphate (2.0 equiv.) in MeCN (0.4 M relative to the pyrophosphate) and Bu_3_N (6.0 equiv.) was added. The reaction mixture was allowed to slowly warm to 0°C over 1 h. Once complete consumption of the monophosphate intermediate was observed *via* analytical SAX HPLC, the reaction mixture was quenched by the addition of H_2_O, and the mixture was stirred for a further 0.5–1 h. The aqueous mixture was washed with dichloromethane (DCM) three times, and the aqueous phase was concentrated *in vacuo* at 30°C. The material was purified *via* flash column chromatography on silica gel, followed by ion exchange chromatography using DEAE Sepharose (see purification of NTPs).

### Nucleoside 5′-O-[α-thio]triphosphates

To an oven-dried multi-necked round bottom flask equipped with a magnetic stirrer bar, nucleoside analogue (1.0 equiv.) was added, and the reaction flask was evacuated and refilled with N_2_ three times. The nucleoside was suspended in PO(OMe)_3_ (0.5 M) and pyridine (2.0 equiv.), and the mixture was cooled to −20°C (ice/NaCl). PSCl_3_ (1.2 equiv.) was added dropwise, and the reaction mixture was allowed to warm to –10°C and maintained between −10°C and 0°C. Once complete or majority consumption of the starting material was observed *via* analytical HPLC (see general analytical SAX HPLC method), the reaction mixture was cooled to −10°C, and a −20°C solution of bis(tributylammonium) pyrophosphate (2.0 equiv.) in MeCN (0.4 M relative to the pyrophosphate) and Bu_3_N (6.0 equiv.) was added. The reaction mixture was allowed to slowly warm to 0°C over 1 h. Once complete consumption of the monophosphate intermediate was observed *via* analytical SAX HPLC, the reaction mixture was quenched by the addition of H_2_O, and the mixture was placed in the freezer overnight. Upon thawing, the aqueous mixture was washed with DCM three times, and the aqueous phase was concentrated *in vacuo* at 30°C. The material was purified *via* flash column chromatography on silica gel, followed by ion exchange chromatography using DEAE Sepharose (see purification of NTPs).

### Purification of NTPs

A column without a frit was packed first with cotton wool, followed by a thin layer of sand, a filter paper, and finally with silica gel in *^i^*PrOH. The crude material was dissolved in a small amount of H_2_O or *^i^*PrOH and loaded onto the column. The column was eluted following a gradient of 0%–50% NH_4_OH/*^i^*PrOH. Fractions containing the product (confirmed *via* TLC analysis, 2:3 *^i^*PrOH/NH_4_OH) were pooled and concentrated *in vacuo* at 30°C. The concentrated residue was loaded onto a column packed with DEAE Sepharose and eluted following a gradient of 0%–50% 1M NH_4_HCO_3(aq)_/H_2_O. The fractions containing the product (confirmed *via* TLC analysis, 2:3 *^i^*PrOH/NH_4_OH) were pooled and concentrated *in vacuo* at 30°C. To remove excess NH_4_HCO_3_ salt, the residue was dissolved in a minimum amount of H_2_O and MeOH was added before concentrating *in vacuo* at 30°C. This step was repeated until a stable mass of product was achieved. The product was transferred to a Falcon™ tube and lyophilised.

### Thermal shift assay

Thermal shift assays were performed using 5 µM AfPolX1 wild-type (WT) and 5X SYPRO orange in a buffer containing 50 mM HEPES (pH = 7.5) and 50 mM or 150 mM NaCl. The reaction mixture was heated from 25°C to 95°C in the Stratagene Mx3005P instrument for 71 cycles, with a 1°C increment per cycle (1°C/30 s), and fluorescence measured at 585 nm. Reactions were performed in triplicate, and the mean normalised fluorescence spectra with error (calculated as standard deviation) were plotted against temperature. Melting temperature (*T*_m_) was calculated from the normalised graph using the JTSA server [JTSA: Bond, PS (2017) at http://paulsbond.co.uk/jtsa].

### Gap-filling assays

All reactions were performed at 37°C in reaction buffer containing 25 mM HEPES (pH 7.5), 50 mM sodium acetate, and 10 mM magnesium acetate. Reactions containing 4 µM AfPolX1 were preincubated with 150 nM DNA substrate in the reaction buffer before addition of 1 mM respective nucleotides (dNTP or rNTP). At appropriate time points, reactions were quenched with an equal volume of quenching buffer comprising 90% formamide, 50 mM EDTA, and 0.025% sodium dodecyl sulphate (SDS). Samples were heated to 95°C for 20 min before loading on a 23% acrylamide (19:1)-1× TBE denaturing gel containing 6 M urea to separate extension products from the unextended primer. Gels were imaged using Bio-Rad Chemidoc (SYBR Gold filter) and quantitated using Bio-Rad Image Lab Software. The fraction of primer extension was calculated and plotted against time using GraphPad Prism software (v. 10.5.0). The product formation (*y*) at different times (*t*) at which the reaction was quenched were fit to the equation:


\begin{eqnarray*}
y = A\left[ {1 - {{{\rm e}}^{( - kt)}}} \right] + c
\end{eqnarray*}

where *A* is the amplitude of product formation, *k* is the observed single turnover rate constant, and *c* is a constant.

### Template-independent nucleotide incorporation assays

All template-independent extension assays were performed at 37°C in reaction buffer containing 50 mM HEPES (pH 7.5) and 50 mM sodium acetate. Samples containing 4 µM AfPolX1 (either purified WT or G501H mutant) or calf thymus TdT (New England Biolabs, USA) were preincubated with 25 nM FAM labelled single-stranded DNA (ssDNA) substrate in reaction buffer for 10 min on ice before reactions being initiated as detailed below for each specific assay. At desired time points, reactions were quenched with an equal volume of quenching buffer comprising 90% formamide, 50 mM EDTA, and 0.025% SDS. Samples were heated to 98°C for 10 min and loaded on a 15% acrylamide (19:1)-1X TBE denaturing gel containing 6 M urea to separate extension products from the unextended primer. The gels were imaged on an Amersham Typhoon 5 (Cytiva, USA) laser-based scanner using blue light (excitation wavelength = 495 nm; emission wavelength = 520 nm), allowing detection of the FAM labelled primers.

### Multiple nucleotide extension assays

All multiple nucleotide extension assays were performed at 37°C. Reactions containing 4 µM AfPolX1 (WT/G501H) were preincubated with 25 nM FAM labelled ssDNA substrate in reaction buffer containing 50 mM HEPES (pH 7.5) and 50 mM sodium acetate for 10 min. Reactions were initiated by addition of 10 mM magnesium acetate or manganese chloride or 10 mM magnesium acetate with 0.25 mM cobalt chloride and 2.5 µM unlabelled trap dsDNA (Supplementary Table S1) and 1 mM each of all four nucleotides.

### Single nucleotide primer extension assays

Reactions were initiated by the addition of 1 mM of dNTP, rNTP, or modified nucleotides (2′-fluoro, 2′-OMe, 2′-*O*-methoxyethyl, or phosphorothioate) with 2.5 mM magnesium acetate or manganese chloride or 2.5 mM magnesium acetate with 0.25 mM of cobalt chloride and 2.5 µM of unlabelled trap dsDNA. The gel bands were quantitated using Image Quant software (Cytiva, USA) and the fraction extended was calculated using the following equation: 


\begin{eqnarray*}
&&{\mathrm{Fraction\ Extended}}= {(avgIE-avgIB)\over {(avgIE-avgIB)+(avgIU-avgIB)}}
\end{eqnarray*}

where avgIE is the average intensity of the extended primer, avgIB is average background intensity, and avgIU is average intensity of the unextended primer.

## Results

### Phylogenetic analysis of TdT homologues in fungi

Primary sequence alignment of the X-family polymerases of multicellular fungi indicates that fungal PolXs are longer than their counterparts in mammals and unicellular yeast. This also holds true for the PolXs from *C. cinereus* (Cc), the only other characterised multicellular fungal system [48]. A key contributor to the larger PolX is the longer N-terminal extension (Supplementary Fig. S1) that presumably contributes to the BRCT domain of these polymerases. Our subsequent analysis focused only on the catalytic cores including the 8 kDa domain since this spans the most conserved regions of X-family polymerases. We compared AfPolX1 and AfPolX2 with the human PolXs and the yeast PolIVs (Fig. 1b)—both at the sequence level and structurally, relying on AlphaFold predicted tertiary structures for those polymerases that lacked experimentally determined structural information (Supplementary Fig. S2a).

Both AfPolX1 and X2 adopt the domain architecture of known TdT and Polµ structures [1, 17, 18, 20]. Overall AfPolX1’s structure was predicted with high confidence (pTM = 0.88), having a plDDT score >90 for most structured regions of the polymerase (Supplementary Fig. S2a). AfPolX1 has two distinct, sizable loop regions designated as loops 1 (27 residues) and 2 (44 residues) embedded in its palm (Fig. 1c). The loop2 region is noticeably shorter in the other PolXs compared in this study (Fig. 1b). In keeping with the prediction that X2 is a Polλ homolog, loop1 in AfPolX2 is considerably shortened (10–11 residues). Both AfPolXs, however, lack the histidine identified to be critical in Polµ and TdT for stabilising the incoming nucleotide and single-stranded primer terminus as substrates within the polymerase active site—in the absence of templating information [20]. Instead, a glycine occupies this position (G501 and G552 in X1 and X2, respectively) (Fig. 1b, residues highlighted in dark red).

The two PolXs of *A. fumigatus* share just 22% identity (Supplementary Fig. S2b and c), an indication that AfPols X1 and X2 possibly evolved independently. Consistent with this analysis, we found that AfPolX2 indeed showed higher identity to Polλ and its homologues, 38% and 36.5% identity with CcPolλ and HsPolλ, respectively, implicating *A. fumigatus* PolX2 functions as Polλ *in vivo*.

Four PolXs of fungal origin have been previously tested for terminal transferase activity—ScPolIV and SpPolIV, the sole X-family polymerases encoded in the unicellular yeast*S. cerevisiae* and *S. pombe*, respectively, and the Pols λ and µ of the basidiomycete *C. cinereus* [32, 33, 48]. None of these PolXs have been reported to have the capacity for template-independent creative synthesis. In our resulting phylogeny, AfPolX1 was recovered in an evolutionarily distant position from each of these PolXs (Fig. 1a), occupying sequence space uncharacterised in the search for a terminal transferase (see Supplementary File 1 for the phylogenetic tree in full). We, therefore, proceeded to biochemically characterise the catalytic core of AfPolX1.

### AfPolX1 can perform template-dependent DNA synthesis

We overexpressed and purified the catalytic core of AfPolX1 (residues 298–704) (Fig. 1b and  Supplementary Fig. S3a). To ascertain activity, we tested the primer-template utilisation by AfPolX1 (Supplementary Fig. S3b) and found it demonstrates extension on a 5′-overhang substrate. Reaction conditions including pH, temperature, and Na^+^ concentration were then optimised by performing end-point multiple nucleotide extension assays on substrates D3 and D4 (Supplementary Fig. S3c and d). Despite evidence that *A. fumigatus* tolerates temperatures of up to 65°C [49, 50], the catalytic core of AfPolX1 appears to undergo unfolding at temperatures around 40°C (Supplementary Fig. S3e), as observed in thermal melt assays [51]. We first evaluated whether AfPolX1 can perform any gap-filling activity attributed to PolXs.

### AfPolX1 performs gap filling with poor sugar selectivity

**Figure 2.**
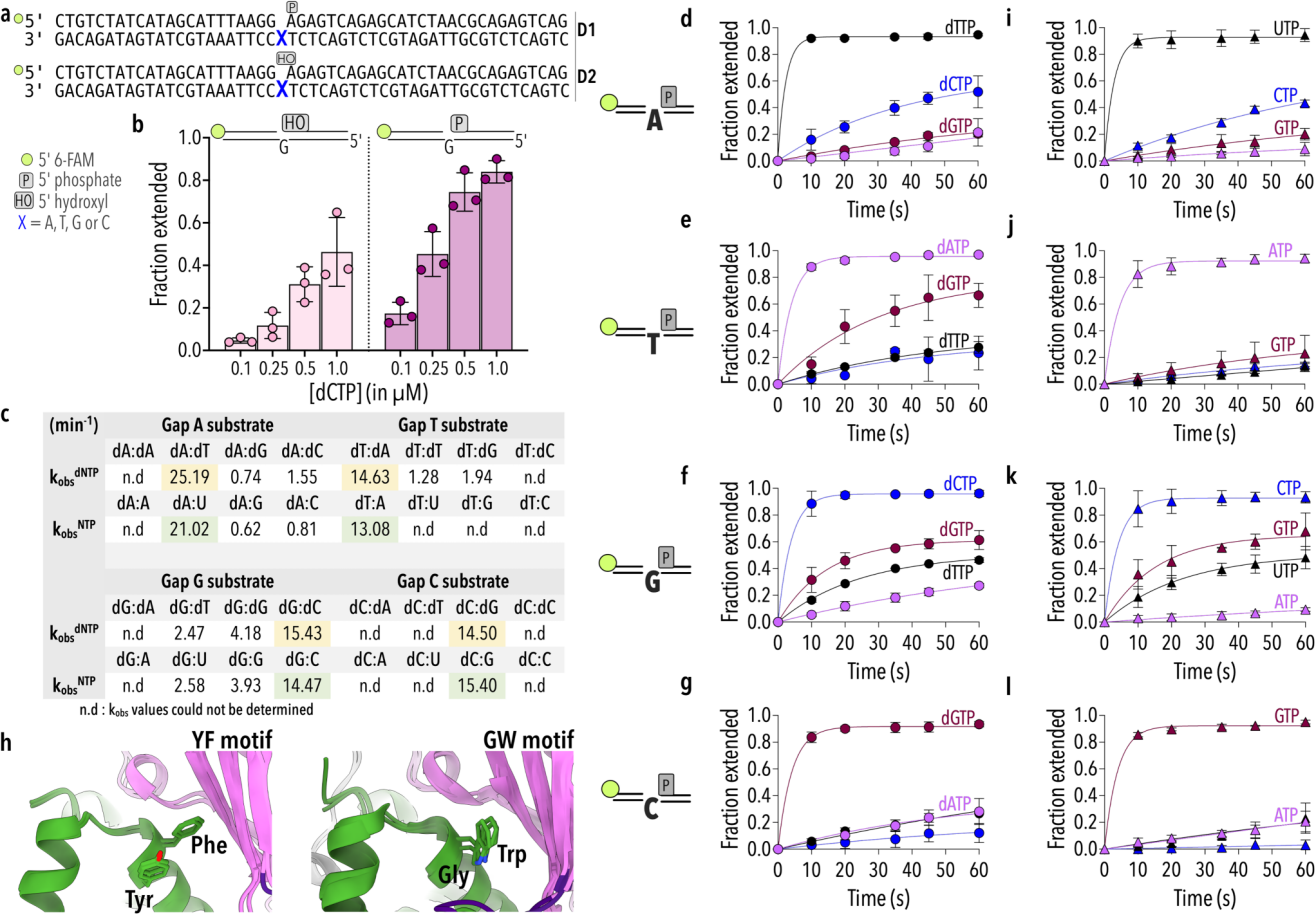
Gap-filling activity of AfPolX1. (**a**) DNA substrates used for AfPolX1 biochemical characterization. The templating base position is highlighted in blue. (**b**) Substrate specificity of AfPolX1 between single nucleotide gapped substrates D2 (5′-OH downstream, light pink, left panel) and D1 (5′-phosphate, dark pink, right panel). The templating base in both substrates is dG. Substrate utilisation was compared at incoming dCTP concentrations between 0.1 and 1 µM. (**c**) Misincorporation propensity of AfPolX1 against 1-nt gapped substrates. The table shows observed rates of incorporation (*k*_obs_) for each dNTP or rNTP on each of the four different gapped substrates. All *k*_obs_ for correct nucleotide incorporations are denoted in yellow. Matched rNTP rates are highlighted in green. Data represent at least two independent replicates. ‘n.d.’ refers to *k*_obs_ values that could not be determined. Incorporation of dNTP (**d**–**g**; shown as filled circles) and rNTP (**i**–**l**; shown as filled triangles) by 4 µM AfPolX1 on each of the four possible 1-nt gapped substrates (150 nM) in the presence of 1 mM of each nucleotide (dATP or ATP in pink, dTTP or UTP in black, dGTP or GTP in maroon, and dCTP or CTP in blue). Cartoon depicting gapped substrate corresponding to each row of panels is shown to the left. Error bars represent SD of three biological replicates. (**h**) Panels comparing YF-motif present in Pols β and λ with GW motif in Polµ and TdT present within the thumb domain (green) oriented towards the active site in the palm domain (pink) restrict rNTP incorporation.

Gap filling is a key feature of repair polymerases involved in base and nucleotide excision repair pathways. As *A. fumigatus* lacks a Polβ homolog, AfPols X1 and X2 are expected to stand in for this activity. We used a substrate with a single nucleotide (1-nt) gap (Fig. 2a) mimicking an intermediate of DNA nick repair that is likely to be filled by PolXs. Pols β, λ, µ, and SpPolIV have been shown to exhibit a strong preference for 5′-phosphorylation of the downstream primer of the gap as representative of damages *in vivo* [33, 52–55]. When comparing correct nucleotide incorporation by AfPolX1 opposite the 1-nt gap either with a 5′P or with a 5′OH, we found that AfPolX1 also preferred 5′-phosphorylation at this position, becoming most apparent at limiting concentrations of dNTP (Fig. 2b). So, all subsequent gap-filling experiments were completed using the 5′-P substrates.

Next, we varied the templating base (A, T, G, or C) within the 1-nt gapped substrates (D1) and compared correct and incorrect dNTP incorporation by AfPolX1 in the gap (Fig. 2c and d–g). Correct nucleotide incorporation proceeded at comparable rates for all the substrates (*k*^dTTP^_obs _= 25.19 min^−1^, *k*^dATP^_obs _= 14.63 min^−1^, *k*^dCTP^_obs _= 15.43 min^−1^, *k*^dGTP^_obs _= 14.5 min^−1^) (Fig. 2c). However, the overall incorrect nucleotide addition was slowed by nearly an order of magnitude, with one exception. On a gap templating a dT, dGTP misincorporation was only 7.5-fold slower (*k*^dGTP^_obs _= 1.94 min^−1^) than the correct incorporation of a dA (Fig. 2c and e). G–T mispairs appear to be favoured in the AfPolX1 active site as gap G substrates also accept dTTP (Fig. 2f). Intriguingly, the G-dG misincorporation is more pronounced, happening ∼1.7-fold faster compared to dT incorporation.

Polµ’s propensity to misincorporate dGTP opposite a gapped T has been previously observed for human Polµ [56]. Based on 1- and 2-nt gapped substrates bound ternary structures of human Polµ, two residues, K438 and Q441, have been proposed to influence the active site microenvironment and promote misincorporation [56]. While the corresponding residues are not conserved in AfPolX1 (being a T638 and E641, respectively), the polymerase nonetheless catalyses dGTP misincorporation.

The rNTP pool within a cell can be up to six orders of magnitude higher compared to dNTP levels [57–59]. Replicative polymerases [60] and certain repair polymerases—like Pols β and λ—often contain bulky aromatic residues, such as tyrosine and phenylalanine, within their active sites that enforce sugar selectivity (Figs 1b and 2h). In PolXs, the backbone carbonyl oxygen in tyrosine sterically clashes with the 2′-OH of an incoming rNTP, preventing misincorporation [61, 62]. TdT and Polµ instead have a ‘GW motif’ (Figs 1b and 2h), where steric gating residues are replaced with glycine and tryptophan, removing any hindrance to rNTP binding within the active site.

It remains unclear how any preferential recognition of dNTPs over rNTP occurs in the absence of steric selectivity, given the considerable concentration differential in cells. In fact, using mouse embryonic fibroblasts, Pryor *et al.* have shown that rNTP misincorporation *in vivo* is common during break repair by NHEJ, being mediated by Polµ and TdT, and represents an important feature for downstream break ligation [12]. Our sequence analysis and structural comparisons of AfPolX1 revealed a GW motif, implicating an increased propensity for rNTP incorporation. In the context of the gapped DNA substrates, we observed robust, template-dependent addition of rNTPs (Fig. 2i–l). For each substrate, the cognate incoming rNTP was incorporated at rates directly comparable with their dNTP counterparts (*k*^UTP^_obs _= 21.02 min^−1^, *k*^ATP^_obs _= 13.08 min^−1^, *k*^CTP^_obs _= 14.47 min^−1^, *k*^GTP^_obs _= 15.40 min^−1^) (Fig. 2c). In all cases, base pairing alone determined incorporations as there was a clear decrease in the rate of incorporation for unmatched rNTP additions (e.g. CTP/GTP/ATP on a Gap-A substrate) that mirrored misincorporations with dNTPs (Fig. 2, comparison of panels left with right). An exception to this were the gap T:dGTP misincorporations (Fig. 2e) that were not observed as with GTP (Fig. 2j).

### AfPolX1 is an active template-independent nucleotidyl transferase

**Figure 3.**
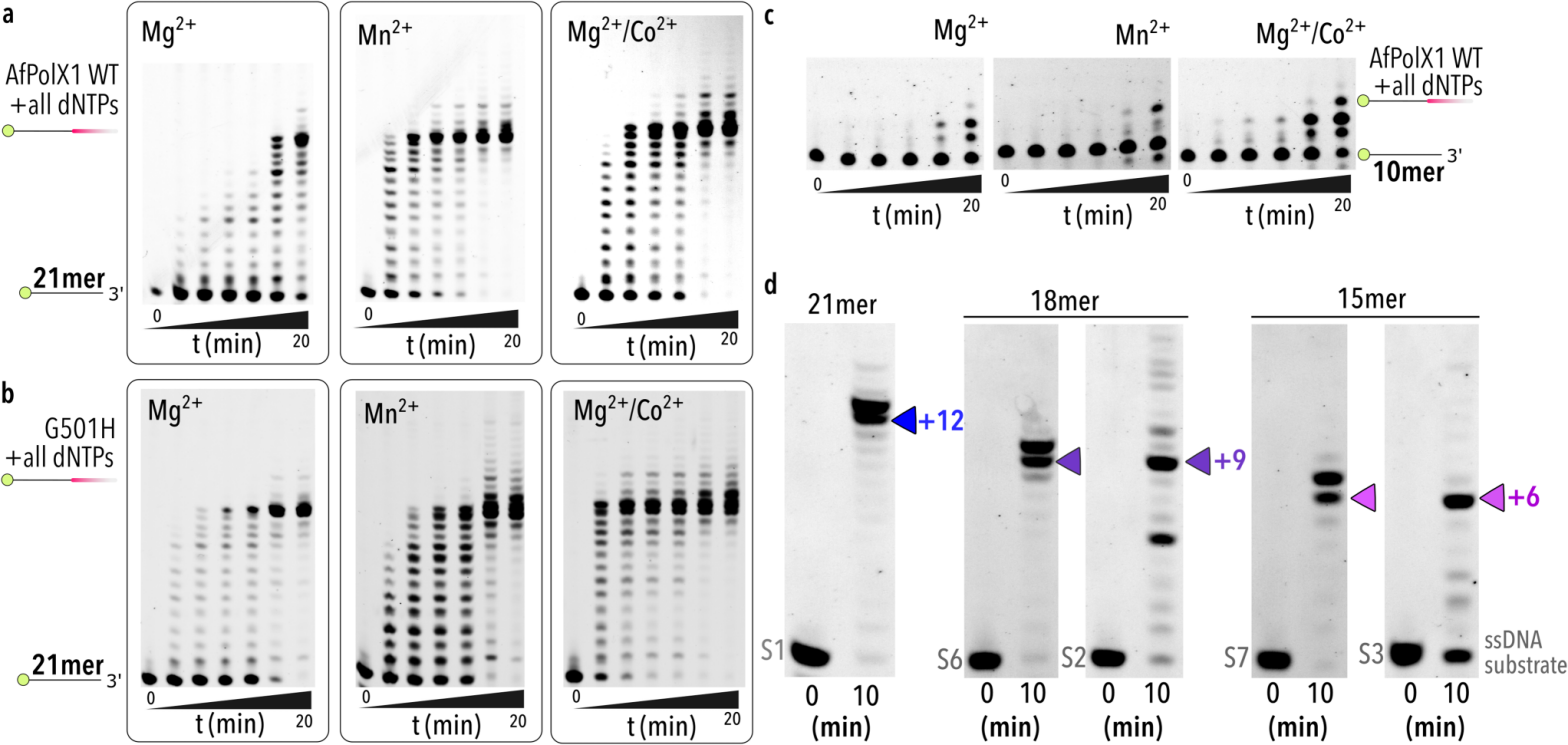
Template-independent nucleotidyl transferase activity of AfPolX1. Comparison of primer extension activity by 4 µM (**a**) AfPolX1 WT or (**b**) AfPolX1 G501H mutant on 25 nM 21mer ssDNA (substrate S1) in the presence of 1 mM each of all four dNTPs and 10 mM of divalent Mg^2+^, Mn^2+^, or Mg^2+^ supplemented with 0.25 mM Co^2+^ (Mg^2+^/Co^2+^). Cartoons represent unextended and extended primer alongside gel, with pink shaded region depicting newly formed ssDNA. Reactions were quenched 0.25, 0.5, 0.75, 1, 5, and 20 min after addition of divalent. (**c**) Gels comparing template-independent primer extension from a 10mer fluorescently labelled primer by WT AfPolX1 in the presence of 1 mM of each dNTP and 10 mM of Mg^2+^, Mn^2+^, or Mg^2+^/Co^2+^ as shown. Reactions were carried out for 0.5, 0.75, 1, 5, and 20 min. (**d**) ssDNA extension by AfPolX1, with all dNTPs present, show pausing at distinct stall points indicated with blue, purple, and pink triangles alongside gels for products from 21mer, two 18mers, and two 15mers, respectively. Conditions for extension are identical to that in panel (a) using Mg^2+^/Co^2+^. ssDNA substrates are as shown in grey below with sequences provided in Supplementary Table S2.

To investigate whether AfPolX1 is an early homologue of TdT encoded by *A. fumigatus*, we tested the template-independent nucleotide incorporation ability of AfPolX1 (Fig. 3). The active site DxD aspartates of vertebrate TdT are flanked by a histidine (Fig. 1b, red bold residues) that has been implicated in helping grip both the flexible ssDNA primer end and the incoming nucleotide, aiding the nucleophilic attack by the 3′-OH during template-independent nucleotide incorporation [20]. In AfPolX1, this position is occupied by glycine (G501) (Fig. 1b). We compared wild-type AfPolX1 to a point mutant where the G501 was switched to histidine, AfPolX1 G501H, and were surprised to find both wild-type (Fig. 3a) and the G501H mutant (Fig. 3b) polymerases capable of robust ssDNA extension. TdT shows improvements in template-independent activity in the presence of divalent transition metals such as Co^2+^ and Mn^2+ ^[7, 18]. In both AfPolX1 variants, magnesium alone was sufficient to observe template-independent incorporations that were enhanced when a combination of Mg^2+^ and Co^2+^ was provided. To rule out the possibility that transient spurious secondary structures formed by the 21mer (*T*_m _∼ 28°C) did not result in template-dependent priming, we tested AfPolX1 with a 10mer ssDNA (*T*_m _∼ 17°C) derived from the 21mer and retaining an identical 3′ end. We found that multiple non-templated extensions were still observed and became prominent at earlier time points with Mg^2+^/Co^2+^ (Fig. 3c). To our knowledge, this represents the first report of template-independent primer extension in a fungal PolX and provides evidence of the emergence of terminal transferase activity within the X-family polymerase scaffold in early eukaryotes.

Overall, G501H appeared marginally more active than the wild-type (Fig. 3a), indicating that positioning the histidine strategically within the active site boosts terminal transferase activity but is dispensable for terminal transferase function in fungal PolXs. Presumably, evolutionary pressures to retain and enhance terminal transferase activity—owing to its essential role in DNA repair functions—have driven the introduction of the histidine into active sites of vertebrate orthologs [20].

We observed that AfPolX1 reached a unique stall point when extending the 21mer (Fig. 3a and d), not seen in TdTs. Shorter primers (10 and 12mers) did not generate sufficiently long products to display this distinct stall pattern (Supplementary Fig. S4), the 15 and 18mers did (Fig. 3d). By testing primers of identical length but different sequences, we established that stalling occurred at precise product lengths (Fig. 3d), being length-dependent rather than primer sequence-dependent. Intrigued by the unique pattern of template-independent activity by AfPolX1, we proceeded to further characterise this with Mg^2+^.

### Terminal transferase activity relies on sequence context

**Figure 4.**
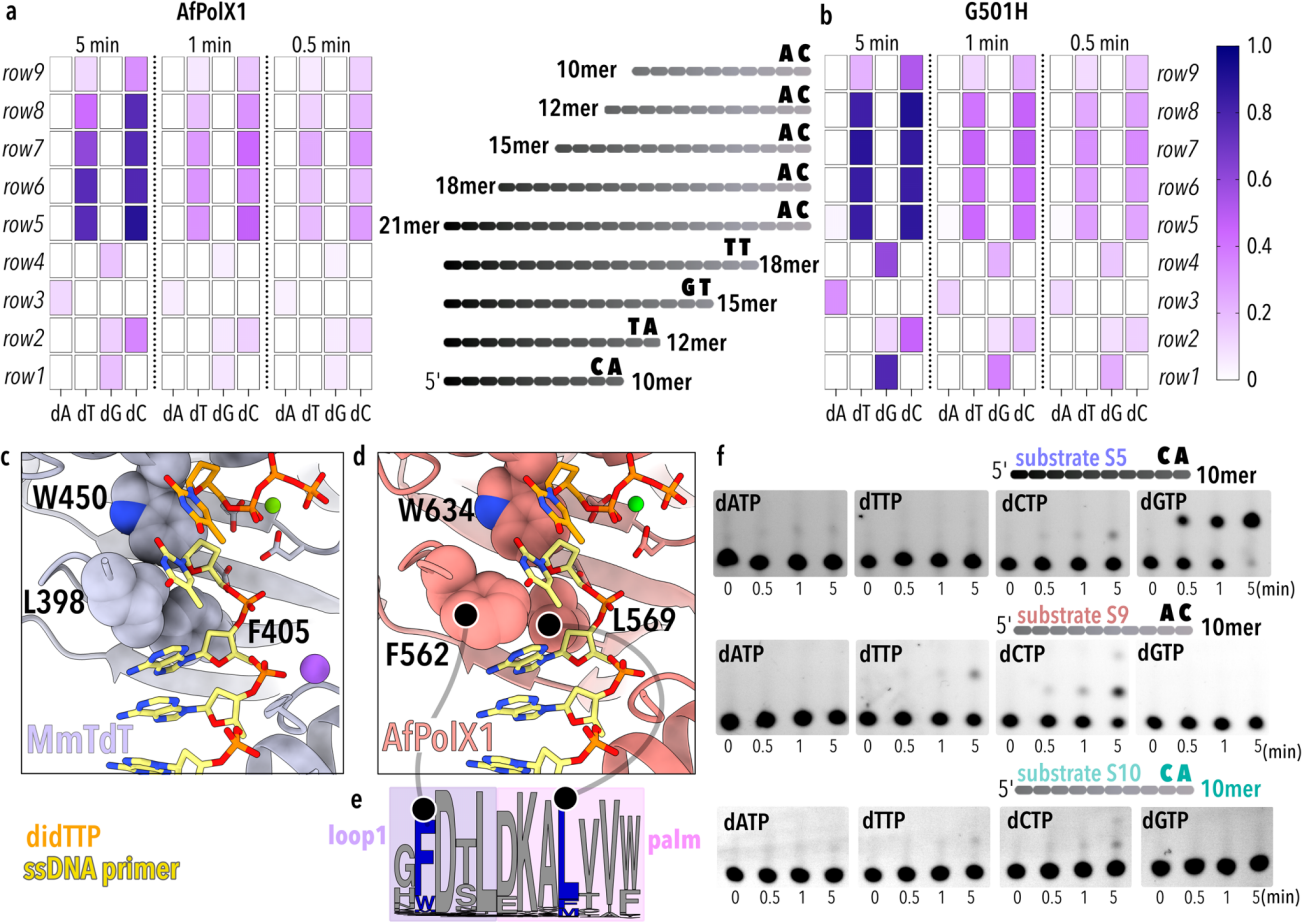
Heatmaps comparing the incorporation of individual dNTPs during template-independent extension in the presence of Mg^2+^ for 0.5, 1, and 5 min by (**a**) AfPolX1 WT and (**b**) G501H to single-stranded FAM-labelled primers of varying lengths and 3′ sequence ends (as shown in schematic between panels). Scale bar depicts fraction extension ranging from white (0, no extension) to dark blue (1.0, complete primer utilisation). Cartoon depictions of 10–21mer primers with shaded grey ovals indicating individual bases along the primer and identity of the two nucleotides from the 3′ end are shown between panels (a) and (b). (**c**) Hydrophobic Leu/Phe residue pair between the loop1 and palm in mouse TdT (MmTdT, mauve) creates a wedge that grips the ssDNA (yellow) in place for nucleotide (orange) incorporation (PDB ID 4I27). (**d**) AlphaFold model showing corresponding Phe/Leu residue pair in AfPolX1 (salmon). Primer ssDNA, dideoxy TTP, and divalent magnesium shown for AfPolX1 are derived from 4I27 structure for MmTdT by superposition of polymerase Cα chains from 4I27 and AfPolX1 predicted structure. (**e**) Weblogo depicting the consensus within the region of loop1 (purple) entering into the β-strand of palm (pink) for sequences within the Polµ/TdT clade of phylogenetic tree in Fig. 1a, i.e. Leu, Phe, and Trp residues are depicted in blue and everything else in grey. (**f**) Gels depicting the template-independent activity of AfPolX1 in incorporating single nucleotides to a 10-base primer at 0, 0.5, 1, and 5 min time points. Schematics for the 10mer sequences used (substrates S5, S9, S10) are depicted in cartoon format above the associated gel, with the two 3′-end nucleotides emphasised. Substrate S10 is identical in sequence to S9, with the two 3′-end nucleotides changed to CA (depicted in green).

Wanting to establish AfPolX1’s primer length requirement to perform creative synthesis, we systematically shortened the primer ssDNA from a 21mer down to 10mer in steps of three or two nucleotides from its 3′ end. When probed with each of the four natural dNTPs individually, AfPolX1 chooses a different nucleotide to incorporate in case of each primer length (Fig. 4a and b, rows 1–5) without any identifiable pattern. TdT preferentially adds dGTP to ssDNA ends [30, 63] but has also been shown to rely on sequence context—the outcome being determined specifically by the last two nucleotide positions (P_−1_ and P_−2_) of the 3′ end of the primer [28, 64].

In TdT ternary structures (PDB ID:4I27), the hydrophobic interaction between the loop1 leucine 398 and the palm domain phenylalanine 405 has been observed to create a wedge that disrupts the stacking interaction between the 3′ base and the base immediately 5′ to it, separating the 3′ nucleotide of the ssDNA primer from the rest of the primer bases (Fig. 4c) [18]. In Polµ structures bound to either a gapped or a DSB substrate [56, 65], the lariat loop becomes displaced by the template strand, removing this loop1–palm interaction. TdT-like acceptance of ssDNA substrates has therefore been correlated to this Leu–Phe interaction. Identical locations in AfPolX1 appear to have these amino acid residues swapped (Leu→Phe; Phe→Leu), suggesting AfPolX1 could retain the hydrophobic interaction between Phe 562 (lariat loop) and Leu 569 (palm) (Fig. 4d). Indeed, the palm leucine is strongly conserved among the Polµ/TdT branch of fungal PolXs (Fig. 4e) and the complementary position within the lariat loop also remains a hydrophobic residue (F/W/M), more often being aromatic. Stabilising this interaction between the lariat loop and palm of PolXs for gripping the ssDNA appears to have originated earlier than the active site histidine and could serve to predict which X-family Pols have greater potential for utilising ssDNA substrates.

Hypothesising that the template-independent nucleotide incorporations observed by us for AfPolX1 were similarly guided by interactions of this PolX with the ssDNA primer end, and likely linked to the sequence context at the 3′, we shortened the 21mer primer while keeping the 3′OH sequence unchanged as -AC (Fig. 4a and b, rows 5–9). Both AfPolX1 WT and G501H incorporated only pyrimidines irrespective of the primer length, provided the 3′ end of the primer had -AC. AfPolX1 showed a slight preference for adding dCTP, while G501H generally added dTTP and dCTP equally well (comparing 0.5 min to 5 min). In our experiments, base selection appeared effectively decoupled from the nucleotide binding and addition process. Choice of base was determined by primer sequence rather than an intrinsic preference for a nucleotide. For instance, G501H added dGTP alone to one 10mer (Fig. 4f, top panel) while preferentially adding dCTP to others (Fig. 4f, middle and bottom panels). We also find that swapping just the two last bases of a 10mer primer from -AC to -CA (Fig. 4f, middle and bottom panels) reduced the overall use of this substrate by G501H, and contrary to our expectation that dGTP would be incorporated in this context, dCTP was primarily added. These observations implicate additional determinants that remain unresolved in our understanding of a terminal transferase’s catalytic mechanism.

### AfPolX1 incorporates modified nucleotides

**Figure 5.**
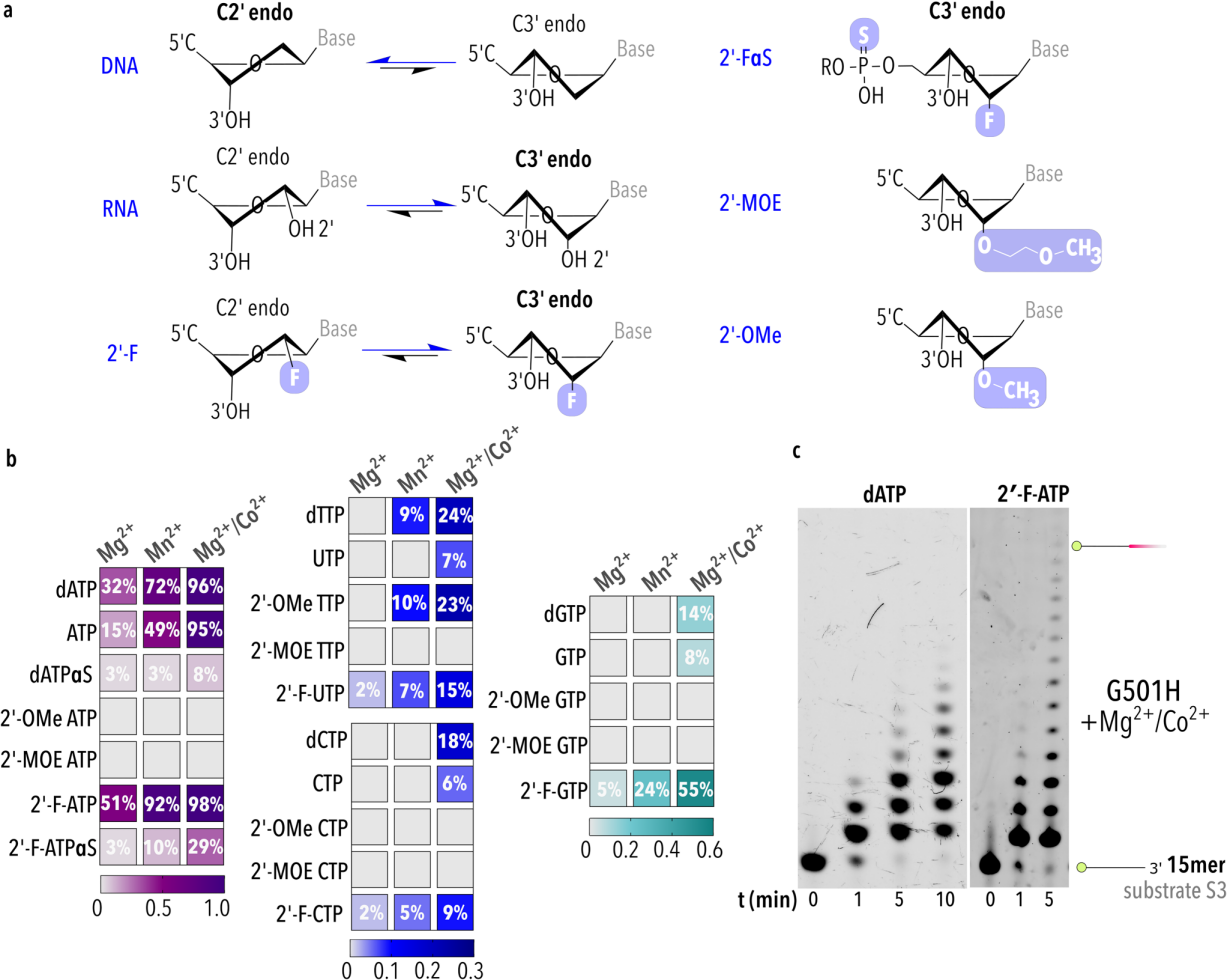
Template-independent incorporation of modified nucleotides by G501H. (**a**) Configuration of (deoxy)ribose sugar depending on the substituent [-H, -OH, -F, *O*-methoxyethyl (MOE), and -OMe] at the 2′ position of the incoming nucleotides is shown. Substituent groups are shaded in mauve. (**b**) Various modified nucleotides were incorporated into a 15mer ssDNA by G501H using different divalent ion conditions (10 mM Mg^2+^, 10 mM Mn^2+^, or 10 mM Mg^2+^/0.25 mM Co^2+^). Scale bars represent fraction extension. Mean percentage extension with Mg^2+^and Co^2+^ for all nucleotides showing extension >0 is shown within the plot. Data shown are from three independent replicates. (**c**) Gels demonstrating the differential terminal transferase activity on the 15mer (-GT) substrate (substrate S3, Supplementary Table S2) by G501H with dATP and 2′-F-ATP in the presence of 10 mM Mg^2+^/0.25 mM Co^2+^.

The considerably open, rather static, conformation of the TdT active site intrinsically makes substrate selection by TdT flexible. Various TdTs have been evolved to accept base, sugar, and backbone modifications, including fluorophore- and biotin-labelled dNTPs, locked nucleic acids (LNAs) and xeno nucleic acids (XNAs) [30, 66–68]. Given that AfPolX1 performs template-independent additions, we tested its inherent capacity to incorporate modified nucleotides on the short 15mer primer (Supplementary Table S2, substrate S3). Only the G501H mutant was evaluated because its terminal transferase activity was nearly twice that of WT on this primer sequence, to which AfPolX1 only adds dATP. Five different structural modifications routinely found in oligonucleotide therapeutics were assessed in comparison to unmodified dNTPs and rNTPs (Fig. 5).

Modifications at the 2′-hydroxyl group of ribose are extensively used in oligonucleotide therapeutics as they provide nuclease resistance and stability benefits without perturbing nucleic acid structure and pharmacological properties [69, 70]. We tested three types of modifications at this position—fluoro (F), OMe, and MOE, which result in the C3′ endo sugar conformation found in rNTPs (Fig. 5a). AfPolX1 showed exceptional acceptance of 2′-fluorinated substrates compared to any other modifications tested (Fig. 5b and Supplementary Figs S5 and S6). In every divalent condition tested (Mg^2+^, Mn^2+^, Mg^2+^/Co^2+^), 2′-F-ATP incorporation occurred efficiently (Fig. 5b) and formed the distinct ladder pattern (Fig. 5c), as seen for TdTs (Supplementary Fig. S5) [30]. The improvement in nucleotide incorporation becomes most obvious with Mg^2+^, where untemplated 2′-F-ATP addition resulted in nearly twice the product yielded from dATP addition (51% versus 32%, respectively) (Fig. 5b). The faster primer conversion is accompanied by longer product formation for this modification (comparing 5 min time points in Fig. 5c) and implies that AfPolX1 can also accept short fluorine containing oligonucleotides as substrates. We also compared dATP alpha-phosphorothioate (dATPαS) with its 2′-fluorinated counterpart (2′F-dATPαS) (Fig. 5b). In these modified nucleotides, a non-bridging oxygen on the alpha-phosphate is replaced with sulphur. For both nucleotides, incorporation is slower than dATP and is fastest when Co^2+^ is present. We find that introducing the fluorine group at the 2′ position enhances product synthesis, generating nearly 29% product with 2′-F-dATPαS compared to 8% with dATPαS. Overall, except 2′-F-ATP and 2′-F-GTP, incorporation of the modified nucleotides could not be observed with Mg^2+^ or Mn^2+^ alone, and were only detected when Mg^2+^ was supplemented with Co^2+^.

With Mg^2+^, G501H showed a clear bias for dATP incorporation on the 15mer substrate S3 (Fig. 4a and b, row3). This predisposition is also evident in its sparse incorporation of 2′-F-UTP (∼2%) compared to its ATP counterpart (51%), despite both nucleotides possessing an identical 2′ modification. Addition of a transition metal such as Mn^2+^ or Co^2+^ skews the base selection mechanism in-built within AfPolX1’s terminal transferase function in favour of other bases. Consistent with this, we observe variability in 2′-OMe incorporation, where only thymidine is accepted from among the four 2′-OMe nucleotides when Mn^2+^ or Co^2+^ is present (Fig. 5b). However, no incorporation could be obtained for any of the 2′-MOE modified nucleotides tested, irrespective of divalent(s) provided (Fig. 5b and Supplementary Fig. S6). This 2′ modification is sterically challenging for the TdT active site in general; calf thymus TdT incorporates 2′-MOE NTPs significantly slower when compared to other modified NTPs (Supplementary Figs S5 and S6).

Taken together, we find that while the GW motif makes PolXs more accepting of 2′-sugar substituents, the evolutionary divergence of these active sites, from fungi to mammals, has resulted in TdTs increased tolerance for a bulky, hydrophobic substituent at the 2′-position of the nucleotide sugar (Fig. 5a). Future studies to evaluate the structural determinants that prevent these incorporations are needed to help enhance AfPolX1’s terminal transferase ability.

## Discussion

We have provided the first detailed characterization of a fungal X-family DNA polymerase harbouring terminal transferase activity. Phylogenetic analysis suggests that an ancestral bacterial PolX possessing a 3′-5′ exonuclease activity evolved and diversified into the multiple PolX variants found in organisms [34, 71, 72]. Bacterial PolXs continued to evolve, with certain bacterial PolXs losing their polymerisation capability while retaining their 3′ phosphatase function [73]—a property that is absent in all other PolXs studied to date. The fungal branch of X-family polymerases that diverged from the bacterial parent then evolved in two directions. One of these branches evolved into early eukaryotic PolX representatives on the path towards a terminal transferase [14, 34]. Since TdT represents a specialised evolutionary branch of Polµ-like polymerases, a progenitor to TdT can be expected to combine the functional characteristics of Polµ and TdT. Our study provides biochemical evidence to support the evolution of this activity, as shown for AfPolX1. Key to our observations is that AfPolX1 performs template-independent primer extensions at physiological concentrations of magnesium. We find AfPolX1 also bears structural features intrinsic to both TdT and Polµ, as would be expected to have emerged in the PolX scaffold of an early TdT homolog. The existence of an unstructured loop1 coupled with the conservation of the Leu–Phe motif for interaction between the palm and loop1 are evidence of the importance of the terminal transferase activity in eukaryotic cells. Neither of these features is necessary to perform gap-filling activities by PolXs. In fungi, NHEJ forms an essential and often primary mechanism of dsDNA break repair [74]; therefore, the emergence of terminal transferase activity was likely relevant to providing compatible ends for DSB repair.

Our observations indicate that like Polµ and TdT [12, 64], AfPolX1’s interaction with the damaged ssDNA overhang of a break and its sequence context could determine the nucleotides incorporated to repair the damage. Consequently, AfPolX1 might directly influence any products made during NHEJ, as well as the errors that are accumulated [75]. However, the contributors influencing nucleobase recognition and selectivity could not be fully resolved in the absence of structural understanding and require further characterisation.

Metazoan TdTs have been identified and harnessed for their specialised activities; however, this remains an active area of investigation. The past five decades have seen the development of a formidable number of modifications to the backbone, sugar, and base of a nucleotide—all compatible with the phosphoramidite based solid-phase oligonucleotide synthesis process [76–78]. Achieving the goal of a scalable, enzymatic process of oligonucleotide synthesis for therapeutics using a TdT-dependent process requires the development of a similar diversity within the capabilities of the terminal transferases being evolved. Critical to developing the requisite breadth of activities in a ‘TdT-toolbox’ is a deeper knowledge of the determinants for template independence and nucleotide acceptance. Our study provides the foundational work that opens up the previously unexplored group of fungal PolXs as promising candidates for further development. A more in-depth study of the structure of these PolXs is necessary to understand how they function as terminal transferases. This knowledge will be key to developing improved fungal TdTs that can be used to grow the capabilities needed for scalable biocatalytic oligonucleotides synthesis.

## Supplementary Material

gkaf1497_Supplemental_Files

## Data Availability

All data underlying this article are available in the article and its online supplementary material. Raw data for the enzymatic assays including uncropped gels are available upon request from the corresponding author.

## Notes

Present address: Department of Biochemistry, University of Wisconsin—Madison, WI 53706, United States

Present address: Manchester Institute of Biotechnology, School of Chemistry, University of Manchester, Manchester M1 7DN, United Kingdom

Present address: Manchester Institute of Biotechnology, School of Chemistry, University of Manchester, Manchester M1 7DN, United Kingdom
