# Supplementary material for: Aspergillus fumigatus PolX1 is an early ancestor of vertebrate terminal deoxynucleotidyl transferases
Source: Nucleic Acids Res. 2026 Jan 23;54(3):gkaf1497. doi: 10.1093/nar/gkaf1497 (PMC12828229; doi:10.1093/nar/gkaf1497)
Supplement: gkaf1497_Supplemental_Files [file gkaf1497_supplemental_files.zip › Supplementary Data Parveen Revised.pdf]

SUPPLEMENTARY INFORMATION

Aspergillus fumigatus **PolX1 is an early ancestor of vertebrate terminal deoxynucleotidyl transferases**

Najma Parveen[1,2], Sophia Steblina[1], Abhijit Behera[2,3], Caecilie M. Benckendorff[4,5], Gavin J. Miller[4,5], Katie E. Davis[6,7], Purba Mukherjee[1,2,*]

[1]Department of Chemistry, University of York, Heslington, YO10 5DD, United Kingdom
[2]Department of Biological Sciences, Indian Institute of Science Education and Research, Kolkata, Mohanpur 741246, India
[3]Present address: Department of Biochemistry, University of Wisconsin-Madison, WI 53706, United States
[4]School of Chemical & Physical Sciences and Centre for Glycoscience, Keele University, Staffordshire ST5 5BG, United Kingdom
[5]Present address: Manchester Institute of Biotechnology, School of Chemistry, University of Manchester, Manchester, M1 7DN, United Kingdom
[6]Department of Biology, University of York, Heslington, YO10 5DD, United Kingdom
[7]School of Biosciences, University of Sheffield, Sheffield S10 2TN, United Kingdom

[*] To whom correspondence should be addressed. Email: purba.mukherjee@york.ac.uk

SUPPLEMENTARY RESULTS

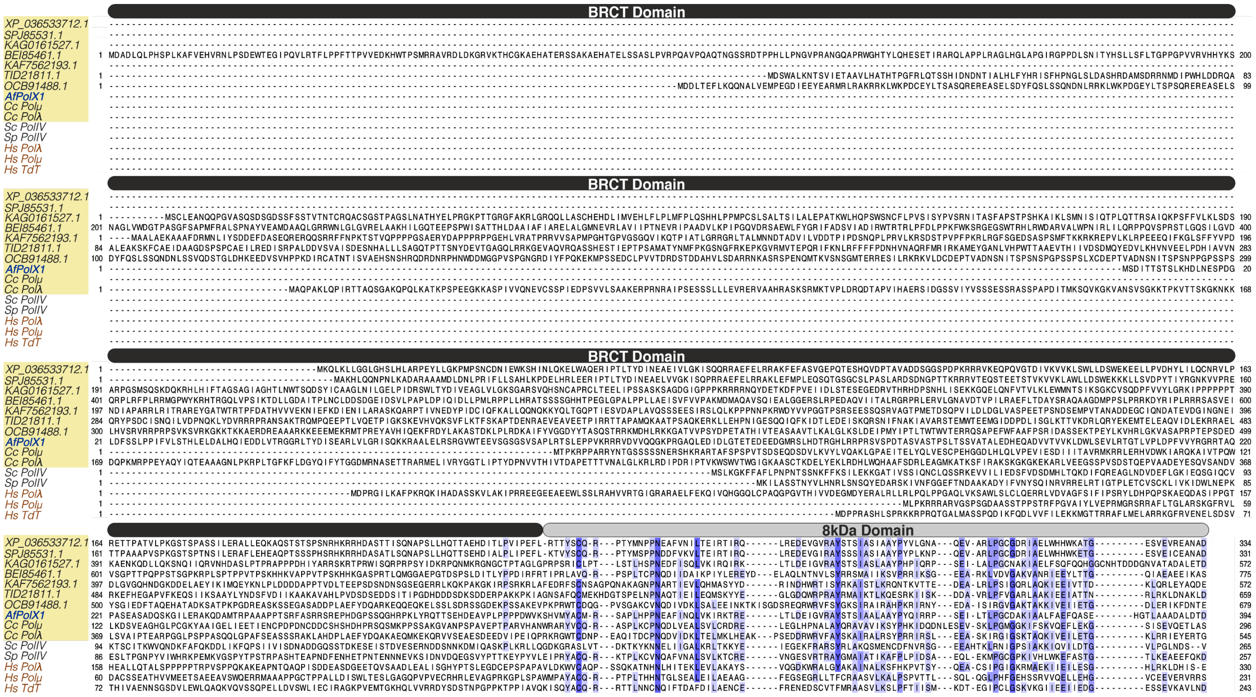

Figure S1: Multiple sequence alignment (MSA) of fungal (black) and human X-family polymerases (brown), depicting the various lengths of unaligned BRCT domains (black) and the aligned 8 kDa domains (gray). The fungal multicellular X family polymerases are highlighted with a box (yellow) with AfPolX1 shown in blue. The residues highlighted in different shades of blue depict the percentage conservation of amino acid in that position.

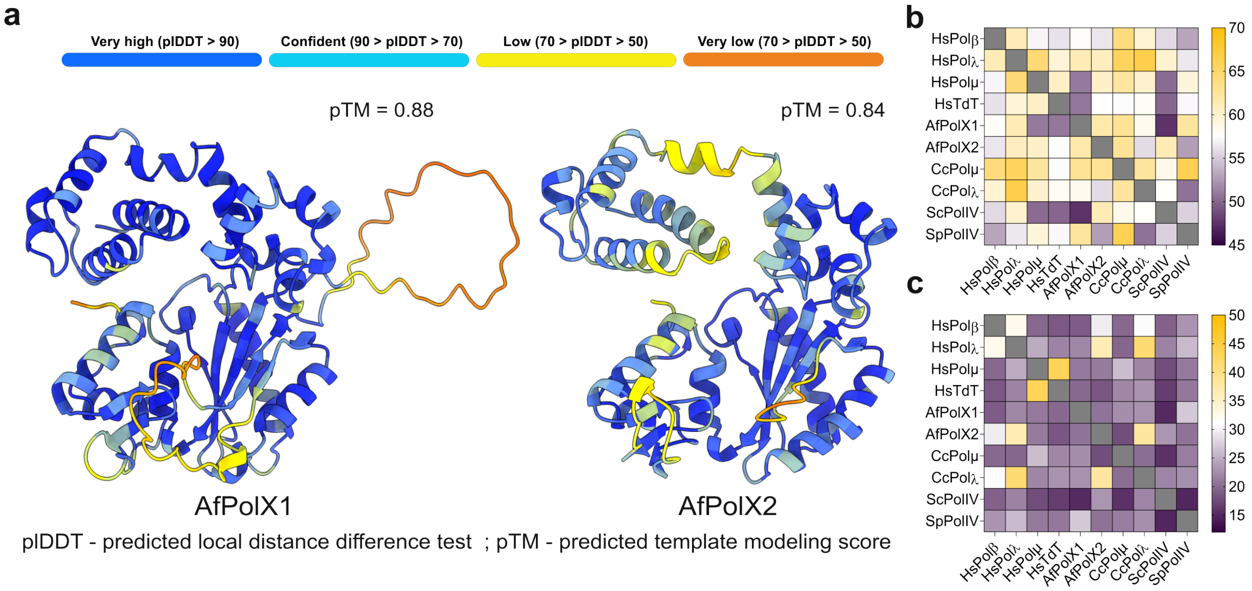

Figure S2: Structural and sequence analysis of AfPolXs. (a) AlphaFold3 predicted structures of the catalytic core of AfPolX1 and AfPolX2 coloured as per local confidence scores at the amino acid level (pIDDT values). (b) Similarity and (c) identity matrices comparing catalytic cores of AfPolX1 and AfPolX2 with human and other fungal X-family polymerases (Stothard, 2000).

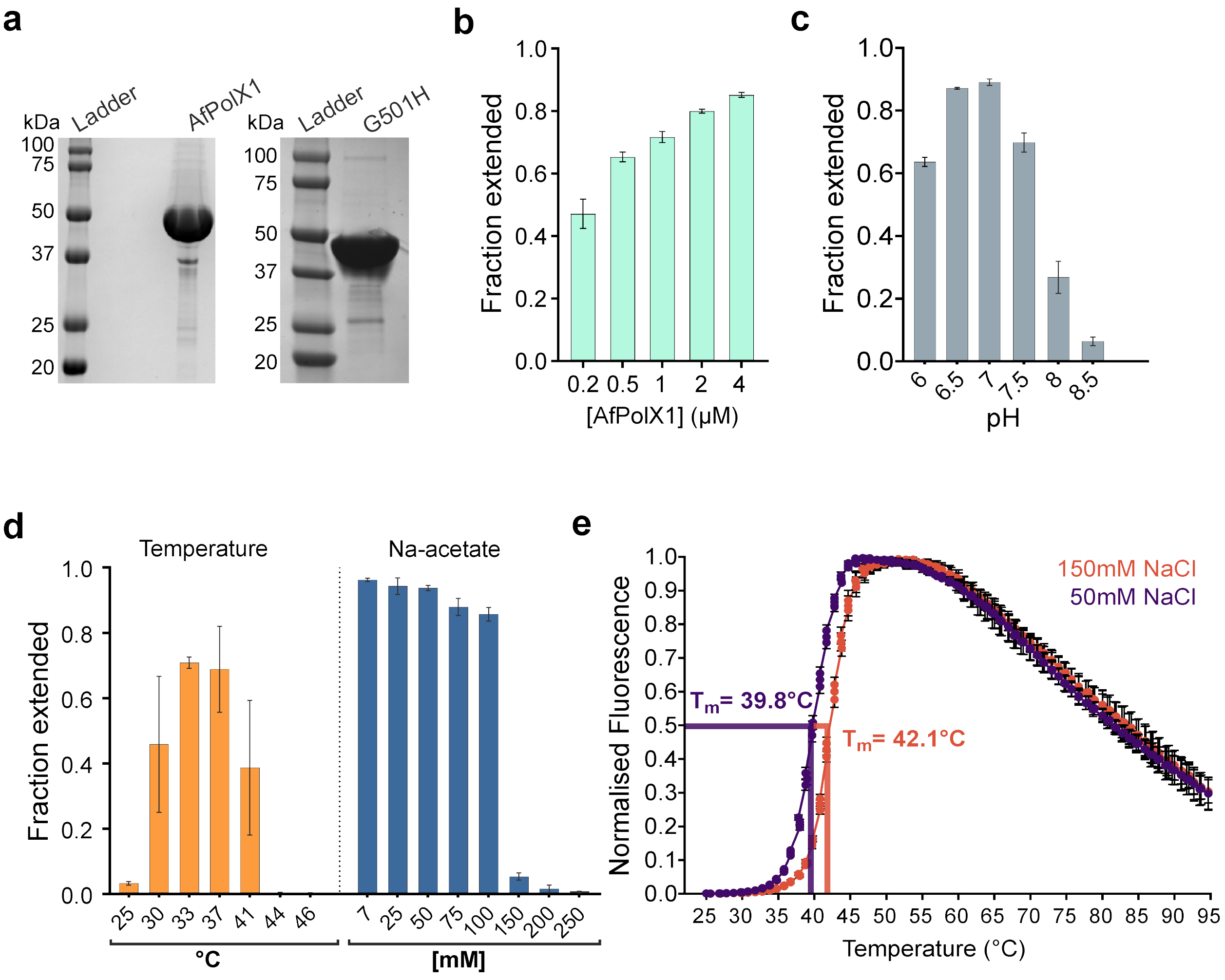

Figure S3: Characterisation of AfPolX1 (a) SDS-PAGE analysis of the purified AfPolX1WT and G501H mutant proteins. (b) Effect of varying AfPolX1 concentration (0.2 μM – 4 μM) in single nucleotide incorporation assays at 10 s, with substrate D3 and 1 mM dTTP in the presence of Mg^{2+}. (c) Effect of varying pH – MES (pH 6-6.5) or HEPES (pH 7-8.5) buffers – on primer extension of substrate D3 at 1 min. (d) Effects of temperature and Na^{+} ions on primer extension activity at 5 min, by AfPolX1 on substrate D4 with 1 mM of all four dNTPs and Mg^{2+} as divalent. (e) Comparison of thermal melt curves of AfPolX1 in the presence of 50 mM and 150 mM NaCl (data represents three independent replicates). Mean normalised fluorescence was plotted against temperature. Error bars represent standard deviation.

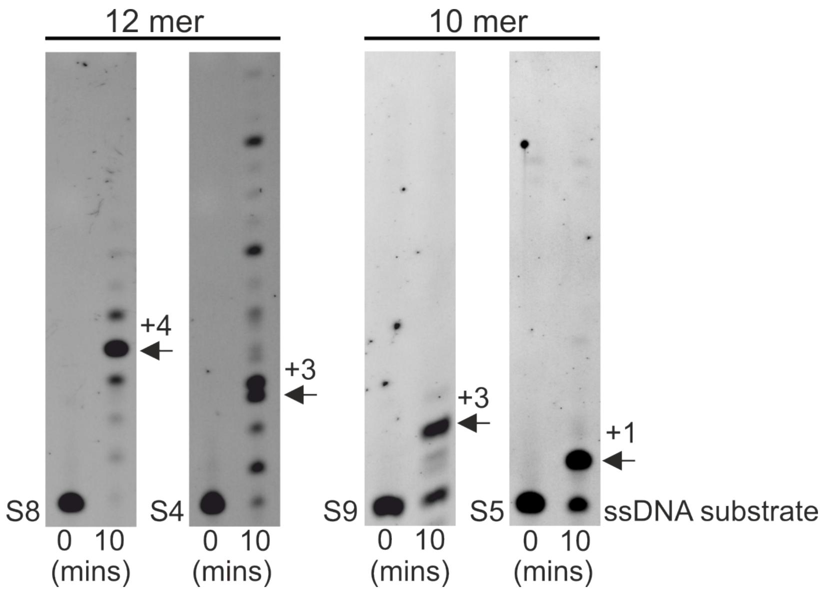

Figure S4: Gels comparing multiple nucleotide incorporations by 4 μM AfPolX1, on 25 nM 12mer and 10mer substrates with all four dNTPs (1 mM each), in the presence of 10 mM Mg^{2+}/0.25 mM Co^{2+} as divalent.

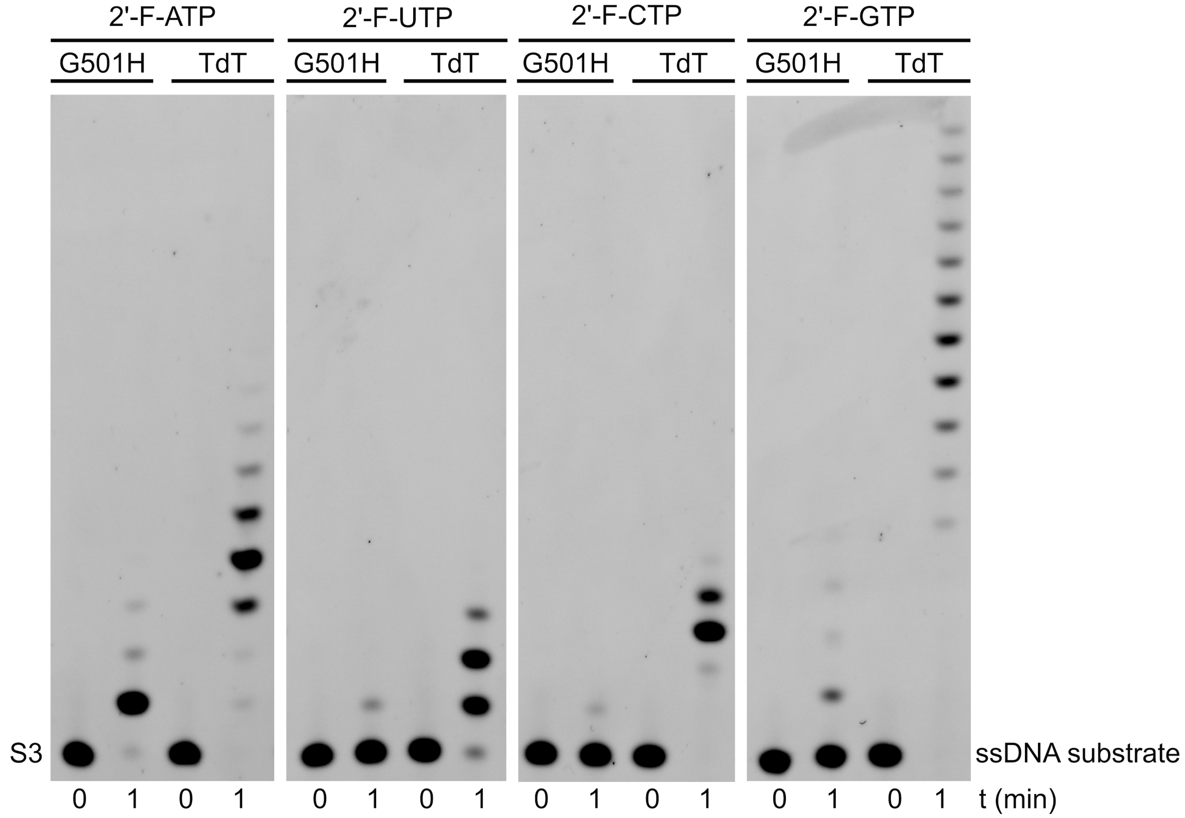

Figure S5: Gels comparing incorporations of various 2′-F NTPs (1 mM) by G501H (4 μM) or calf thymus TdT (4 μM) on 15mer substrate S3 (25 nM) in the presence of 10 mM Mg^{2+}/0.25 mM Co^{2+} as divalent.

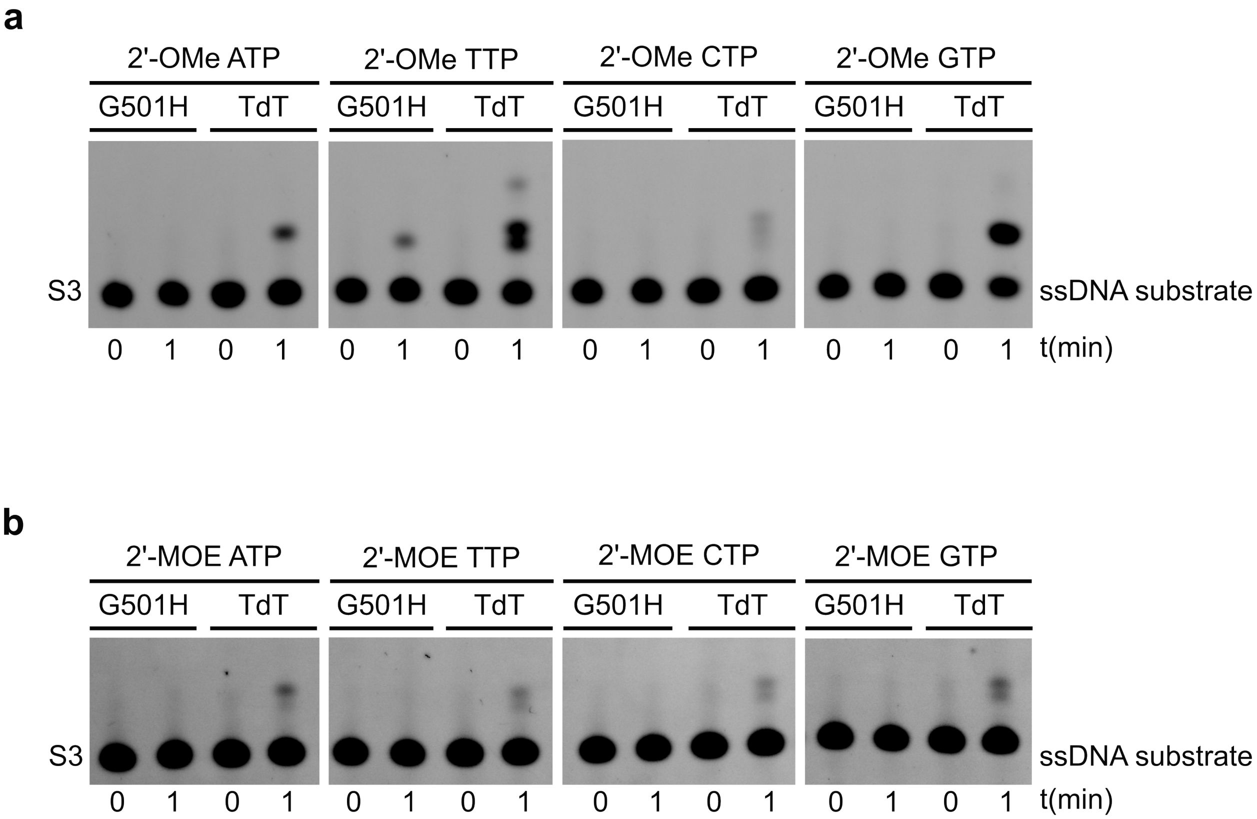

Figure S6: Gels comparing incorporations of various modified nucleotides, (a) 2′-OMe NTPs (1 mM) or (b) 2′-MOE NTPs (1 mM) by G501H (4 μM) or calf thymus TdT (4 μM) on 15mer substrate S3 (25 nM) in the presence of 10 mM Mg^{2+}/0.25 mM Co^{2+} as divalent.

SUPPLEMENTARY TABLES

Substrates	Sequence (5' – 3')
**D1**	/56-FAM/CTGTCTATCATAGCATTTAAGG ^{P}AGAGTCAGAGCATCTAACGCAGAGTCAG 3’<br>3’GACAGATAGTATCGTAAATTCC**X**TCTCAGTCTCGTAGATTGCGTCTCAGTC 5’<br><br>**X** = A, T, G, C
**D2**	/56-FAM/CTGTCTATCATAGCATTTAAGG AGAGTCAGAGCATCTAACGCAGAGTCAG 3’<br>3’GACAGATAGTATCGTAAATTCC**X**TCTCAGTCTCGTAGATTGCGTCTCAGTC 5’<br><br>**X** = A, T, G, C
**D3**	/56-FAM/CTGTCTATCATAGCATTTAAGG 3’<br>3’GACAGATAGTATCGTAAATTCCATCTCAGTC 5’
**D4**	/56-FAM/CTGTCTATCATAGCATTTAAGG 3’<br>3’GACAGATAGTATCGTAAATTCCTTCTCAGTCTCGTAGATTGCGTCTCAGTC 5’
**D5 (Trap)**	5’ CGCGAAATTAATACGACTCACTATAGGGGAATTGTGAGCGGATAACAATTCCCC 3’<br>3’ GCGCTTTAATTATGCTGAGTGATATCCCCTTAACACTCGCCTATTGTTAAGGGG 5’

Table S1: List of the dsDNA sequences used for biochemical characterisation of AfPolX1.

Substrates	Sequence (5’ – 3’)
**S1**	/56-FAM/ CTGTCTATCATAGGTATTAAC
**S2**	/56-FAM/ CTGTCTATCATAGGTATT
**S3**	/56-FAM/ CTGTCTATCATAGGT
**S4**	/56-FAM/ CTGTCTATCATA
**S5**	/56-FAM/ CTGTCTATCA
**S6**	/56-FAM/ TCTATCATAGGTATTAAC
**S7**	/56-FAM/ ATCATAGGTATTAAC
**S8**	/56-FAM/ ATAGGTAATAAC
**S9**	/56-FAM/ AGGTATTAAC
**S10**	/56-FAM/ AGGTATTACA

Table S2: List of ssDNA sequences used for assays.

SUPPLEMENTARY METHODS

Template-dependent nucleotide incorporation assays

Template-dependent nucleotide incorporation assays were carried out using AfPolX1 on primer-template DNA substrates, with 1 mM dTTP or 1 mM of all four dNTPs in the presence of 10 mM Mg^{2+} as divalent. At desired time points, reactions were quenched with an equal volume of quenching buffer comprising 90% formamide, 50 mM EDTA and 0.025% SDS. Samples were heated to 98°C for 10 minutes and loaded on a 15% acrylamide (19:1)-1X TBE denaturing gel containing 6 M urea to separate extension products from the unextended primer. The gels were imaged on an Amersham Typhoon 5 (Cytiva, USA) laser-based scanner using blue light (excitation wavelength = 495 nm; emission wavelength = 520 nm) allowing detection of the FAM labelled primers.

AfPolX1 concentration

Assay condition was varied to test for optimal AfPolX1 concentration (0.2 µM – 4 µM) in a single nucleotide incorporation assay with 25 nM substrate D3 and 1 mM dTTP. All reactions were performed at 37°C in buffer containing 25 mM HEPES (pH 7.5), 50 mM sodium acetate (NaOAc) and 10 mM Mg^{2+}.

pH, temperature and Na^+

Multiple nucleotide incorporation assays with 1 µM AfPolX1, varying reaction pH were performed in either 25mM MES (pH 6-6.5) or HEPES (pH 7-8.5) buffer and containing 50 mM NaOAc, 200 nM substrate D3 and 1 mM dNTP each. Multiple nucleotide incorporation assays varying temperature (25-46°C) and Na^+ concentration (7-250 mM) were carried out with 1 µM AfPolX1 on 200 nM substrate D4 in 50 mM HEPES (pH 7.5) buffer.

Synthesis of nucleoside triphosphates

General Experimental

All chemicals were purchased from Sapala Organics, Biosynth, Fisher Scientific or Sigma Aldrich and were used without further purification unless otherwise stated. The use of NH_4OH refers to NH_4OH (35% w/v). Anhydrous MeCN was obtained by drying over 3 Å molecular sieves and

stored in a J Young® flask. All reactions were conducted using anhydrous solvents, under an atmosphere of N_2, unless otherwise stated. Flash column chromatography was performed using silica gel, high purity grade, pore size 60 Å, 230-400 mesh particle size, 40-63 µm particle size (Sigma Aldrich). Thin layer chromatography (TLC) was performed using pre-coated 0.25 mm 60 F254 silica gel plates (Merck). Visualisation was achieved using UV light (λ = 254 nm). All high-resolution mass spectra were measured at the EPSRC National Mass Spectrometry Facility at Swansea University, UK. NMR spectra were recorded on a Bruker Avance 400 spectrometer. The chemical shift data for ^{1}H and ^{13}C signals are given as δ in units of parts per million (ppm) relative to tetramethylsilane, where δ = 0.00 ppm. The number of protons (n) for a given resonance is indicated by nH. The multiplicity of each signal is indicated by: s (singlet), br s (broad singlet), d (doublet), t (triplet), q (quartet), dd (doublet of doublets), ddd (doublet of doublet of doublets), dt (doublet of triplets), app. (apparent), m (multiplet) or ov. (overlapping). Coupling constants (*J*) are quoted in Hz and calculated to the nearest 0.1 Hz.

Analytical SAX HPLC

Mono- and triphosphorylation of nucleoside analogues was monitored *via* analytical HPLC.
Column: Agilent PL-SAX 8µm 1000 Å, 150 x 4.6 mm
Flow rate: 0.5 mL/min

Time (min.)	%A (0.01M KH_2PO_4, pH 2.6)	%B (0.5M KH_2PO_4, pH 3.5)
0.0	100	0
15.0	0	100
20.0	0	100
23.0	100	0
28.0	100	0

Retention times: Uncharged molecules (~4 min); monophosphates (~8 min); diphosphates (~12 min); triphosphates (~16 min).

Preparation of bis(tributylammonium)pyrophosphate

$Na_4P_2O_7$ (1.0 equiv.) was dissolved in H_2O and loaded onto a H^+ resin column (DOWEX 50W 8X, H^+ form). The column was eluted with H_2O directly into a round-bottom flask containing Bu_3N (1.0 M in EtOH, 2.0 equiv.) at 0°C, until the eluent was pH ~7. The mixture was concentrated *in vacuo*, and the residue was transferred to a Falcon™ tube and lyophilised prior to use.

2′-Deoxyadenosine 5′-*O*-[α-thio]triphosphate (dATPαS)

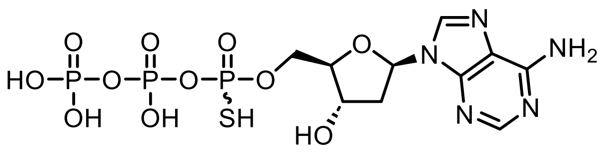

dATPαS (89.0 mg, 0.15 mmol, 7.5%) was synthesised following the general procedure for the synthesis of 5'-*O*-[α-thio]triphosphates from 2′-deoxyadenosine (500 mg, 1.99 mmol, 1.0 equiv.). ^{1}H NMR (400 MHz, D_2O) δ 8.63 (1H, s, H_8), 8.57 (1H, s, H_8), 8.25 (1H, s, H_2), 6.53 (1H, ov. app. t, $^3J_{H1'-H2'a/H2'b}$ = 6.6 Hz, $H_{1'}$), 6.52 (1H, ov. app. t, $^3J_{H1'-H2'a/H2'b}$ = 6.7 Hz, $H_{1'}$), 4.91 – 4.82 (2H, m, $H_{4'}$), 4.36 – 4.27 (4H, m, $H_{3'}$, $H_{5'a}$), 4.29 – 4.15 (2H, m, $H_{5'b}$) 2.85 (2H, app. dt, $^2J_{H2a'-H2'b}$ = 14.0 Hz, $^3J_{H2a'-H1'}$ = 6.7 Hz, $H_{2'a}$), 2.60 (2H, app. dt, $^2J_{H2'b-H2'a}$ = 14.0 Hz, $^3J_{H2'b-H1'}$ = 5.2 Hz, $H_{2'b}$); ^{13}C NMR (101 MHz, D_2O): δ 155.3 (C_6), 152.6 (C_2), 148.4 (C_4), 140.1 (C_8), 140.0 (C_8), 118.43 (C_5), 118.40 (C_5), 85.7 (d, $^3J_{C4'-P\alpha}$ = 9.4 Hz, $C_{4'}$), 85.6 (d, $^3J_{C4'-P\alpha}$ = 9.3 Hz, $C_{4'}$), 83.7 ($C_{1'}$), 83.6 ($C_{1'}$), 71.0 ($C_{3'}$), 70.9 ($C_{3'}$), 65.7 – 65.5 (m, $C_{5'}$), 39.11 ($C_{2'}$), 39.09 ($C_{2'}$); ^{31}P NMR (162 MHz, D_2O): δ 42.6 (d, $^2J_{P\alpha-P\beta}$ = 27.5 Hz, P_α), 42.1 (d, $^2J_{P\alpha-P\beta}$ = 28.4 Hz, P_α), −6.7 (d, $^2J_{P\gamma-P\beta}$ = 20.7 Hz, P_γ), −23.3 – −23.7 (m, P_β), HRMS (NSI): *m*/*z* calculated for $C_{10}H_{12}N_5Na_3O_{11}P_3S$ [M−Na]$^-$ 571.9165, found 571.9171.

2′-O-Methyladenosine 5′-*O*-triphosphate (2′OMe ATP)

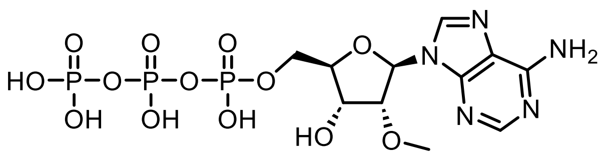

2′OMe ATP (318 mg, 0.54 mmol, 76%) was synthesised following general procedure for the synthesis of 5′-*O*-triphosphates from 2′-*O*-methyl adenosine (200 mg, 0.71 mmol). ^{1}H NMR (400 MHz, D_2O): δ 8.44 (1H, s, H_8), 8.15 (1H, s, H_2), 6.12 (1H, d, $^3J_{H1'-H2'}$ = 5.7 Hz, $H_{1'}$), 4.70 – 4.67 (1H, ov. dd, $^3J_{H3'-H2'}$ = 5.1 Hz, $^3J_{H3'-H4'}$ = 3.4 Hz, $H_{3'}$), 4.39 (1H, app. t, $^3J_{H2'-H1'/H3'}$ = 5.4 Hz, $H_{2'}$), 4.31 (1H, app. p, $^3J_{H4'-H3'/H5'a/H5'b}$ = 3.0 Hz, $^4J_{H4'-P\alpha}$ = 3.0 Hz, $H_{4'}$), 4.22 (1H, ddd, $^2J_{H5'a-H5'b}$ = 11.8 Hz, $^3J_{H5'a-P\alpha}$ = 6.0 Hz, $^3J_{H5'a-H4'}$ = 3.1 Hz, $H_{5'a}$), 4.15 (1H, ddd, $^2J_{H5'b-H5'a}$ = 11.7 Hz, $^3J_{H5'b-P\alpha}$ = 4.8 Hz, $^3J_{H5'b-H4'}$ = 3.1 Hz, $H_{5'b}$), 3.39 (3H, s, CH_3); ^{13}C NMR (101 MHz, D_2O): δ 155.6 (C_6), 153.0 (C_2), 149.0 (C_4), 139.9 (C_8), 118.6 (C_5), 85.3 ($C_{1'}$), 84.4 (d, $^3J_{C4'-P\alpha}$ = 9.1 Hz, $C_{4'}$), 83.1 ($C_{2'}$), 68.7 ($C_{3'}$), 65.0 (d, $^2J_{C5'-P\alpha}$ = 5.8 Hz, $C_{5'}$), 58.2 (OCH_3); ^{31}P NMR (162 MHz, D_2O): δ −7.2 (d, $^2J_{P\gamma-P\beta}$ = 17.9 Hz, P_γ), −11.1 (app. dtd, $^2J_{P\alpha-P\beta}$ = 18.8 Hz, $^3J_{P\alpha-H5'a/H5'b}$ = 6.1 Hz, $^4J_{P\alpha-H4'}$ = 2.4 Hz, P_α), −21.9 (app. t, $^2J_{P\beta-P\alpha/P\gamma}$ = 17.7 Hz, P_β); HRMS (NSI): *m*/*z* Calculated for $C_{11}H_{15}N_5O_{13}P_3Na_2$ [M−2Na+H]$^-$ 563.9680, found 563.9681.

2′-O-(Methoxyethyl)adenosine 5′-*O*-triphosphate (2′MOE ATP)

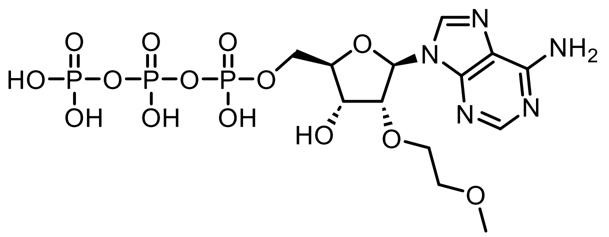

2′MOE ATP (360 mg, 0.56 mmol, 91%) was synthesised following general procedure for the synthesis of 5′-*O*-triphosphates from 2′-*O*-(2-methoxyethyl) adenosine (200 mg, 0.61 mmol). ^{1}H NMR (400 MHz, D_2O): δ 8.52 (1H, s, H_8), 8.24 (1H, s, H_2), 6.20 (1H, d, $^3J_{H1'-H2'}$ = 5.9 Hz, $H_{1'}$), 4.71 (1H, dd, $^3J_{H3'-H2'}$ = 5.2 Hz, $^3J_{H2'-H4'}$ = 3.4 Hz, $H_{3'}$), 4.60 (1H, app. t, $^3J_{H2'-H1'/H3'}$ = 5.6 Hz, $H_{2'}$), 4.47 – 4.39 (1H, m, $H_{4'}$), 4.35 – 4.17 (2H, m, $H_{5'a}$, $H_{5'b}$), 3.86 (1H, ddd, ^2J = 11.8 Hz, ^3J = 5.7 Hz, ^3J = 3.1 Hz, -O$CH_2$$CH_2OCH_3$), 3.74 (1H, ddd, ^2J = 11.8 Hz, ^3J = 5.5 Hz, ^3J = 3.3 Hz, -O$CH_2$$CH_2OCH_3$), 3.59 – 3.45 (2H, m, -$OCH_2$$CH_2$$OCH_3$), 3.17 (3H, s, -$OCH_2CH_2O$$CH_3$); ^{13}C NMR (101 MHz, D_2O): δ 155.6 (C_6), 152.8 (C_2), 149.0 (C_4), 140.0 (C_8), 118.5 (C_5), 85.5 ($C_{1'}$), 84.4 (d, $^3J_{C4'-P\alpha}$ = 9.2 Hz, $C_{4'}$), 82.2 ($C_{2'}$), 71.0 (-$OCH_2$$CH_2$$OCH_3$), 69.6 (-O$CH_2$$CH_2OCH_3$), 69.0 ($C_{3'}$), 65.1 (d, $^2J_{C5'-P\alpha}$ = 5.9 Hz, $C_{5'}$), 57.9 (-$OCH_2CH_2O$$CH_3$); ^{31}P NMR (162 MHz, D_2O): δ −7.03 (d, $^2J_{P\gamma-P\beta}$ = 19.6 Hz, P_γ), −11.0 (d, $^2J_{P\alpha-P\beta}$ = 18.9 Hz, P_α), −21.7 (app. t, $^2J_{P\beta-P\alpha/P\gamma}$ = 19.5 Hz, P_β); HRMS (NSI): *m*/*z* calculated for $C_{13}H_{18}N_5Na_3O_{14}P_3$ $[M-Na]^-$ 629.9762, found 629.9764.

2′-Deoxy-2′-fluoroadenosine 5′-*O*-triphosphate (2′F ATP)

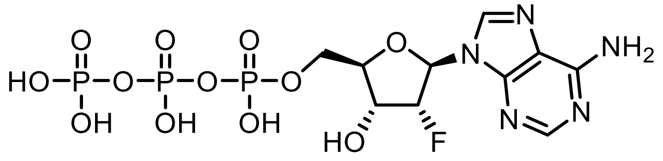

2′F ATP (360 mg, 0.61 mmol, 83%) was synthesised following the general procedure for the synthesis of 5′-*O*-triphosphates from 2′-deoxy-2′-fluoroadenosine (200 mg, 0.74 mmol); ^{1}H NMR (400 MHz, D_2O): δ 8.27 (1H, s, H_8), 7.99 (1H, s, H_2), 6.21 (1H, dd, $^3J_{H1'-F}$ = 16.6 Hz, $^3J_{H1'-H2'}$ = 2.0 Hz, $H_{1'}$), 5.27 (1H, ddd, $^2J_{H2'-F}$ = 52.1 Hz, $^3J_{H2'-H3'}$ = 4.4 Hz, $^3J_{H2'-H1'}$ = 1.9 Hz, $H_{2'}$), 4.66 (1H, ov. ddd, $^3J_{H3'-F}$ = 21.0 Hz, $^3J_{H3'-H4'}$ = 7.4 Hz, $^3J_{H3'-H2'}$ = 4.5 Hz, $H_{3'}$), 4.36 – 4.29 (2H, m, $H_{4'}$, $H_{5'a}$), 4.24 (1H, ddd, $^3J_{H5'b-H5'a}$ = 12.3 Hz, $^3J_{H5'b-P\alpha'}$ = 6.1 Hz, $^3J_{H5'b-H4'}$ = 3.3 Hz, $H_{5'b}$); ^{13}C NMR (101 MHz, D_2O): δ 155.1 (C_6), 152.4 (C_2), 147.8 (C_4), 139.5 (C_8), 118.31(C_3), 93.8 (d, $^1J_{C2'-F}$ = 187.2 Hz, $C_{2'}$), 86.1 (d, $^2J_{C1'-F}$ = 33.8 Hz, $C_{1'}$), 81.6 (dd, $^3J_{C4'-P\alpha}$ = 8.9 Hz, $^3J_{C4'-F}$ = 1.2 Hz, $C_{4'}$), 67.8 (d, $^2J_{C3'-F}$ = 15.8 Hz, $C_{3'}$), 63.7 (d, $^2J_{C5'-P\alpha}$ = 5.3 Hz, $C_{5'}$); ^{19}F NMR (377 MHz, D_2O): δ −203.9 (ddd, $^2J_{F-H2'}$ = 52.0 Hz, $^3J_{F-H3'}$ = 21.1 Hz, $^3J_{F-H1'}$ = 16.5 Hz); ^{31}P{^{1}H} NMR (162 MHz, D_2O): δ −8.0 (d, $^2J_{P\gamma-P\beta}$ = 18.9 Hz, P_γ), −11.0 (d, $^2J_{P\alpha-P\beta}$ = 18.9 Hz, P_β), -21.8 (app. t, $^2J_{P\beta-P\alpha/P\gamma}$ = 19.5 Hz, P_β); LRMS: (NSI) *m*/*z* calculated for $C_{10}H_{14}FN_5O_{12}P_3$ $[M-H]^-$ 508.0, found 507.9.

2′-Deoxy-2′-fluoroadenosine 5′-*O*-[α-thio]triphosphate (2′F ATPαS)

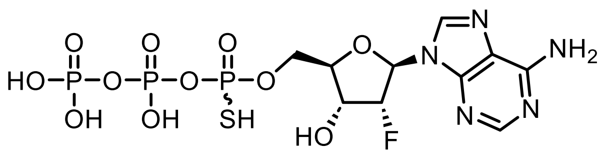

2′F ATPαS (161 mg, 0.20 mmol, 33%) was synthesised following the general procedure for the synthesis of 5’-*O*-[α-thio]triphosphates from 2′-deoxy-2′-fluoroadenosine (200 mg, 0.74 mmol); ^{1}H NMR (400 MHz, D_2O): δ 8.63 (1H, s, H_8), 8.53 (1H, s, H_8), 8.26 (2H, s, H_2), 6.44 (2H, dt, $^3J_{H1'-F}$ = 16.5 Hz, $^3J_{H1'-F}$ = 2.2 Hz, $H_{1'}$), 5.42 (1H, ddt, $^2J_{H2'-F}$ = 51.9 Hz, $^3J_{H2'-H3'}$ = 3.8 Hz, $^3J_{H2'-H3'}$ = 1.7 Hz, $H_{2'}$), 4.79 (ov. m, $H_{3'}$), 4.49 – 4.37 (6H, m, $H_{4'}$, $H_{5'a}$, $H_{5'b}$); ^{13}C NMR (101 MHz, D_2O) δ 171.0 (C=O, formate salt), 155.0 (C_6), 152.4 (C_2), 147.7 (C_4), 139.6 (C_8), 139.5 (C_8), 118.2 – 118.0 (m, C_5), 93.7 (d, $^1J_{C2'-F}$ = 187.2 Hz, $C_{2'}$), 93.7 (d, $^1J_{C2'-F}$ = 187.6 Hz, $C_{2'}$), 86.1 (d, $^2J_{C1'-F}$ = 32.9 Hz, $C_{1'}$), 86.1 (d, $^2J_{C1'-F}$ = 33.8 Hz, $C_{1'}$), 81.5 (d, $^3J_{C4'-P\alpha}$ = 9.3 Hz, $C_{4'}$), 81.3 (d, $^3J_{C4'-P\alpha}$ = 9.3 Hz, $C_{4'}$), 68.0 (d, $^2J_{C3'-F}$ = 15.4 Hz, $C_{3'}$), 67.9 (d, $^2J_{C3'-F}$ = 15.1 Hz, $C_{3'}$), 64.2 – 63.9 (m, $C_{5'}$); ^{31}P NMR (162 MHz, D_2O): δ 43.3 (d, $^2J_{P\alpha-P\beta}$ = 26.9 Hz, P_α), 43.0 (d, $^2J_{P\alpha-P\beta}$ = 28.0 Hz, P_α), −7.76 (d, $^2J_{P\gamma-P\beta}$ = 20.7 Hz, P_γ), −23.0 – −23.6 (m, P_β); LRMS (NSI): *m/z* calculated for $C_{10}H_{14}FN_5O_{12}P_3S$ [M−H]− 524.0, found 523.9.

2′-*O*-Methylthymidine 5′-*O*-triphosphate (2′OMe TTP)

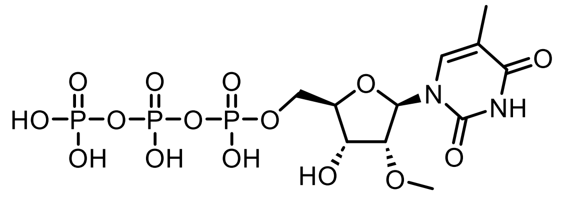

2′OMe TTP (122 mg, 0.30 mmol, 33%) was synthesised following general procedure for the synthesis of 5′-*O*-triphosphates from 2′-*O*-methyl thymidine (250 mg, 0.92 mmol). ^{1}H NMR (400 MHz, D_2O) δ 7.65 (1H, s, H_6), 5.91 (1H, d, $^3J_{H1'-H2'}$ = 4.7 Hz, $H_{1'}$), 4.43 (1H, app. t, $^3J_{H2'-H1'/H3'}$ = 4.7 Hz, $H_{2'}$), 4.21 – 4.08 (3H, m, $H_{4'}$, $H_{5'a}$, $H_{5'b}$), 3.98 (1H, app. t, $^3J_{H3'-H2'/H4'}$ = 5.1 Hz, $H_{3'}$), 3.38 (3H, s, OCH_3), 1.82 (3H, s, CH_3); ^{13}C NMR (101 MHz, D_2O): δ 166.6 (C_4, C=O), 151.7 (C_2, C=O), 134.0 (C_6), 111.9 (C_5), 86.8 ($C_{1'}$), 83.1 (d, $^3J_{C4'-P\alpha}$ = 8.4 Hz, $C_{4'}$), 82.4 ($C_{2'}$), 68.0 ($C_{3'}$), 64.9 (d, $^2J_{C5'-P\alpha}$ = 6.8 Hz, $C_{5'}$), 58.1 (OCH_3), 46.7 [$N(CH_2CH_3)_3$], 11.7 (CH_3), 8.2 [$N(CH_2CH_3)_3$]; ^{31}P{^{1}H} NMR (162 MHz, D_2O) δ −7.6 (br. s, P_α), −11.4 (d, $^2J_{P\gamma-P\beta}$ = 19.3 Hz, P_γ), −22.0 (br. s, P_β); LRMS: (NSI) *m/z* calculated for $C_{11}H_{18}N_2O_{15}P_3$ $[M-H]^-$ 511.0, found 511.0 .

2′-O-(Methoxyethyl)thymidine 5′-O-triphosphate (2′MOE TTP)

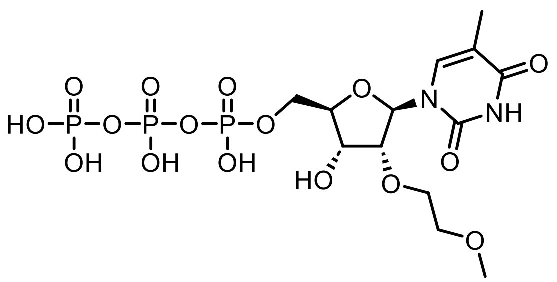

2′MOE TTP (297 mg, 0.46 mmol, 49%) was synthesised following general procedure for the synthesis of 5′-*O*-triphosphates from 2′-*O*-(2-methoxyethyl) thymidine (300 mg, 0.95 mmol). ^{1}H NMR (400 MHz, D_2O): δ 7.70 (1H, d, $^4J_{H6\text{-}CH3}$ = 1.4 Hz, H_6), 5.96 (1H, d, $^3J_{H1'\text{=}H2'}$ = 5.0 Hz, $H_{1'}$), 4.45 (1H, app. t, $^3J_{H3'\text{-}H2'/H4'}$ = 4.8 Hz, $H_{3'}$), 4.20 (3H, m, $H_{4'}$, $H_{5'a}$, $H_{5'b}$), 4.14 (1H, app. t, $^3J_{H2'\text{-}H1'/H3'}$ = 5.1 Hz, $H_{2'}$), 3.82 – 3.71 (2H, m, -OCH_2CH$_2$OCH$_3$), 3.58 – 3.49 (2H, m, -OCH$_2$CH_2OCH$_3$), 3.27 (3H, s, -OCH$_2$CH$_2$OCH_3), 1.86 (3H, d, $^4J_{CH3\text{-}H6}$ = 1.2 Hz, CH_3); ^{13}C NMR (101 MHz, D_2O): δ 166.5 (C_4, C=O), 151.8 (C_2, C=O), 137.1 (C_6), 111.8 (C_5), 86.8 ($C_{1'}$), 83.4 (d, $^3J_{C4'\text{-}P\alpha}$ = 9.3 Hz, $C_{4'}$), 81.3 ($C_{2'}$), 71.1 (-OCH$_2$*C*H$_2$OCH$_3$), 69.4 (-O*C*H$_2$CH$_2$OCH$_3$), 68.3 ($C_{3'}$), 64.8 (d, $^2J_{C5'\text{-}P\alpha}$ = 5.6 Hz, $C_{5'}$), 58.1 (-OCH$_2$CH$_2$O*C*H$_3$), 11.7 (CH_3); ^{31}P{^{1}H} NMR (162 MHz, D_2O): δ −8.40 (br. s, P_γ), −11.3 (d, $^2J_{P\alpha\text{-}P\beta}$ = 19.1 Hz, P_α), −22.1 (br. s, P_β); HRMS (NSI) *m*/*z* calculated for $C_{13}H_{22}N_2O_{16}P_3$ [M−H]$^-$ 555.0188, found 555.0192.

2′-Deoxy-2′-fluorouridine 5′-*O*-triphosphate (2′F UTP)

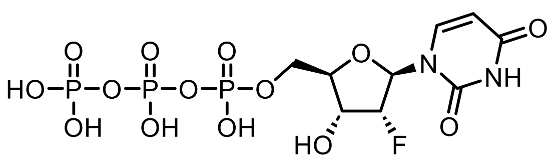

2′F UTP (182 mg, 0.31 mmol, 31%) was synthesised following the general procedure for the synthesis of 5′-*O*-triphosphates from 2′-deoxy-2′-fluorouridine (250 mg, 1.02 mmol). ^{1}H NMR (400 MHz, D_2O): δ 7.88 (1H, d, $^3J_{H6\text{-}H5}$ = 8.1 Hz, H_6), 6.01 (1H, dd, $^3J_{H1'\text{-}F}$ = 18.0 Hz, $^3J_{H1'\text{-}H2'}$ = 1.4 Hz, $H_{1'}$), 5.86 (1H, d, $^3J_{H5\text{-}H6}$ = 8.2 Hz, H_5), 5.11 (1H, ddd, $^2J_{H2'\text{-}F}$ = 52.4 Hz, $^3J_{H2'\text{-}H3'}$ = 4.6 Hz, $^3J_{H2'\text{-}H1'}$ = 1.4 Hz, $H_{2'}$), 4.50 (1H, ddd, $^3J_{H3'\text{-}F}$ = 23.1 Hz, $^3J_{H3'\text{-}H4'}$ = 8.2 Hz, $^3J_{H3'\text{-}H4'}$ = 4.5 Hz, $H_{3'}$), 4.35 – 4.08 (3H, m, $H_{4'}$, $H_{5'a}$, $H_{5'b}$); ^{13}C NMR (101 MHz, D_2O): δ 166.3 (C_4, C=O), 151.3 (C_2, C=O), 142.0 (C_6), 102.2 (C_5), 93.3 (d, $^1J_{C2'\text{-}F}$ = 185.9 Hz, $C_{2'}$), 88.7 (d, $^2J_{C1'\text{-}F}$ = 35.4 Hz, $C_{1'}$), 81.1 (dd, $^2J_{C5'\text{-}P\alpha}$ = 8.4 Hz, $^4J_{C5'\text{-}F}$ = 1.3 Hz, $C_{5'}$), 67.4 (d, $^2J_{C3'\text{-}F}$ = 16.1 Hz, $C_{3'}$), 63.9 – 63.6 (m, $C_{4'}$); ^{19}F NMR (377 MHz, D_2O): δ −202.6 (ddd, $^2J_{F\text{-}H2'}$ = 52.4 Hz, $^3J_{F\text{-}H3'}$ = 22.8 Hz, $^3J_{F\text{-}H1'}$ = 18.0 Hz); ^{31}P NMR (162 MHz, D_2O): δ −6.24 – −6.83 (m, P_γ), −11.2 (d, $^2J_{P\alpha\text{-}P\beta}$ = 19.6 Hz, P_α), −21.7 – −22.6 (m, P_β); HRMS (NSI): *m*/*z* calculated for $C_9H_{10}FN_2Na_3O_{14}P_3$ [M−Na]$^-$ 550.9027, found 550.9031.

2′-*O*-Methylguanosine 5′-*O*-triphosphate (2′OMe GTP)

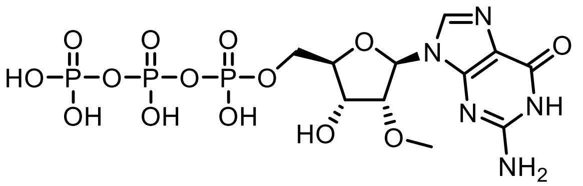

2′OMe GTP was synthesised following general procedure for the synthesis of 5′-*O*-triphosphates from 2′-*O*-Methyl guanosine. ^{1}H NMR (400 MHz, D_2O): δ 8.05 (1H, s, H_8), 5.93 (1H, d, $^{3}J_{H1'-H2'}$ = 5.7 Hz, $H_{1'}$), 4.69 – 4.60 (1H, m, $H_{3'}$), 4.39 (1H, app. t, $^{3}J_{H2'-H1'/H3'}$ = 5.4 Hz, $H_{2'}$), 4.28 – 4.27 (1H, m, $H_{4'}$), 4.19 – 4.17 (2H, m, $H_{5'a}$, $H_{5'b}$), 3.39 (3H, s, OCH_3); ^{13}C NMR (101 MHz, D_2O) δ 85.3 ($C_{1'}$), 83.9 (m, $C_{4'}$), 82.6 ($C_{2'}$), 68.7 ($C_{3'}$), 58.2 (m, $C_{5'}$), 48.9 (OCH_3); ^{31}P{^{1}H} NMR (162 MHz, D_2O): δ −5.66 (app. s, P_γ), −10.7 (d, $^{2}J_{P\alpha-P\beta}$ = 15.0 Hz, P_α), −19.2 (app. s, P_β); LRMS (NSI): *m*/*z* calculated for $C_{11}H_{17}N_5O_{14}P_3$ $[M-H]^-$ 536.0, found 535.8.

2′-*O*-Methylcytidine 5′-*O*-triphosphate (2′OMe CTP, Et_3NH^+ salt)

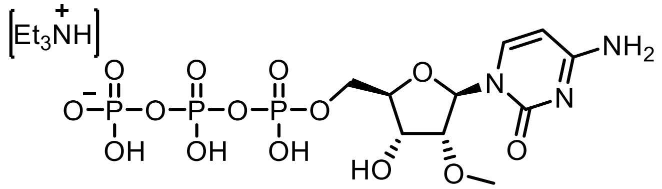

2′OMe CTP was synthesised following general procedure for the synthesis of 5′-*O*-triphosphates from 2′-*O*-methyl cytidine. ^{1}H NMR (400 MHz, D_2O) δ 8.06 (1H, d, $^{3}J_{H6-H5}$ = 7.7 Hz, H_6) 6.23 (1H, d, $^{3}J_{H5-H6}$ = 7.7 Hz, H_5), 6.05 (1H, d, $^{3}J_{H1'-H2'}$ = 3.6 Hz, $H_{1'}$), 4.48 (1H, t, $^{3}J_{H3'-H2'/H4'}$ = 5.5 Hz, $H_{3'}$), 4.38 – 4.26 (3H, m, $H_{4'}$, $H_{5'a}$, $H_{5'b}$), 4.05 (1H, dd, $^{3}J_{H2'-H3'}$ = 5.1 Hz, $^{3}J_{H2'-H1'}$ = 3.6 Hz, $H_{2'}$), 3.55 (3H, s, OCH_3); 3.21 (3H, q, *J* = 7.3 Hz, $[N(CH_2CH_3)_3]$), 1.29 (6H, t, *J* = 7.3 Hz, $[N(CH_2CH_3)_3]$); ^{13}C NMR (101 MHz, D_2O): δ 163.7 (C_4), 142.4 (C_6), 96.1 (C_5), 87.7 ($C_{1'}$), 83.1 ($C_{2'}$), 82.8 (m, $C_{4'}$), 67.7 ($C_{3'}$), 64.4 (m, $C_{5'}$), 58.2 (OCH_3), 46.7 $[N(CH_2CH_3)_3]$, 8.2 $[N(CH_2CH_3)_3]$; ^{31}P{^{1}H} NMR (162 MHz, D_2O): δ −9.07 (m, P_γ), −11.0 (m, P_α), −21.3 (m, P_β); LRMS (NSI): *m*/*z* calculated for $C_{10}H_{17}N_3O_{14}P_3$ $[M-H]^-$ 496.0, found 495.9.

dATPαS: ^{1}H NMR (400 MHz, D_2O)

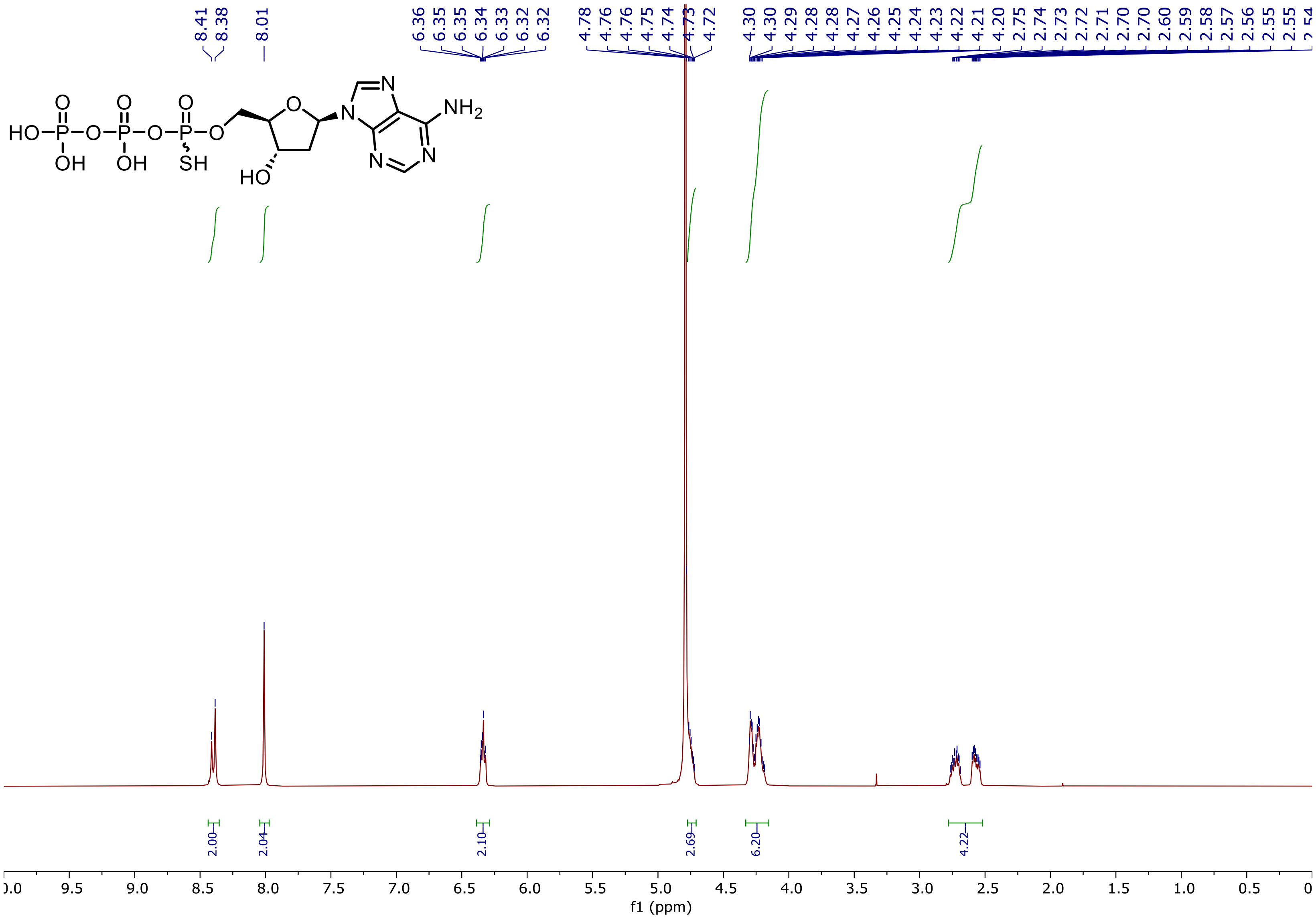

^{13}C NMR (101 MHz, D_2O)

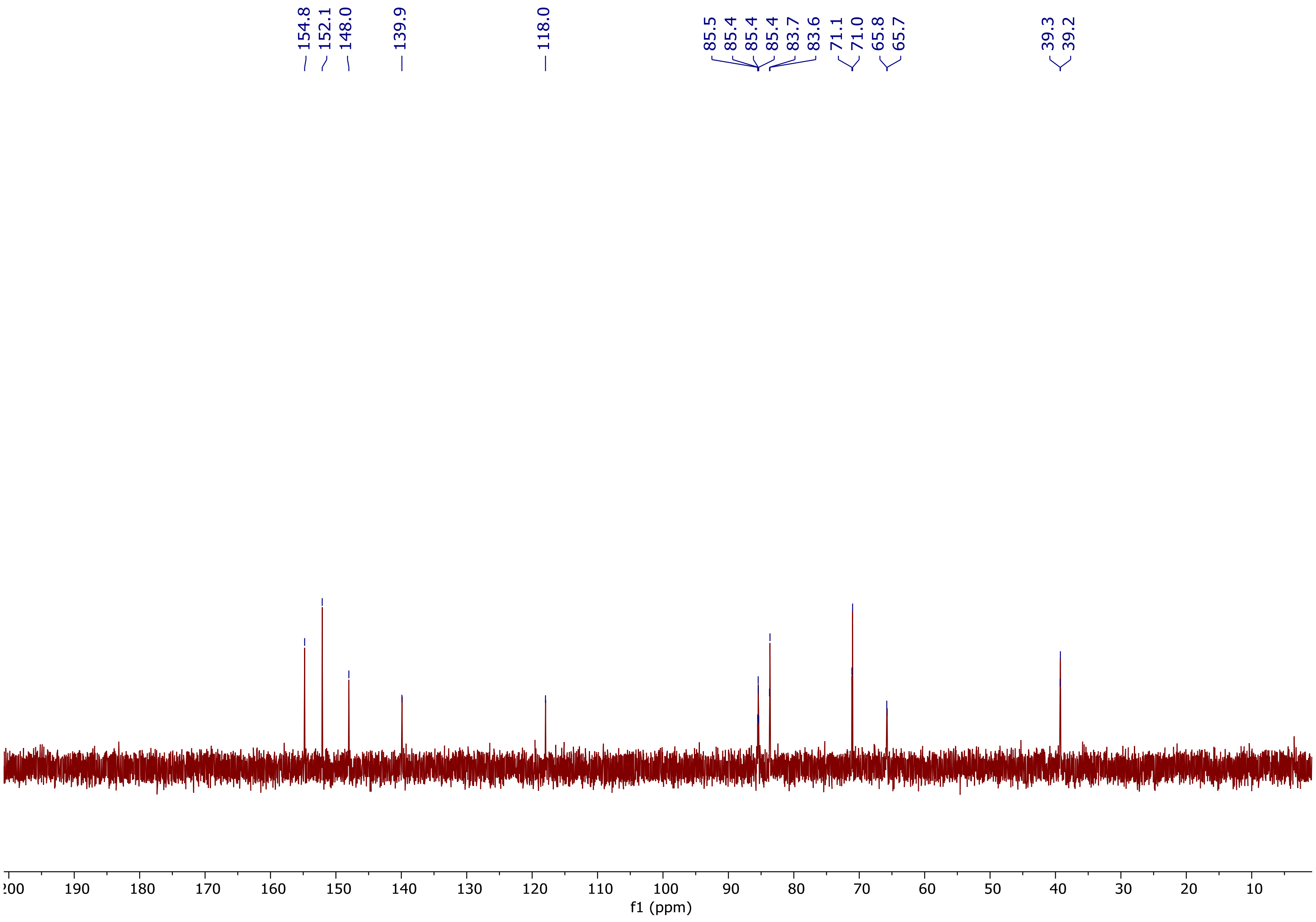

^{31}P NMR (162 MHz, D_2O)

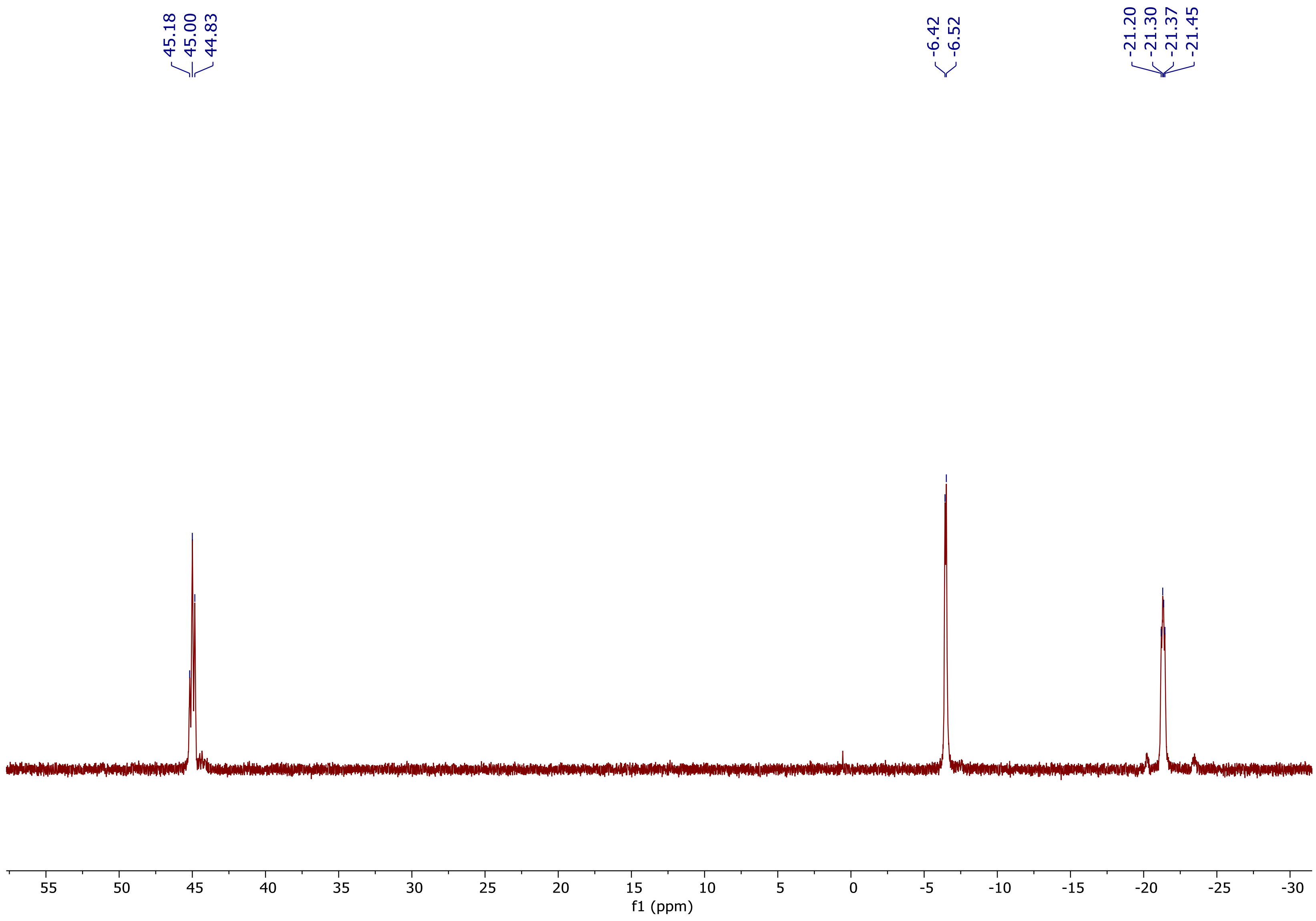

2′OMe ATP: ¹H NMR (400 MHz, D₂O)

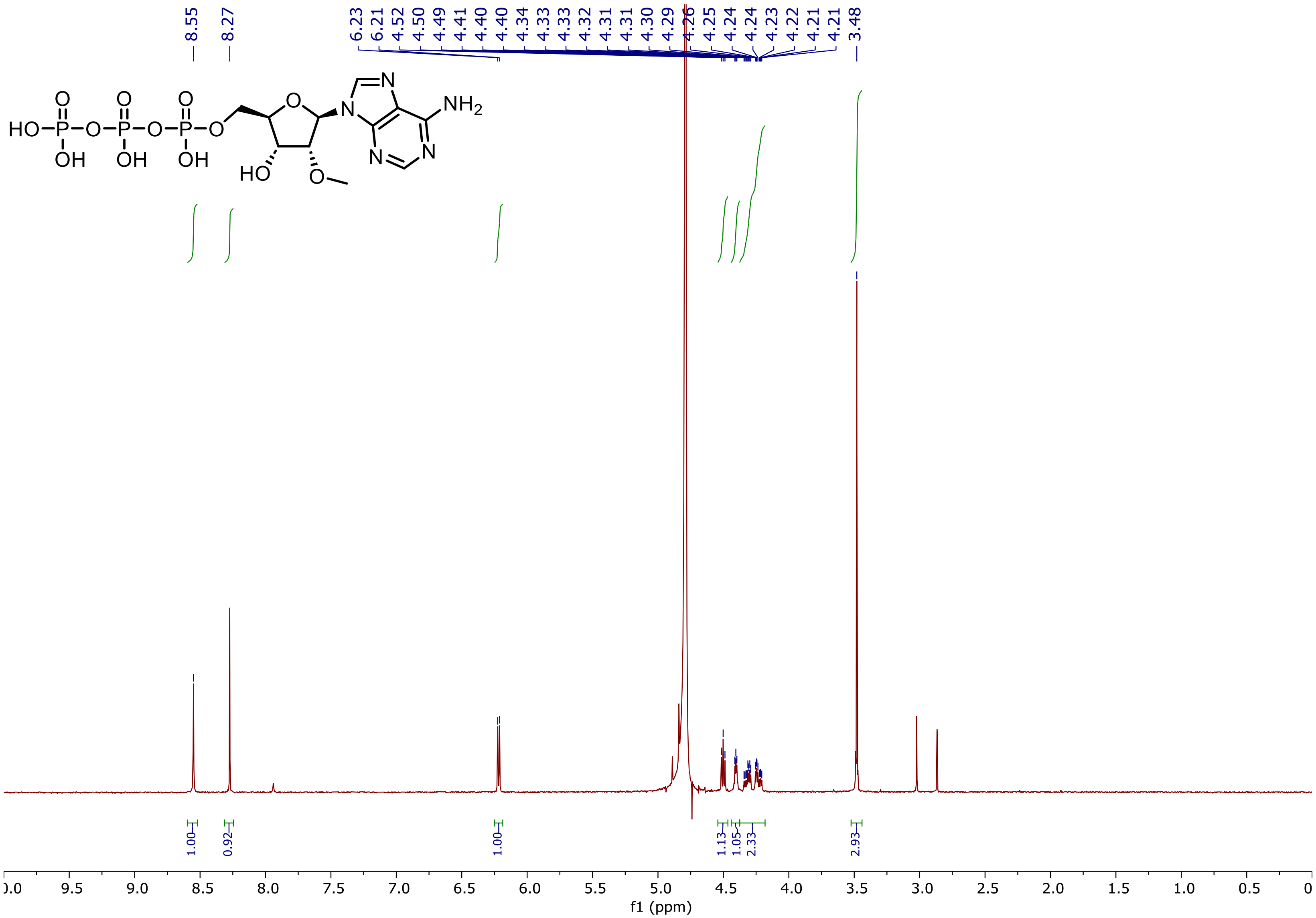

^{13}C NMR (101 MHz, D_2O)

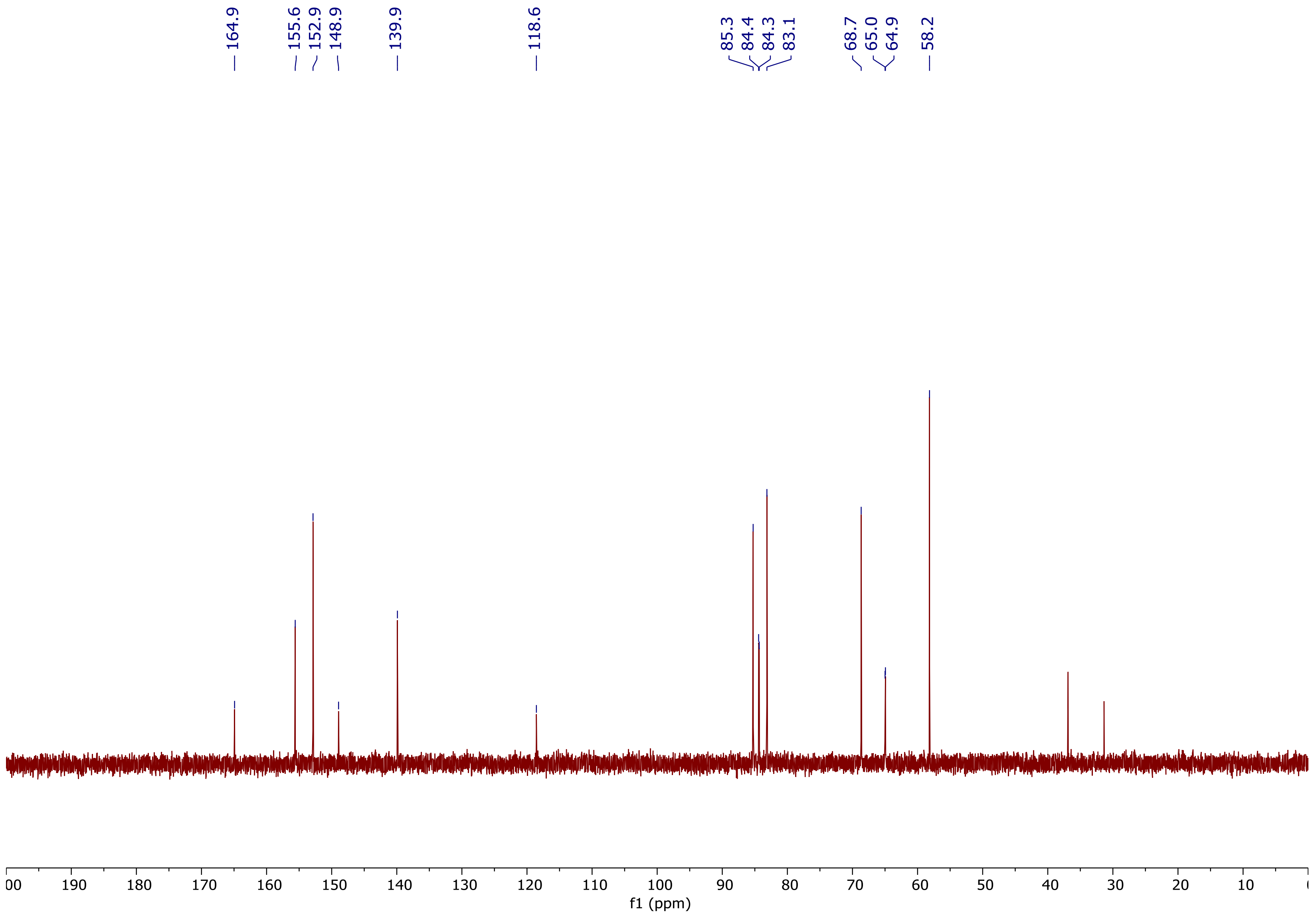

^{31}P NMR (162 MHz, D_2O)

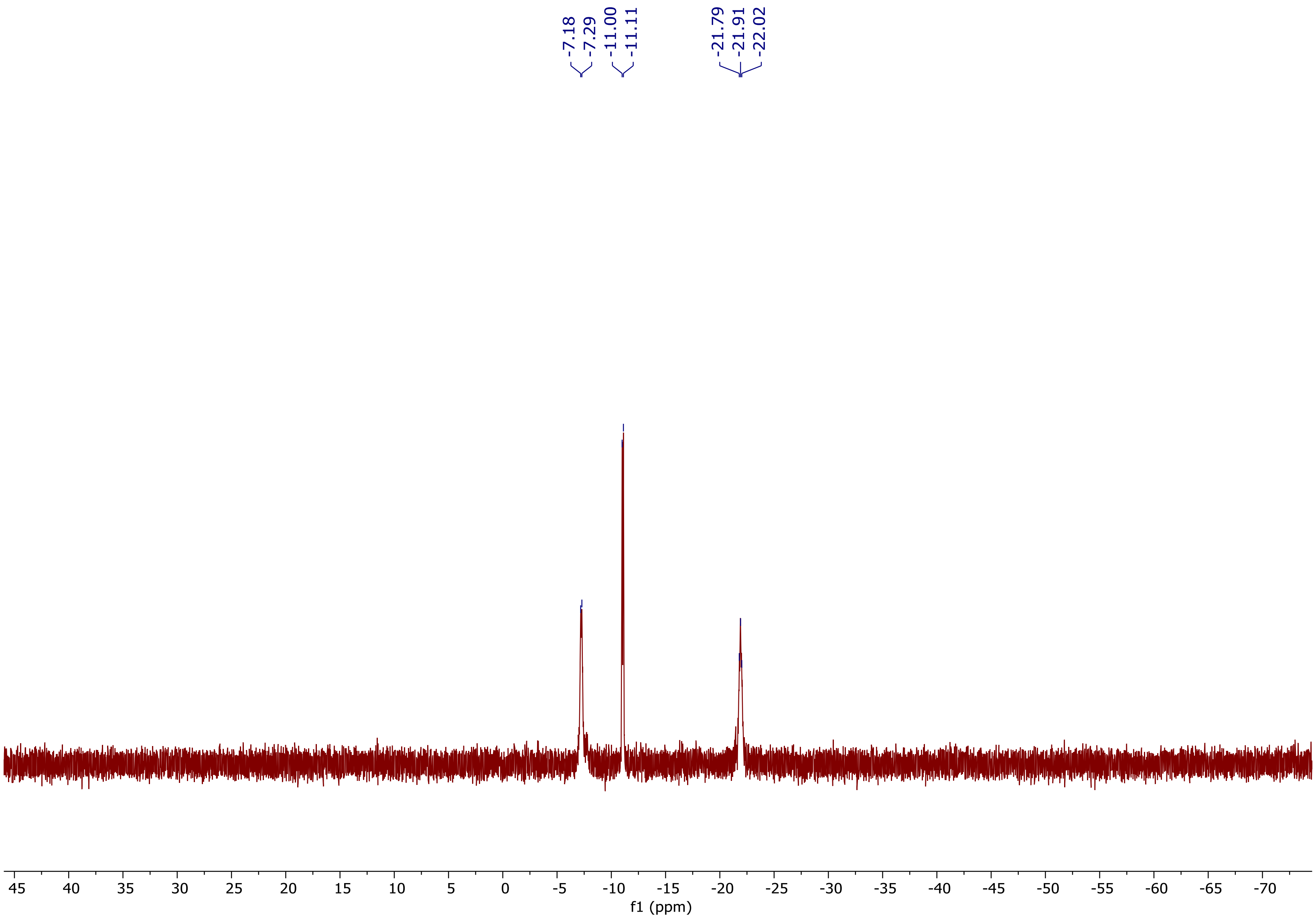

2′MOE ATP: ^{1}H NMR (400 MHz, D_2O)

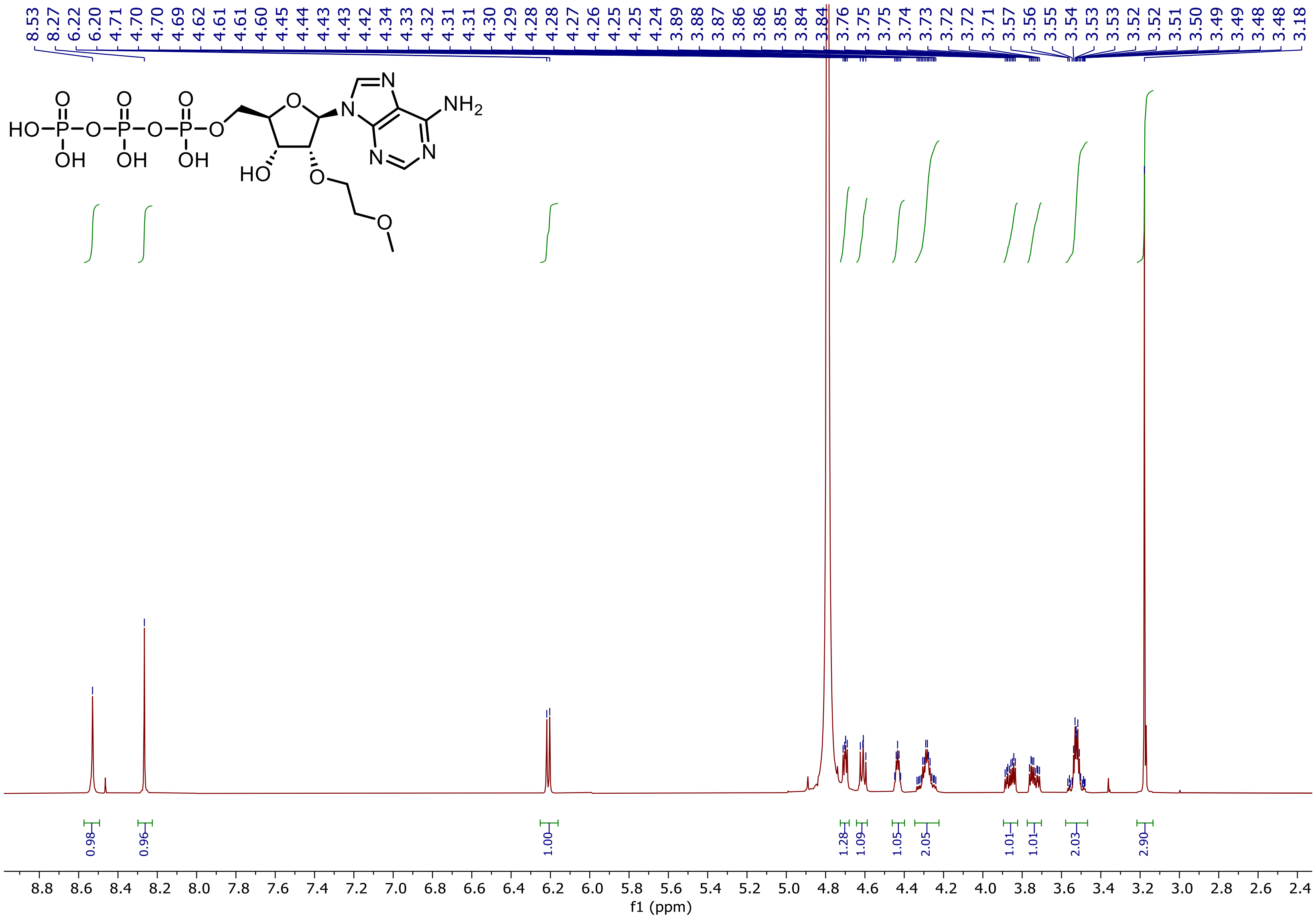

^{13}C NMR (101 MHz, D_2O)

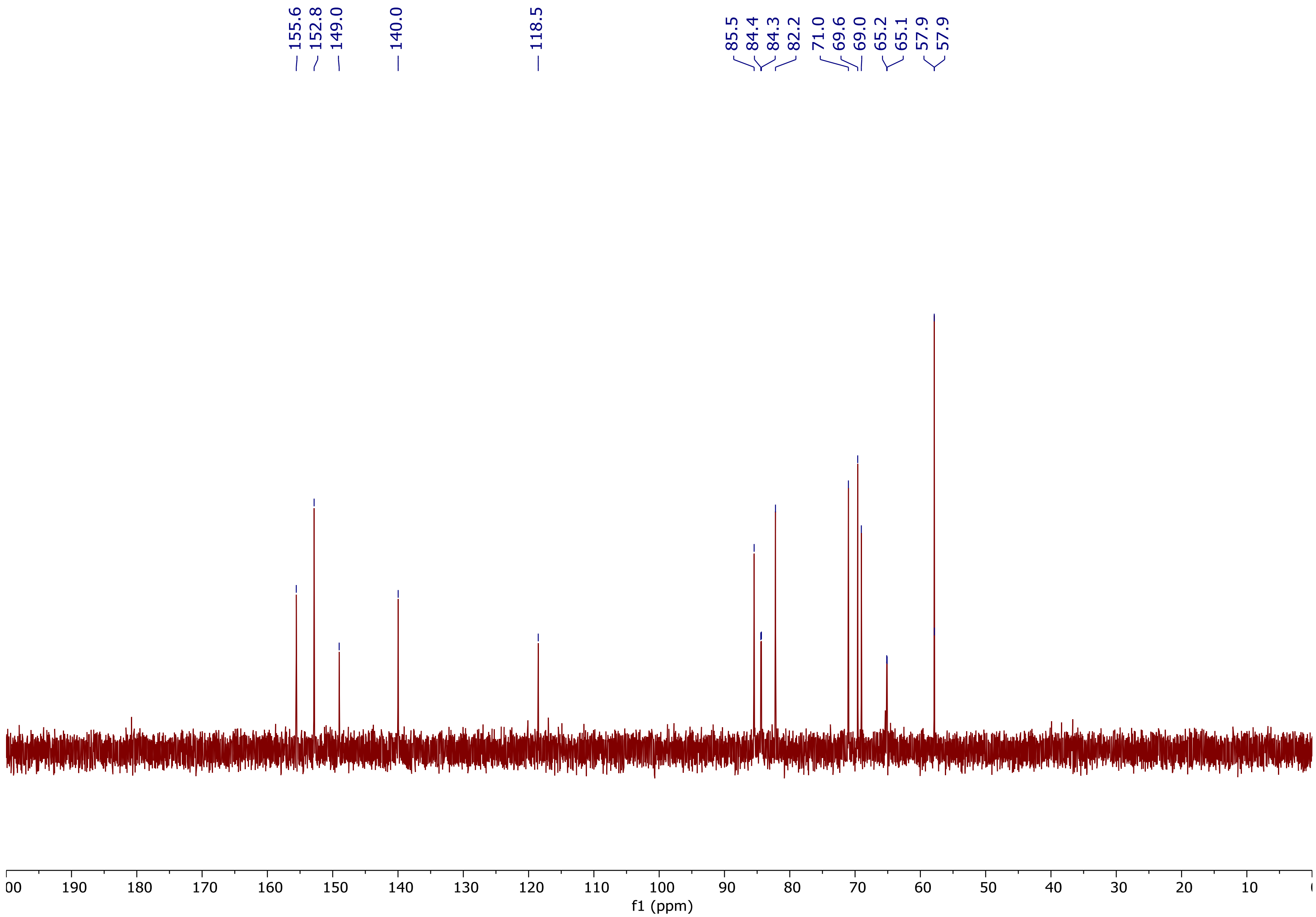

^{31}P NMR (162 MHz, D_2O)

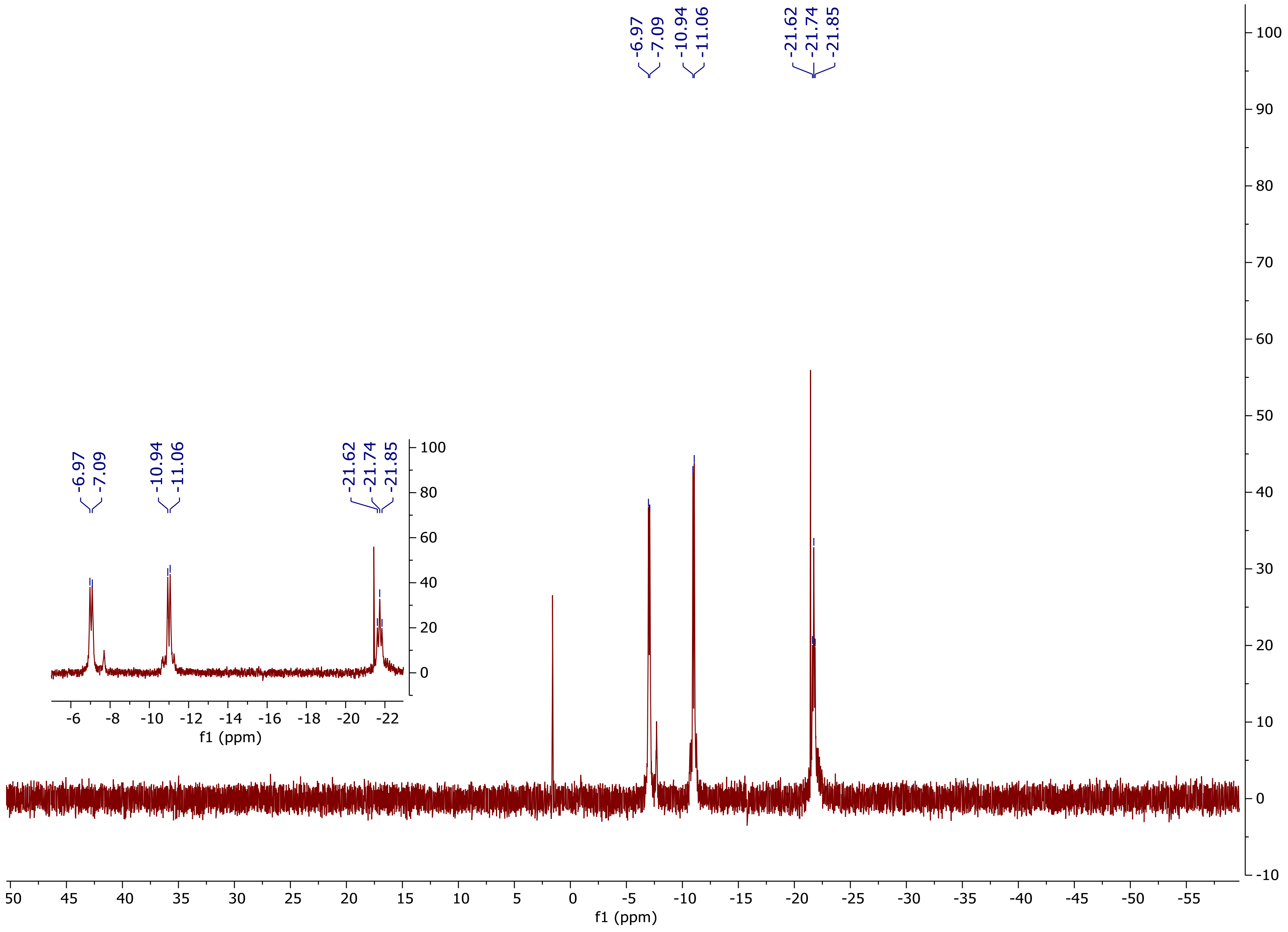

2′F ATP: ^{1}H NMR (400 MHz, D_2O)

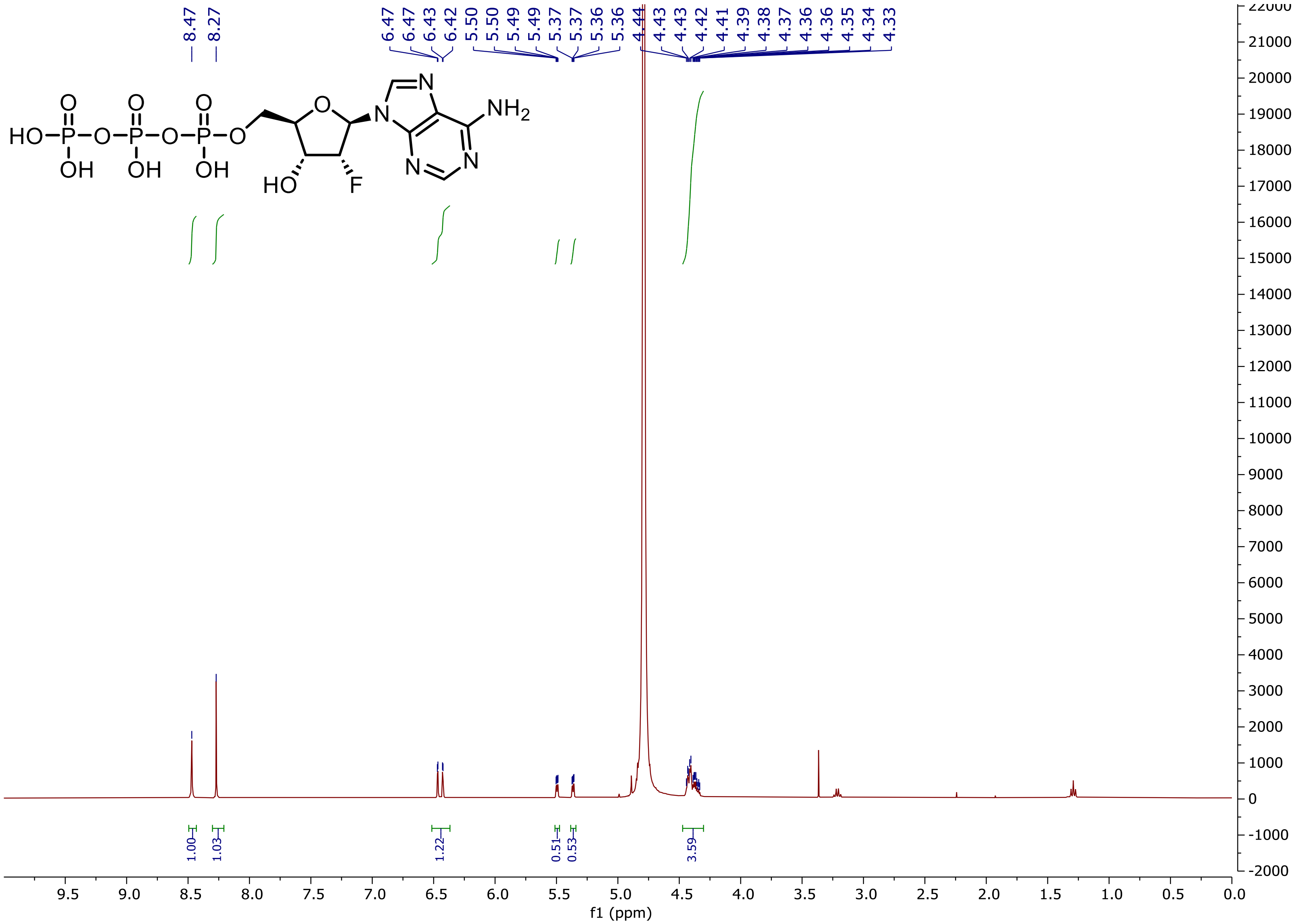

^{13}C NMR (101 MHz, D_2O)

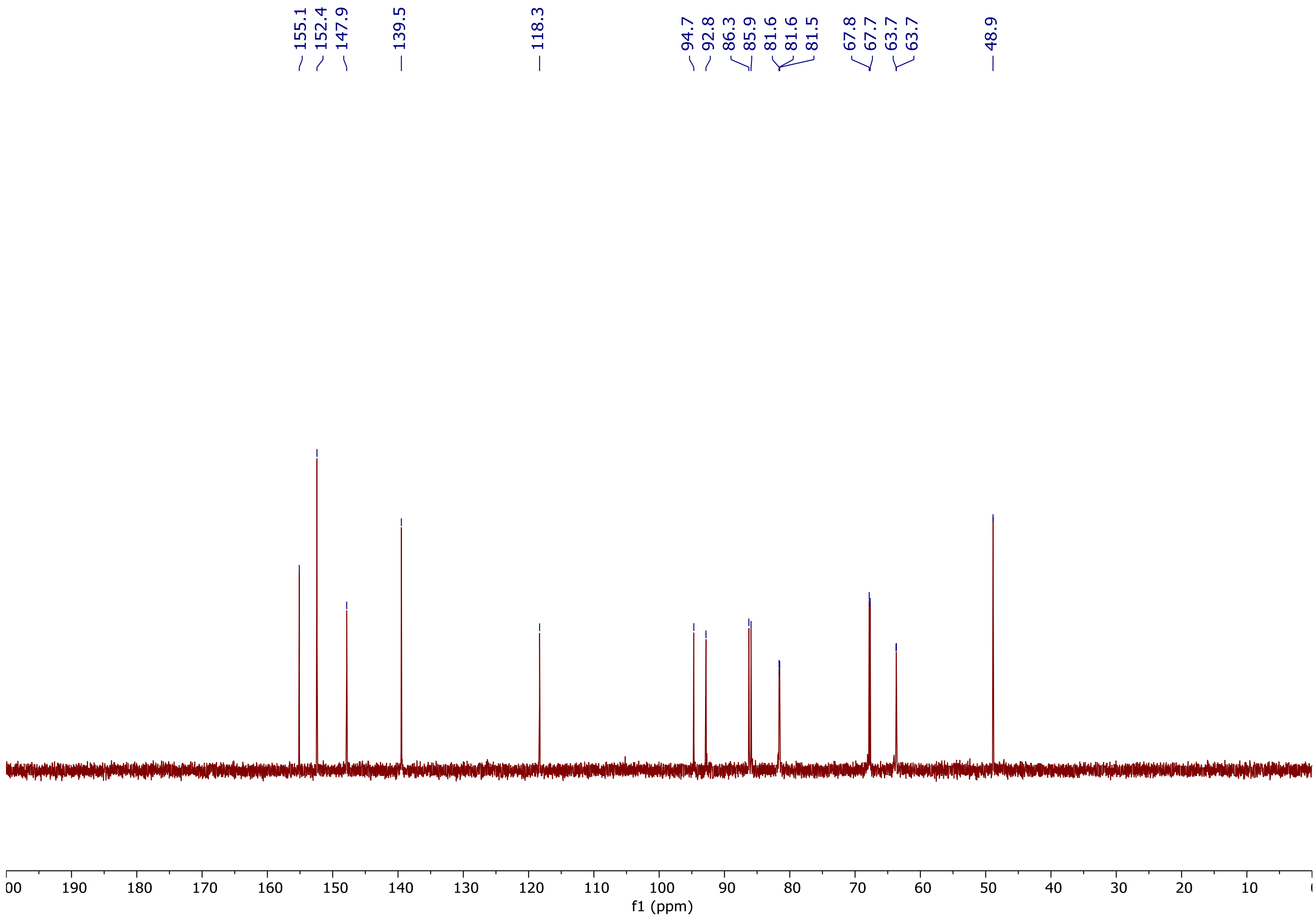

^{19}F NMR (377 MHz, D_2O)

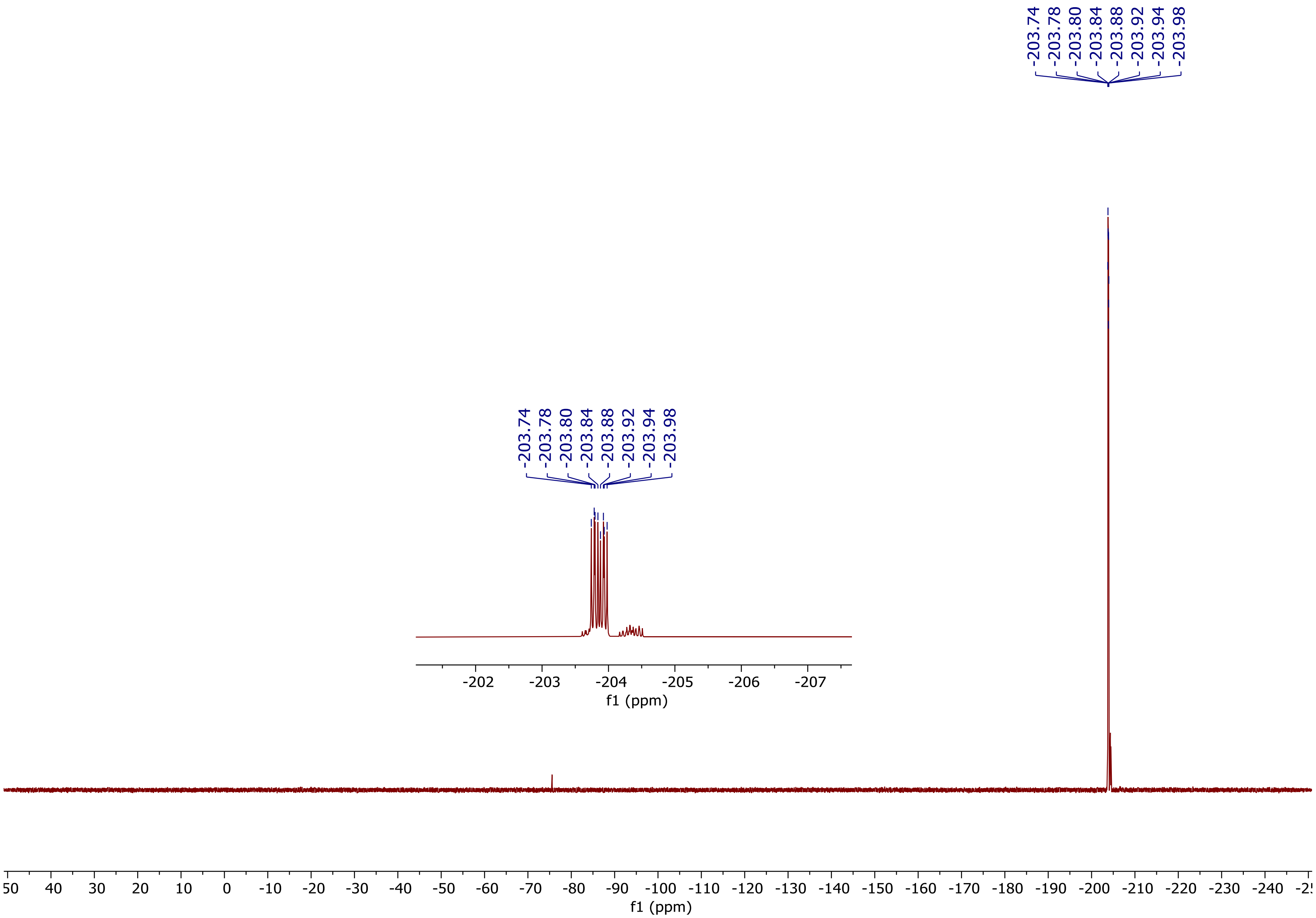

^{31}P NMR (162 MHz, D_2O)

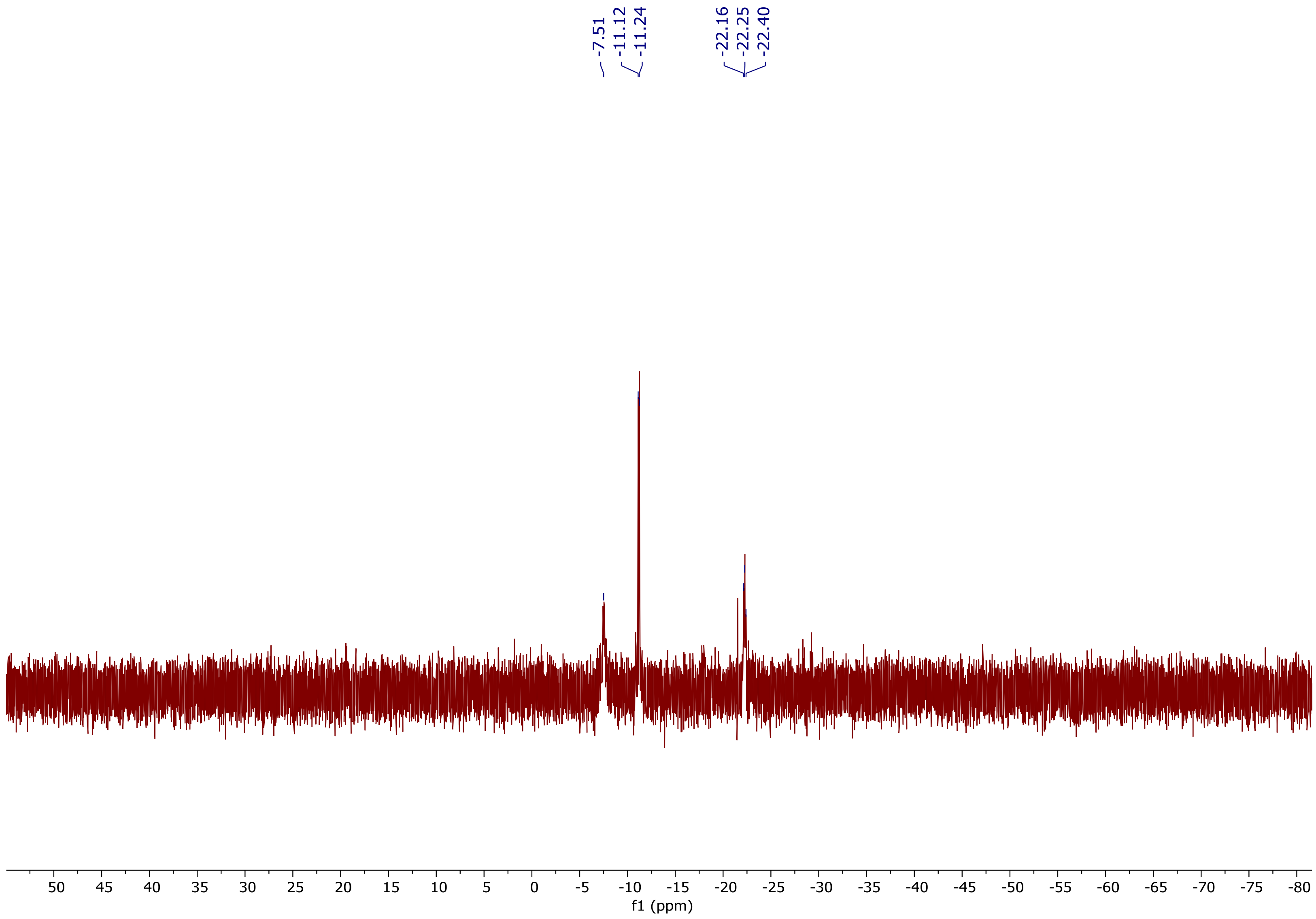

2′F ATPαS: ^{1}H NMR (400 MHz, D_2O)

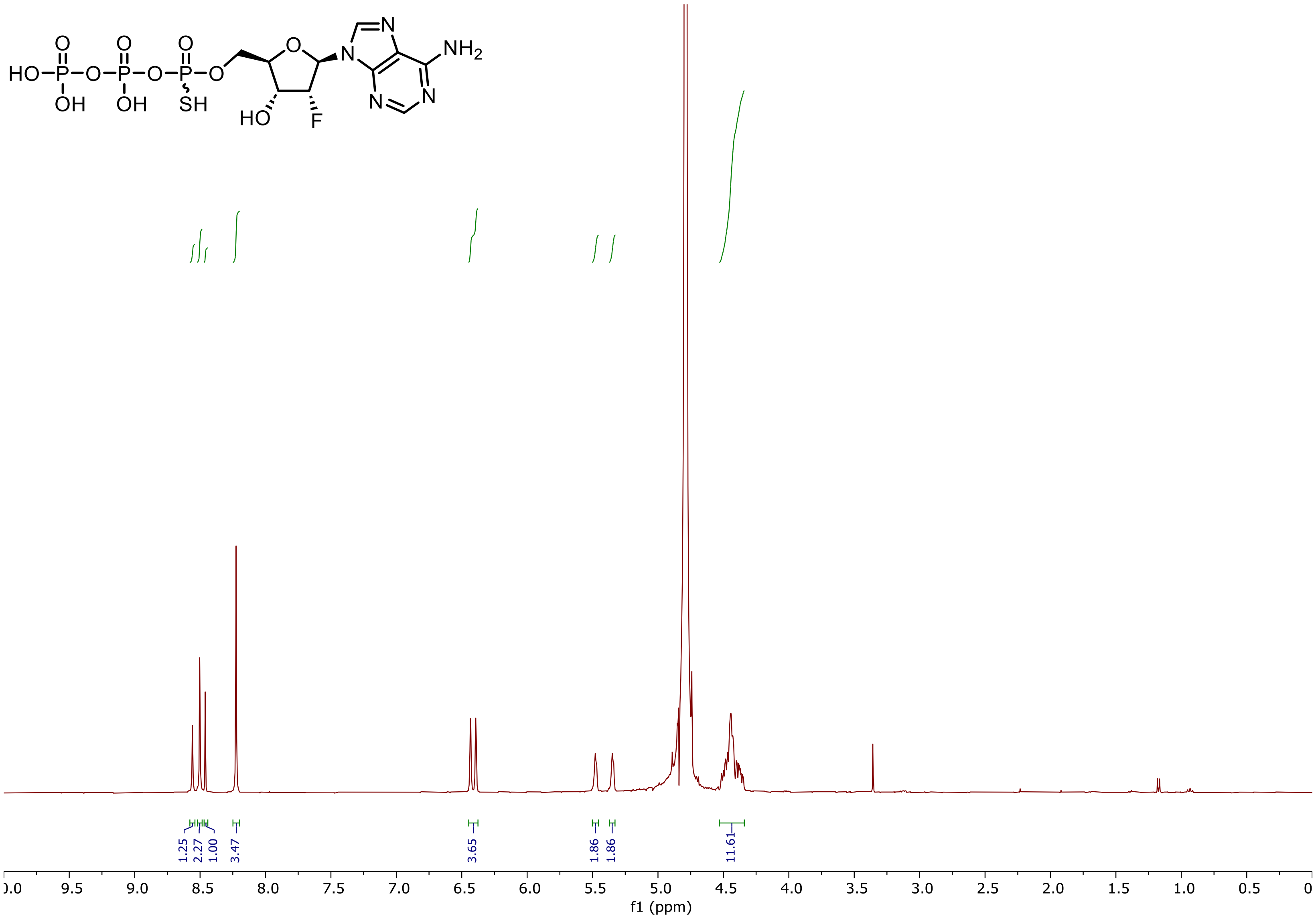

^{13}C NMR (101 MHz, D_2O)

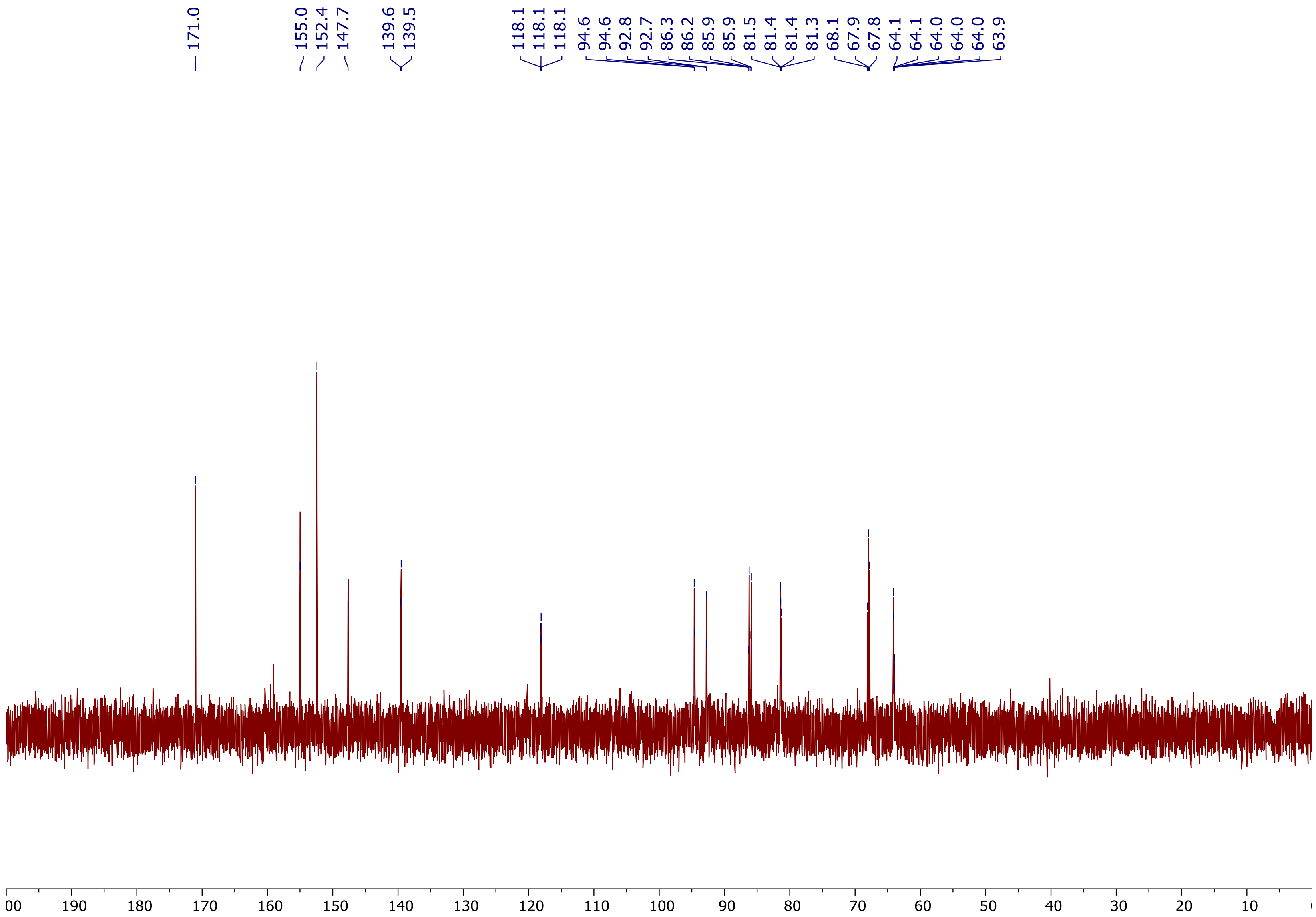

^{19}F NMR (£77 MHz, D_2O)

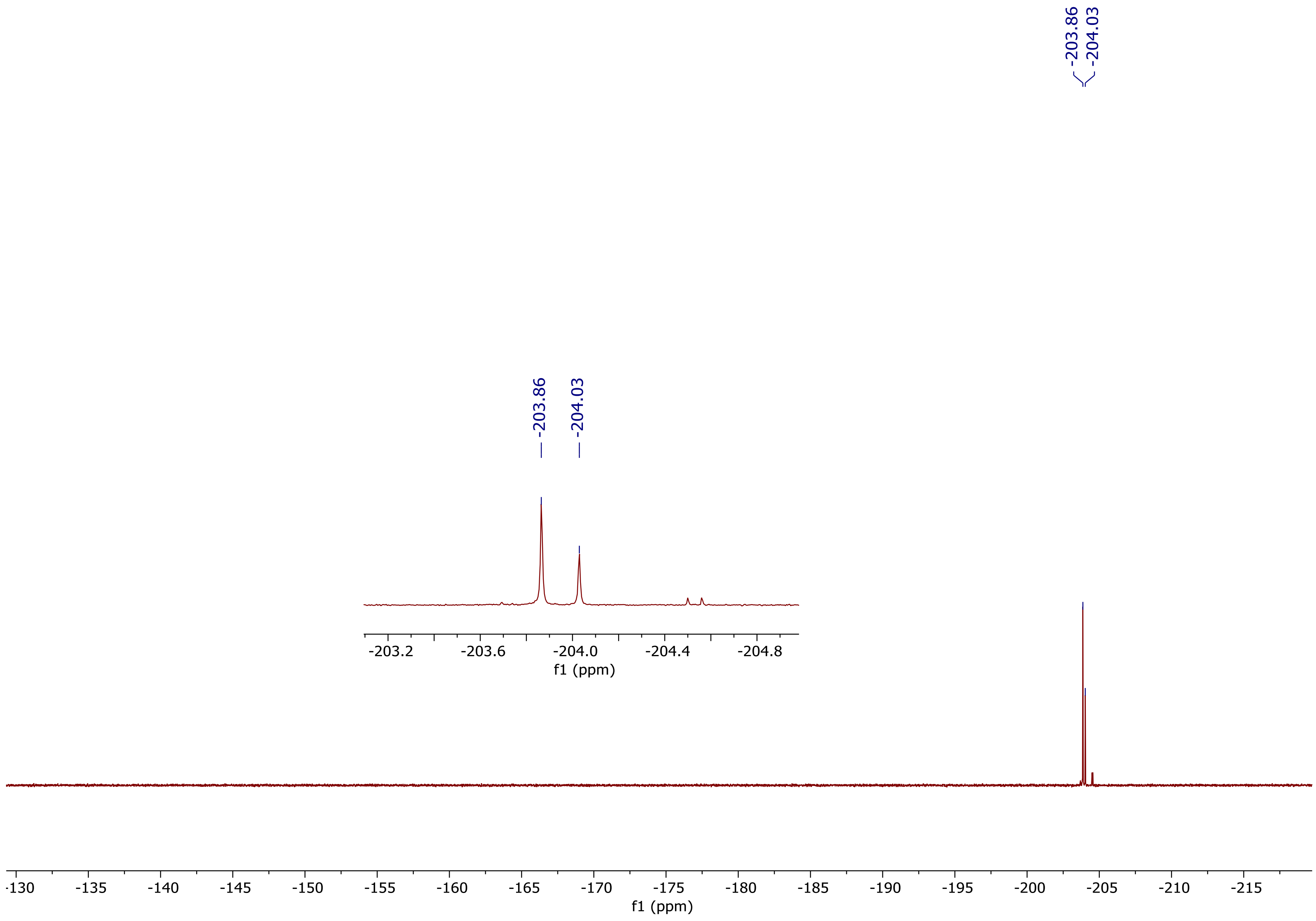

^{31}P NMR (162 MHz, D_2O)

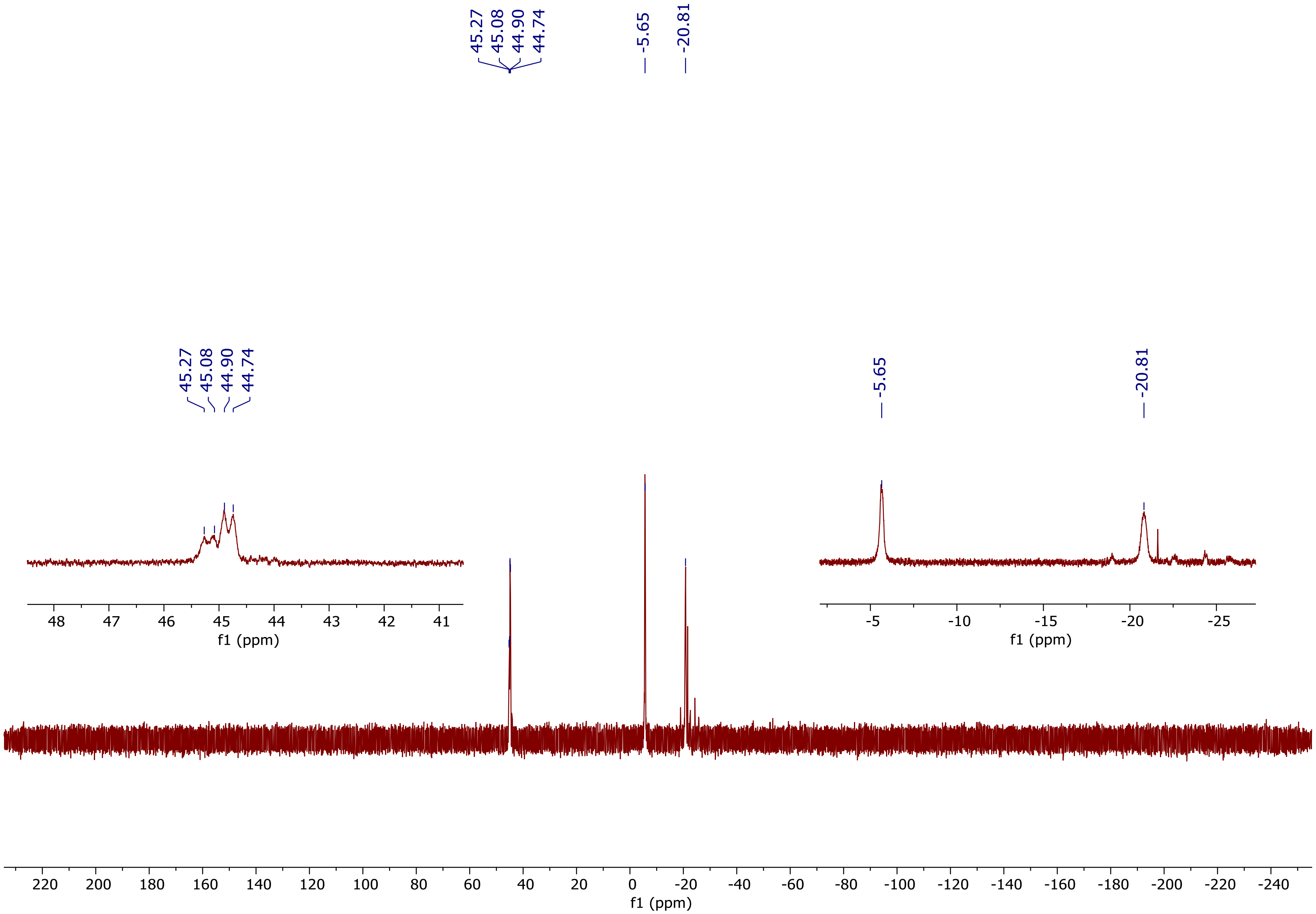

2′OMe TTP: ¹H NMR (400 MHz, D_2O)

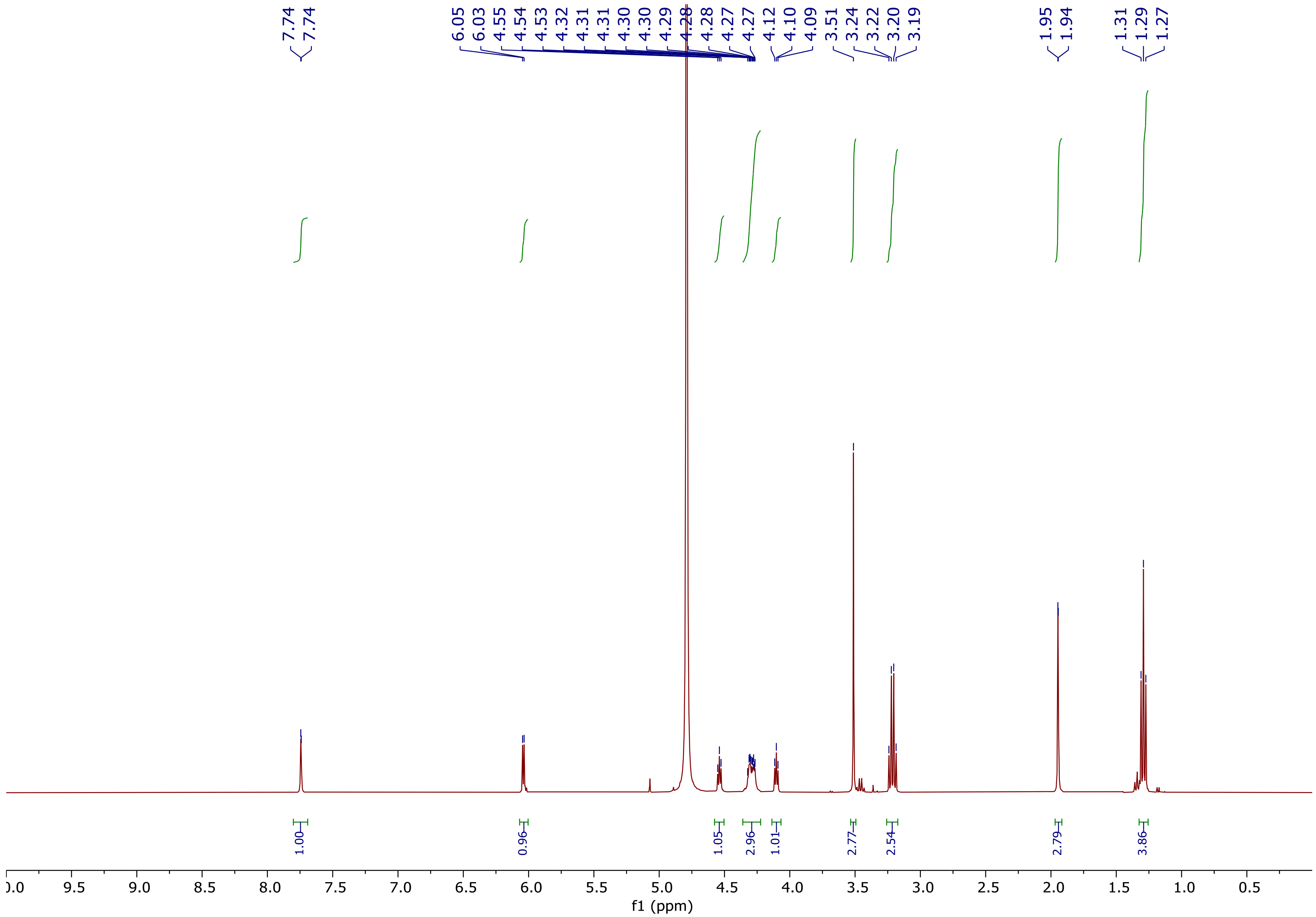

^{13}C NMR (101 MHz, D_2O)

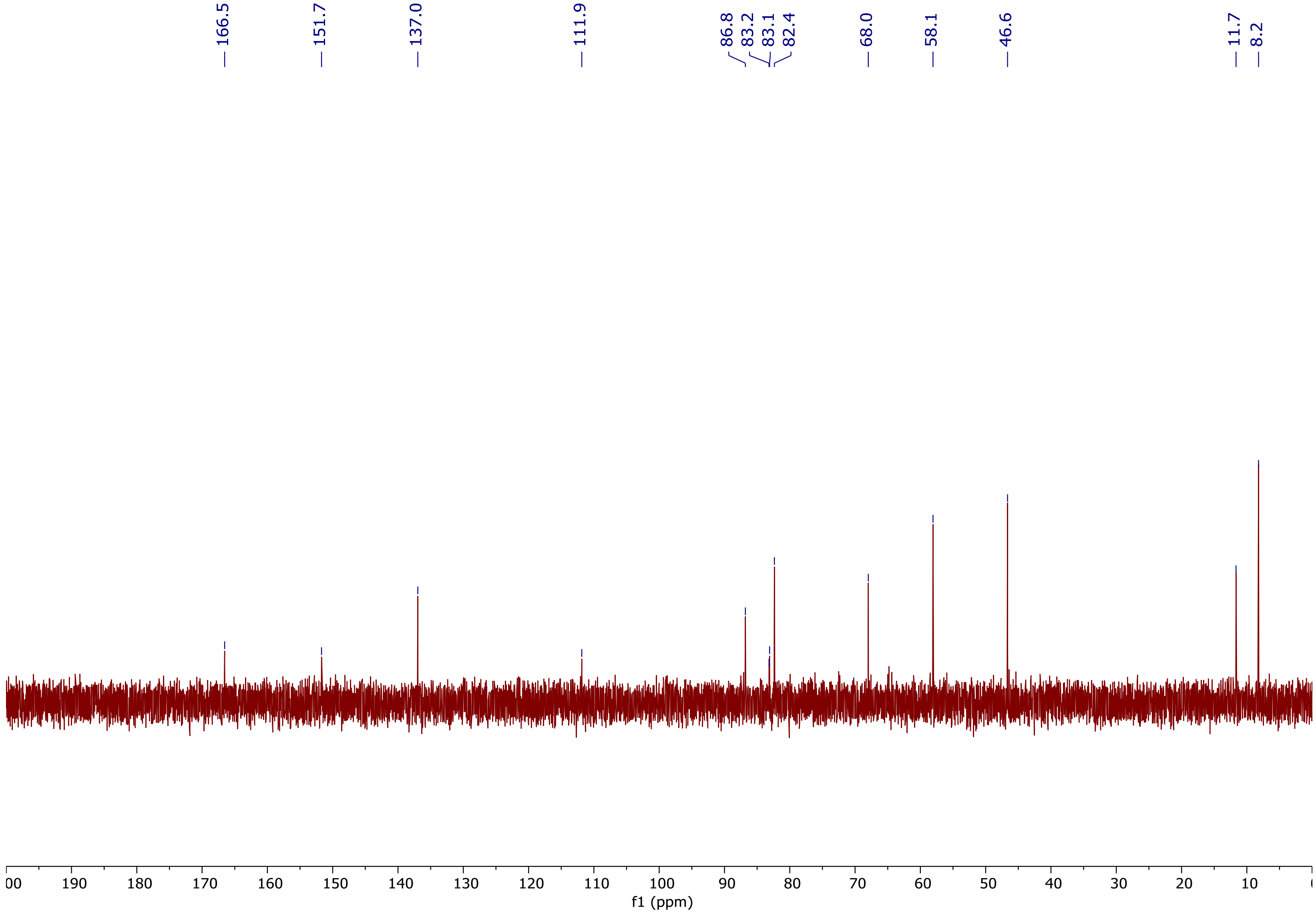

^{31}P NMR (162 MHz, D_2O)

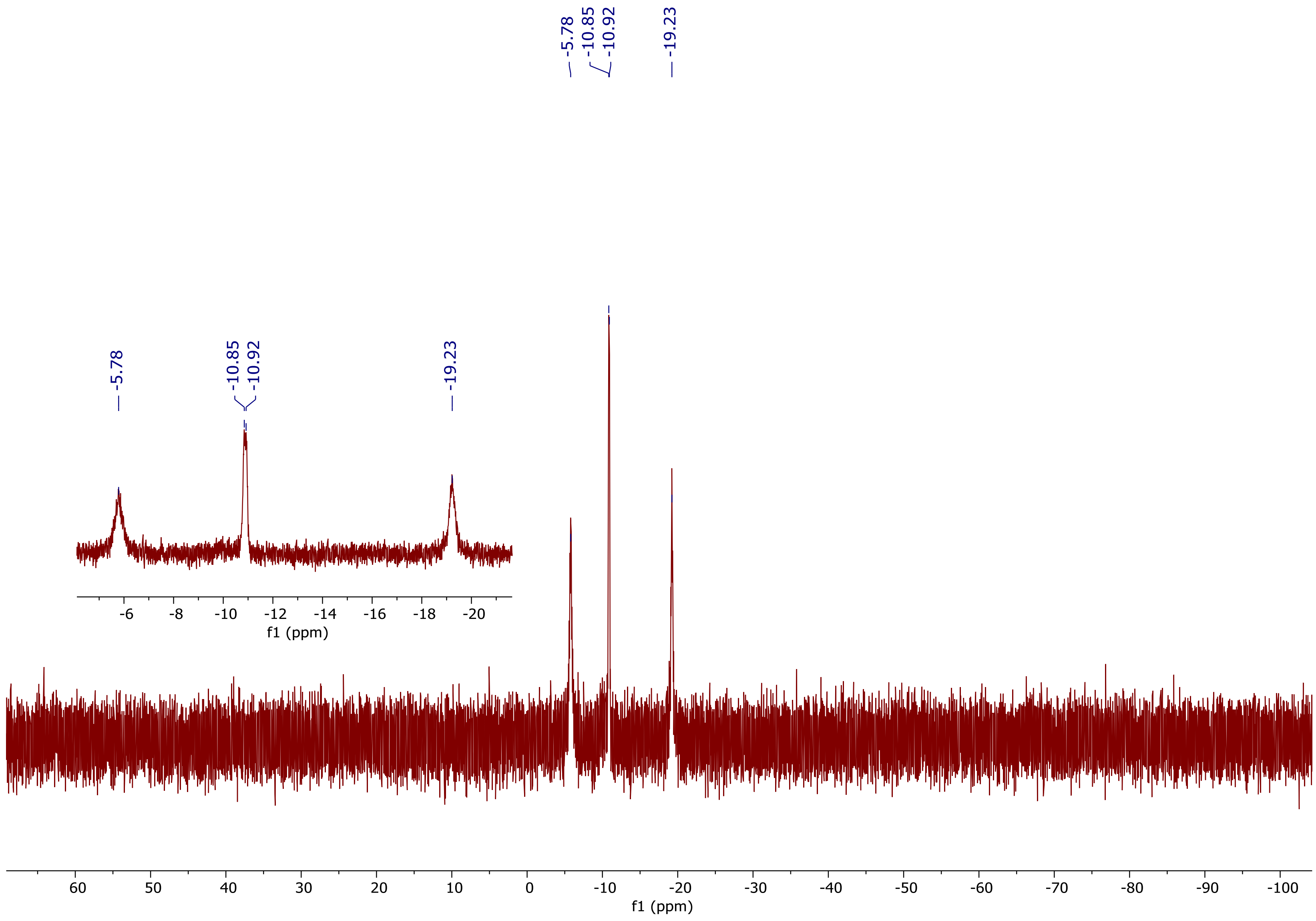

2′MOE TTP: ¹H NMR (400 MHz, D_2O)

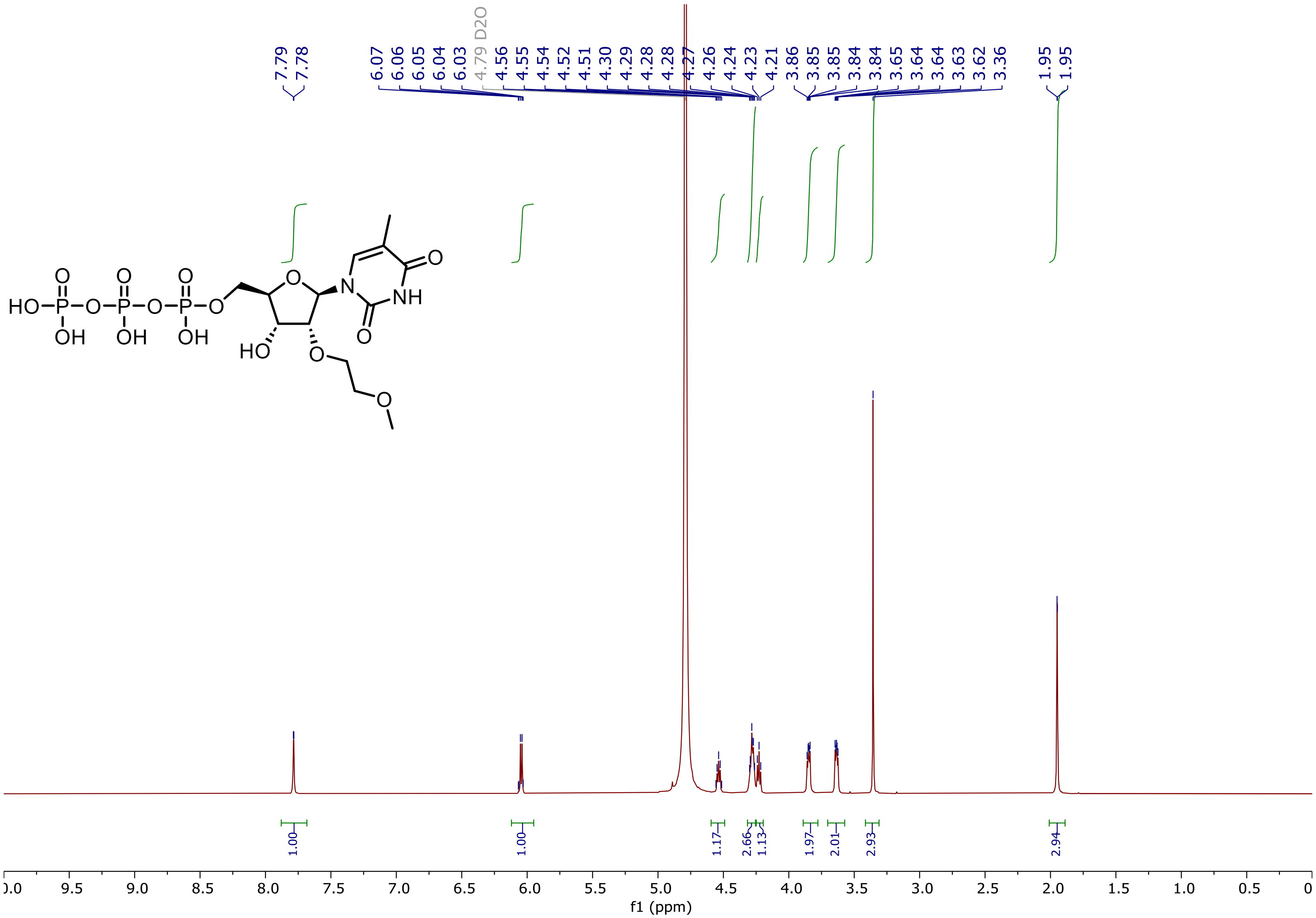

^{13}C NMR (101 MHz, D_2O)

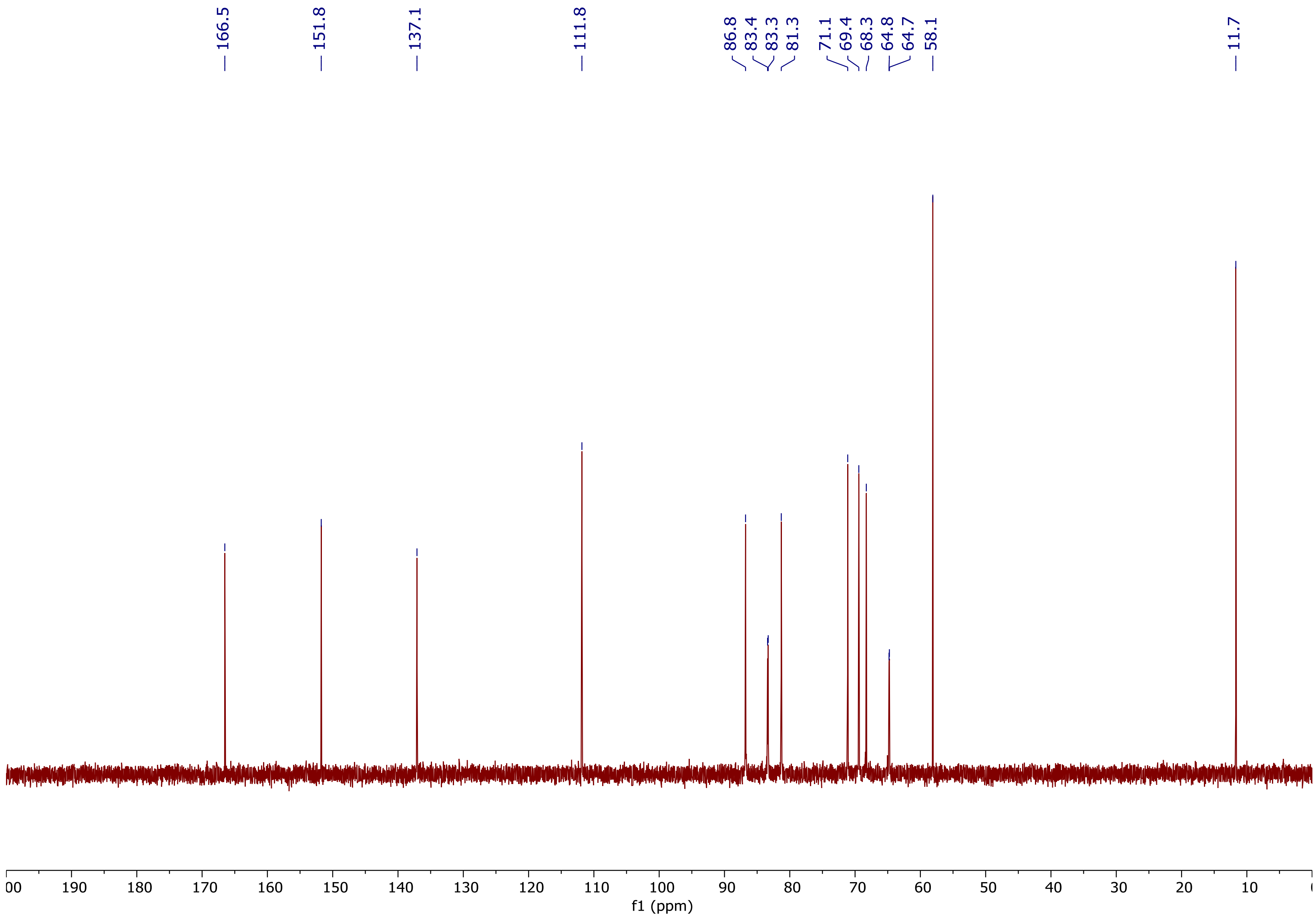

^{31}P NMR (162 MHz, D_2O)

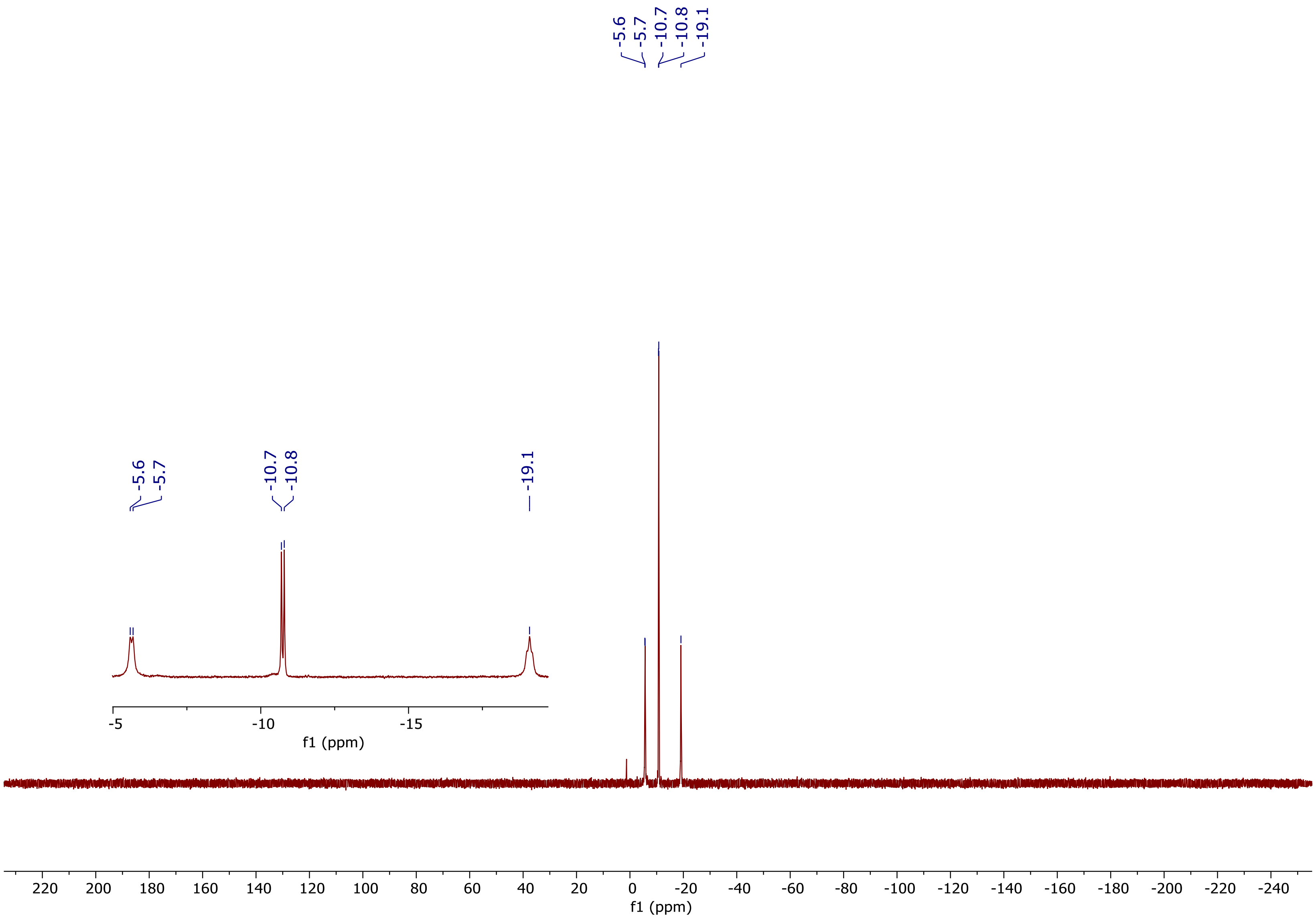

2′F UTP: ¹H NMR (400 MHz, D_2O)

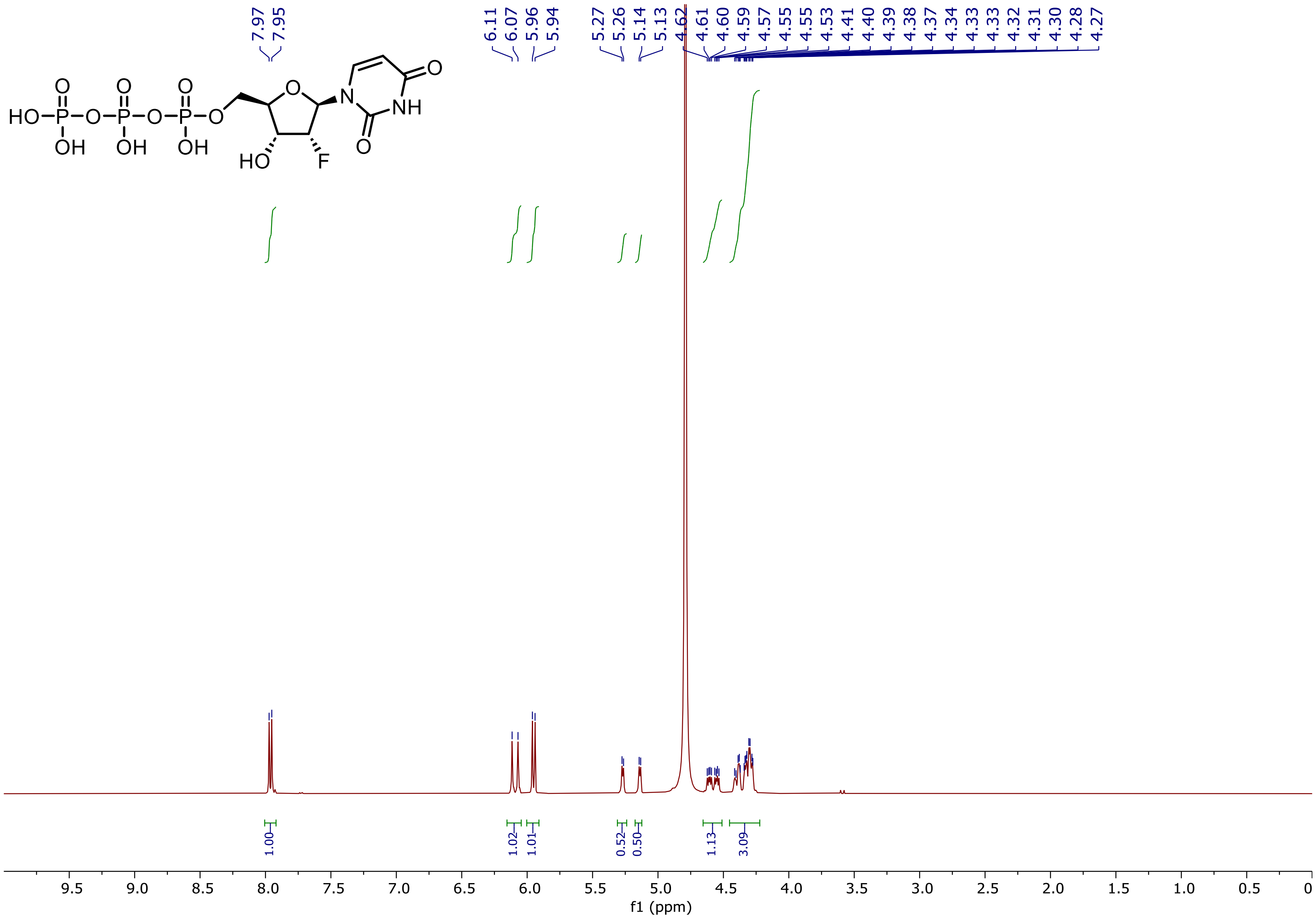

^{13}C NMR (101 MHz, D_2O)

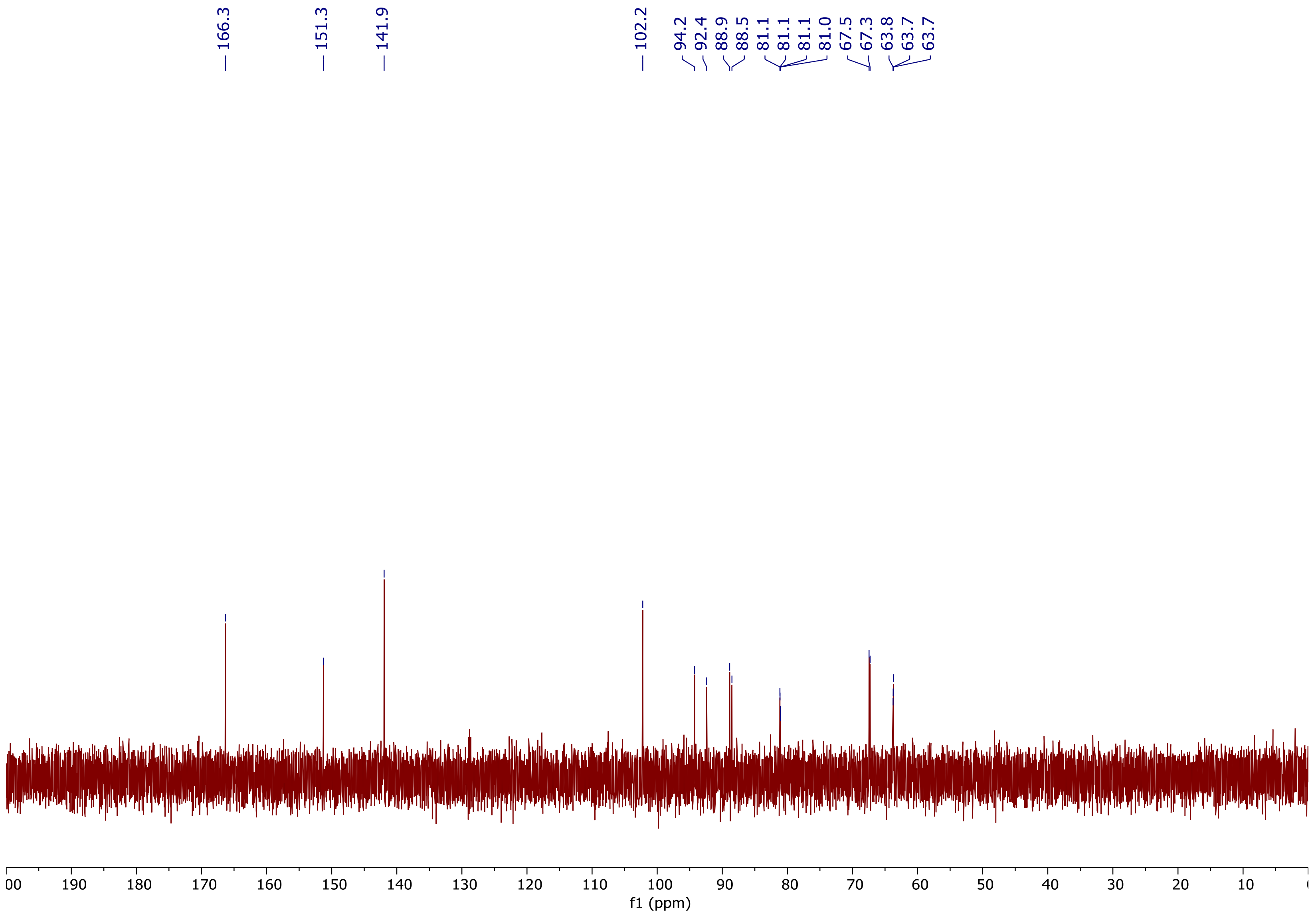

^{19}F NMR (377 MHz, D_2O)

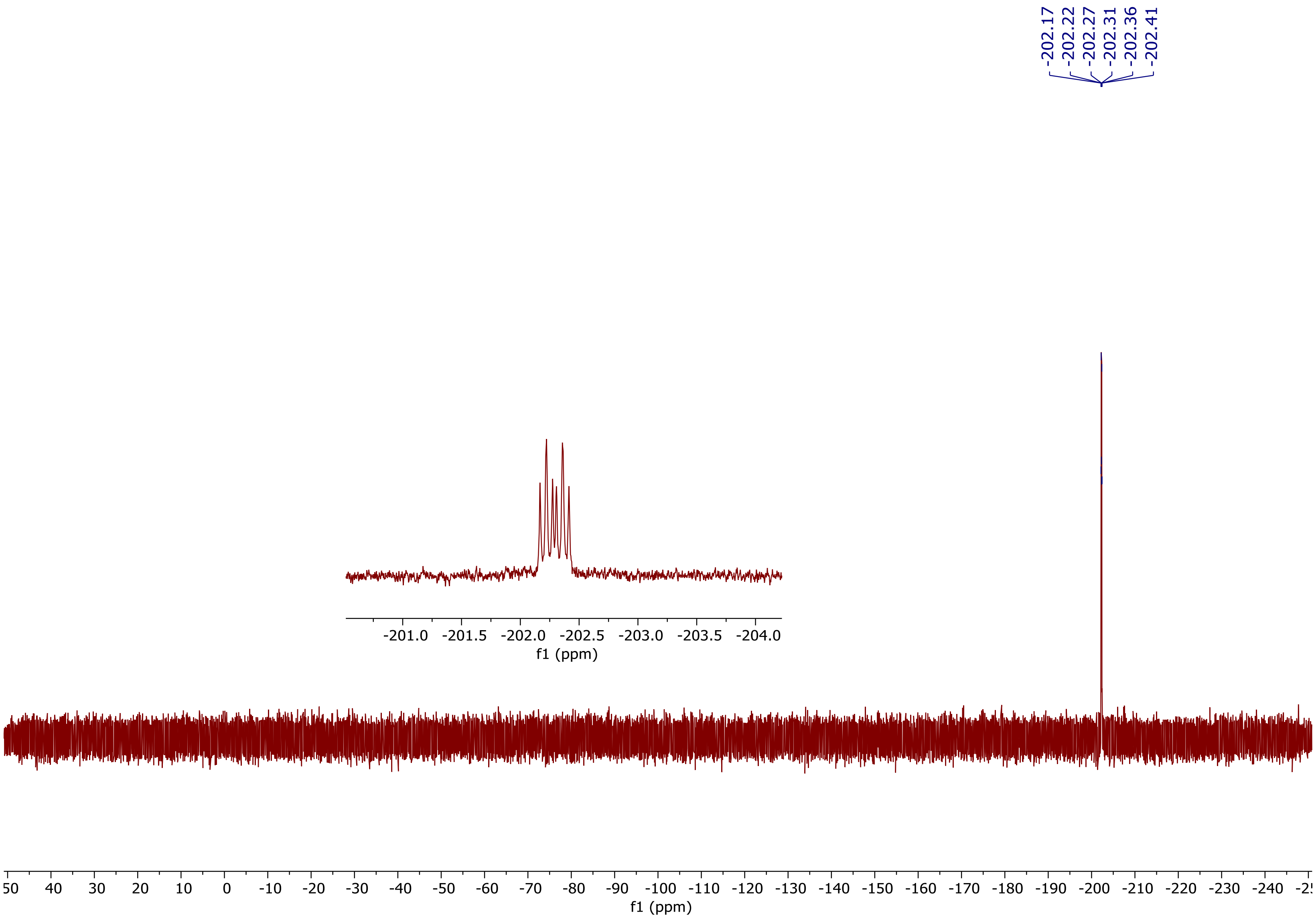

^{31}P NMR (162 MHz, D_2O)

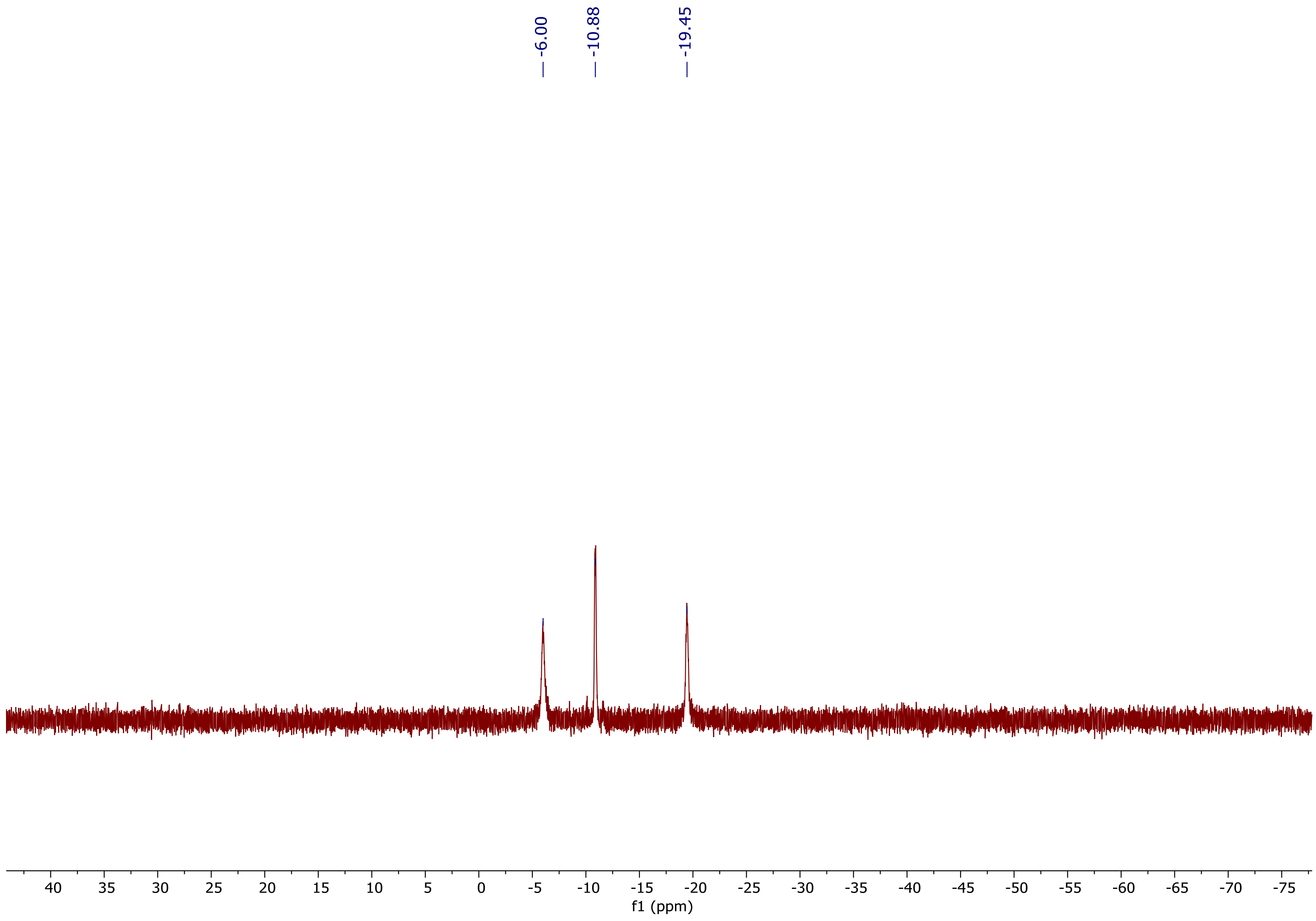

2′OMe GTP: ^{1}H NMR (400 MHz, D_2O)

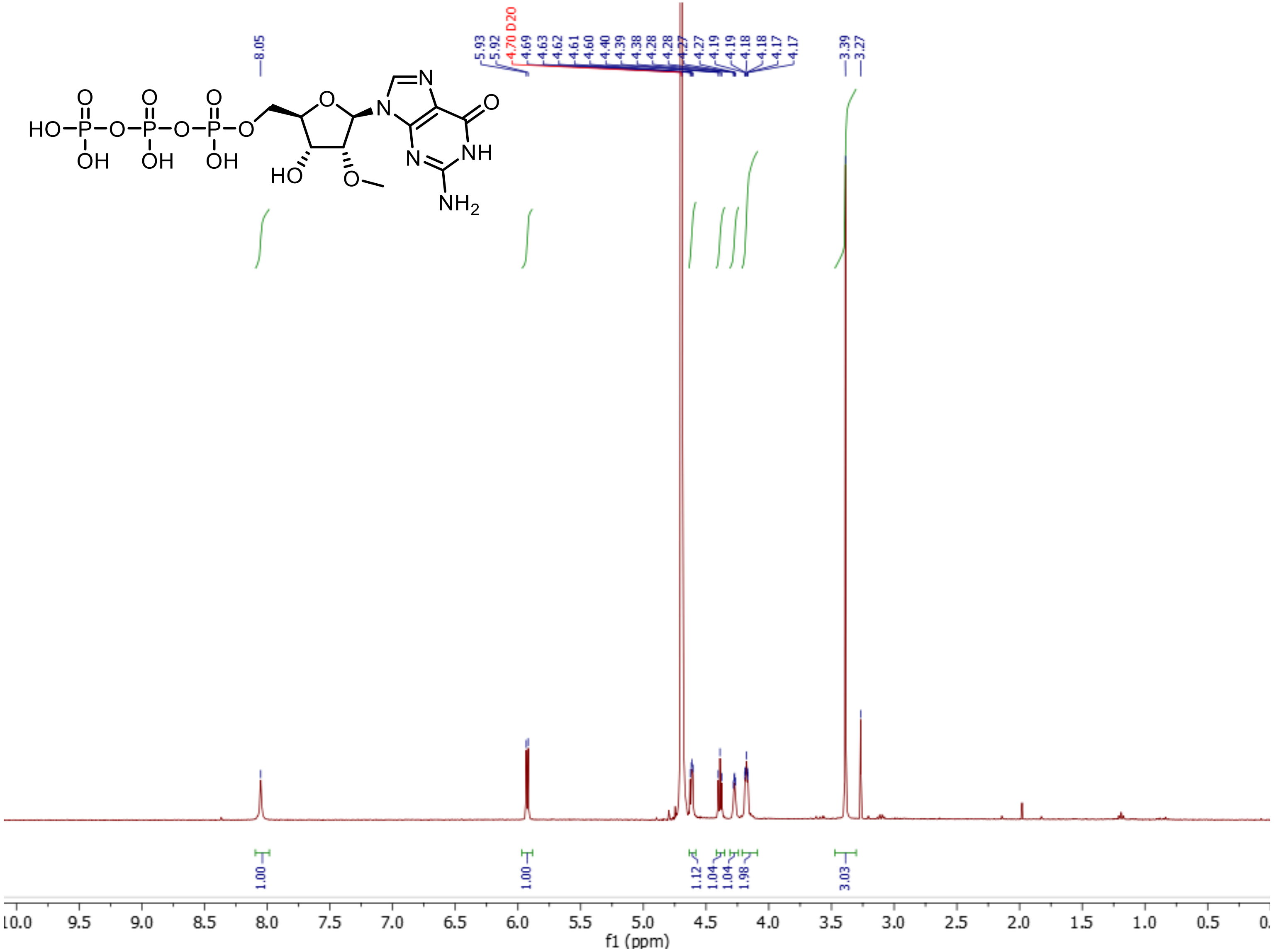

^{13}C NMR (101 MHz, D_2O)

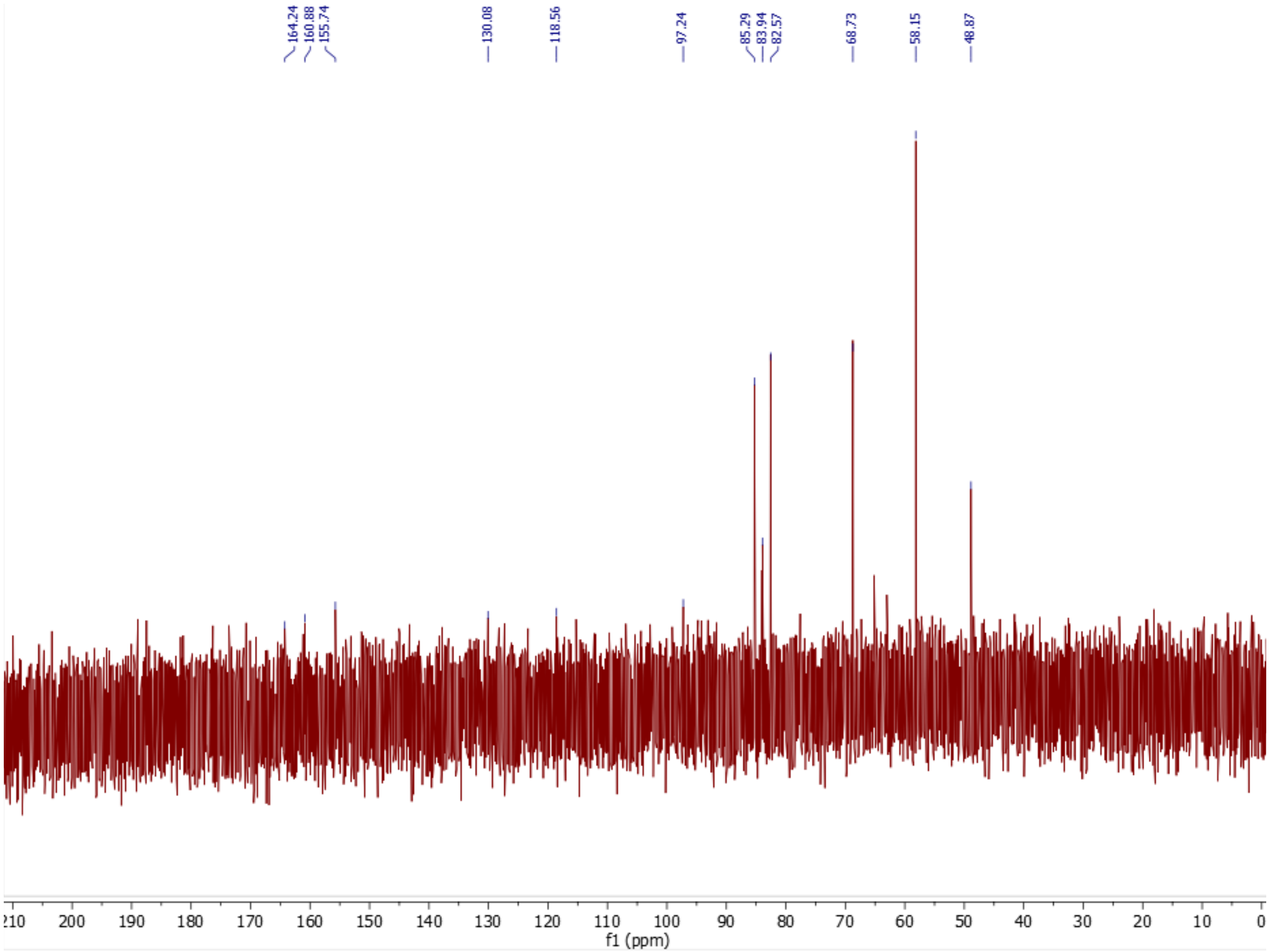

$^{31}P\{^{1}H\}$ NMR (162 MHz, D_2O)

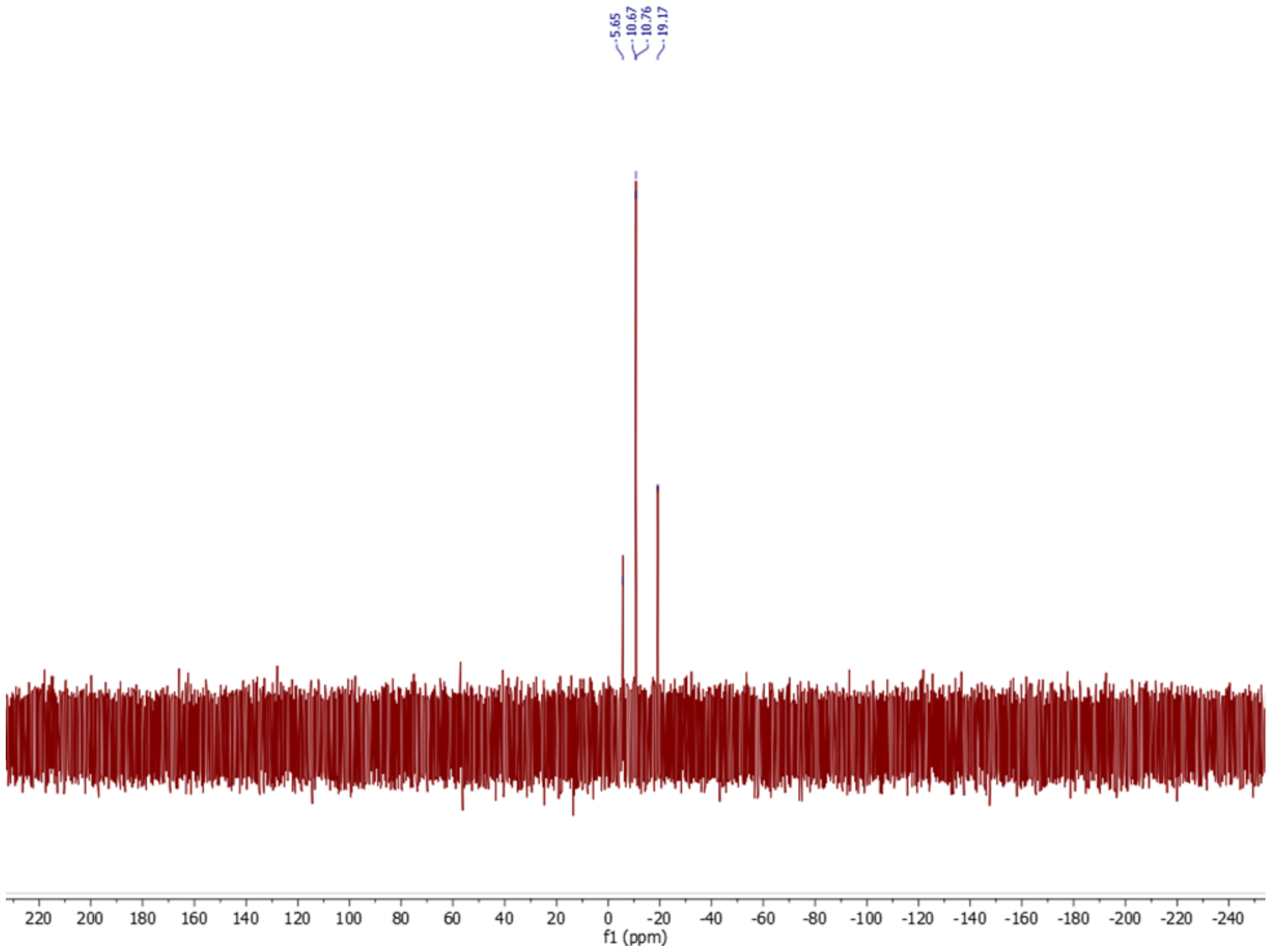

2'OMe CTP: ^{1}H NMR (400 MHz, D_2O)

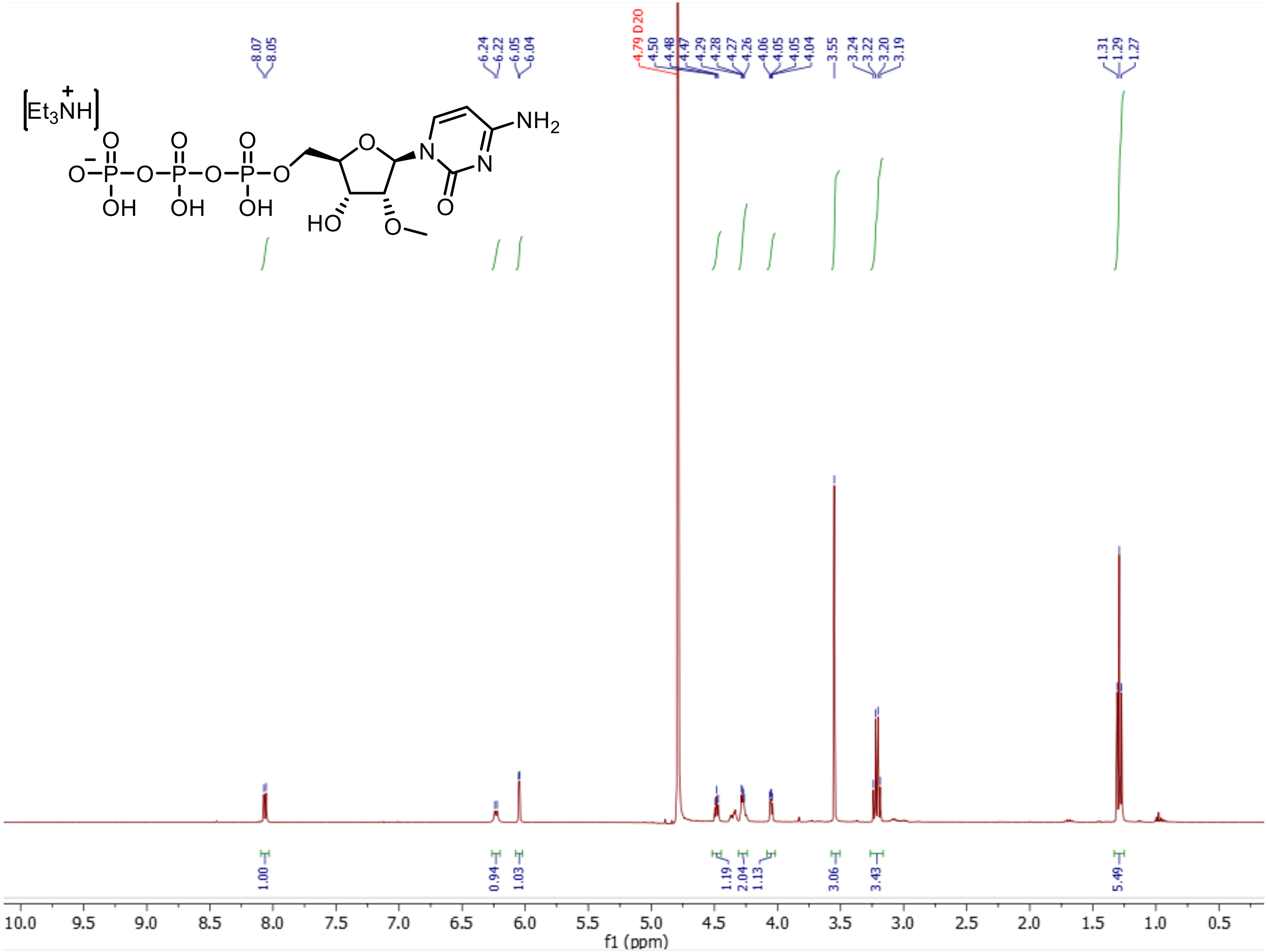

^{13}C NMR (101 MHz, D$_2$O)

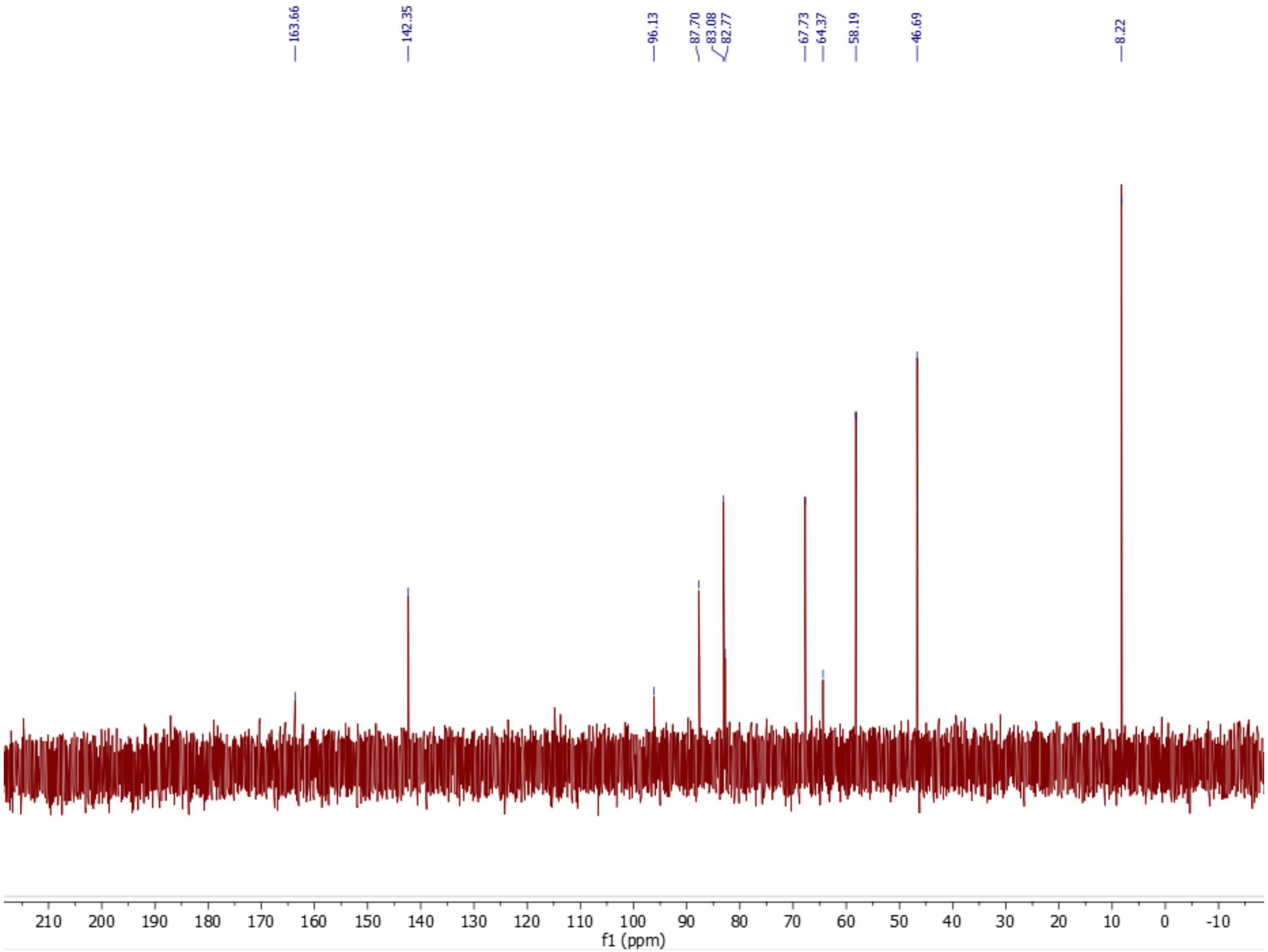

$^{31}P\{^{1}H\}$ NMR (162 MHz, D_2O)

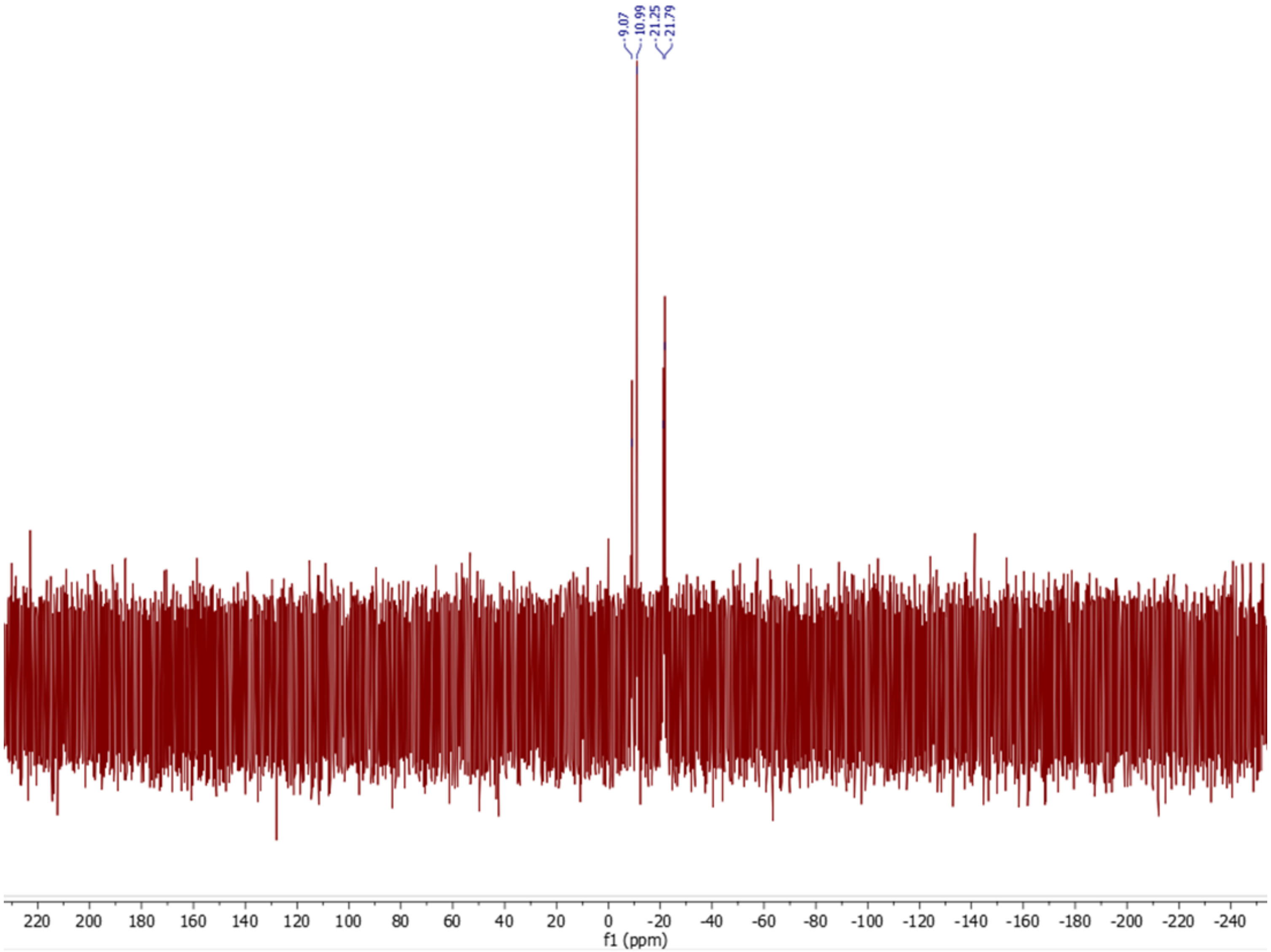

SUPPLEMENTARY REFERENCE

Stothard, P. (2000) The sequence manipulation suite: JavaScript programs for analyzing and formatting protein and DNA sequences. *BioTechniques*, **28**, 1102, 1104.
